# Hemoglobin signal network mapping reveals novel indicators for precision medicine

**DOI:** 10.1038/s41598-023-43694-7

**Published:** 2023-10-25

**Authors:** Randall L. Barbour, Harry L. Graber

**Affiliations:** 1 Department of Pathology, SUNY Downstate Health Sciences University, 450 Clarkson Avenue, Brooklyn, NY 11203 USA; 2 Photon Migration Technologies Corp, 15 Cherry Lane, Glen Head, NY 11545 USA

**Keywords:** Biomarkers, Computational biology and bioinformatics, Data processing, Network topology, Cancer, Tumour biomarkers

## Abstract

Precision medicine currently relies on a mix of deep phenotyping strategies to guide more individualized healthcare. Despite being widely available and information-rich, physiological time-series measures are often overlooked as a resource to extend insights gained from such measures. Here we have explored resting-state hemoglobin measures applied to intact whole breasts for two subject groups – women with confirmed breast cancer and control subjects – with the goal of achieving a more detailed assessment of the cancer phenotype from a non-invasive measure. Invoked is a novel ordinal partition network method applied to multivariate measures that generates a Markov chain, thereby providing access to quantitative descriptions of short-term dynamics in the form of several classes of adjacency matrices. Exploration of these and their associated co-dependent behaviors unexpectedly reveals features of structured dynamics, some of which are shown to exhibit enzyme-like behaviors and sensitivity to recognized molecular markers of disease. Thus, findings obtained strongly indicate that despite the use of a macroscale sensing method, features more typical of molecular-cellular processes can be identified. Discussed are factors unique to our approach that favor a deeper depiction of tissue phenotypes, its extension to other forms of physiological time-series measures, and its expected utility to advance goals of precision medicine.

## Introduction

Underlying the Precision Medicine Initiative is the premise that features of molecular expression are closely tied to disease subgroups^1^, and that applying individualized knowledge of these features will enhance understandings of disease susceptibility and treatment strategies. This focus has motivated the adoption of deep phenotyping strategies, of which multi-omics measures are considered a principal tool^2,3^. While informative, it has been recognized that often not leveraged by this initiative are varied forms of physiological recordings^4,5^. This is unfortunate, as this class of measures is recognized as information-rich as a consequence of sensitivity to a host of ongoing feedback adjustments by unseen drivers that support homeostasis and whose details are often disturbed by disease. While not directly observable, these unseen factors nevertheless produce macroscale behaviors affording the potential to identify surrogate signatures that might usefully serve to bridge macroscale behaviors with the unseen microscale phenomenology.

Invoked here is the rationale that identification of such factors can be gained through examination of low-variance measures of short-term behaviors. An everyday example supporting this view is the seemingly hidden driving movements of a marionette when viewed from a distance. Composite adjustment of forces applied to varied strings (i.e., drivers) produce coordinated movements. Now consider observations that are time-delayed compared to the applied force. The greater the time delay, the more difficult it becomes to recognize the actions of drivers. It follows that reducing this delay will improve recognition of features causatively linked to such actions.

In a previous report we demonstrated that access to such low-variance measures can be gained by invoking a particular form of network analysis of time series^6^. Produced is a low-dimensional Markov chain whose recurring States are generated on a time scale similar to fleeting, nonstationary behaviors which, when combined with use of signal averaging over space–time, achieves the desired low-variance measures. When applied to hemoglobin time-series measures collected from women with breast cancer and a control group, revealed were multiple classes of adjacency matrices whose coefficient comparisons showed mainly uncorrelated patterns and disease sensitivity, suggesting that extended sensitivity to a host of unseen disease-sensitive drivers may be achievable^6,7^.

Here we have explored a logical follow-on to these measures and invoke the hypothesis that because multiple adjacency classes are generated, the actions of the considered drivers are likely dispersed across the same. Analogous to a puzzle, coalescing of coefficients across such matrices is taken as equivalent to assembling its pieces, thereby revealing recognizable patterns. To achieve this, we have systematically explored higher-order co-dependencies across such matrices in the hope of identifying trends that may reveal actions of said drivers. As shown herein, the considered examination reveals several forms of novel behavior that, unexpectedly, have a highly structured character and are exemplary of features more typical of molecular-cellular processes. Also revealed are instances of hidden behaviors whose features are dependent on network-coefficient patterns not evident from inspection, some of which are shown sensitive to well-known molecular markers used to guide treatments.

## Results

### Mapping of continuous voxel time series signals into discrete Hb states

**Figure 1:**
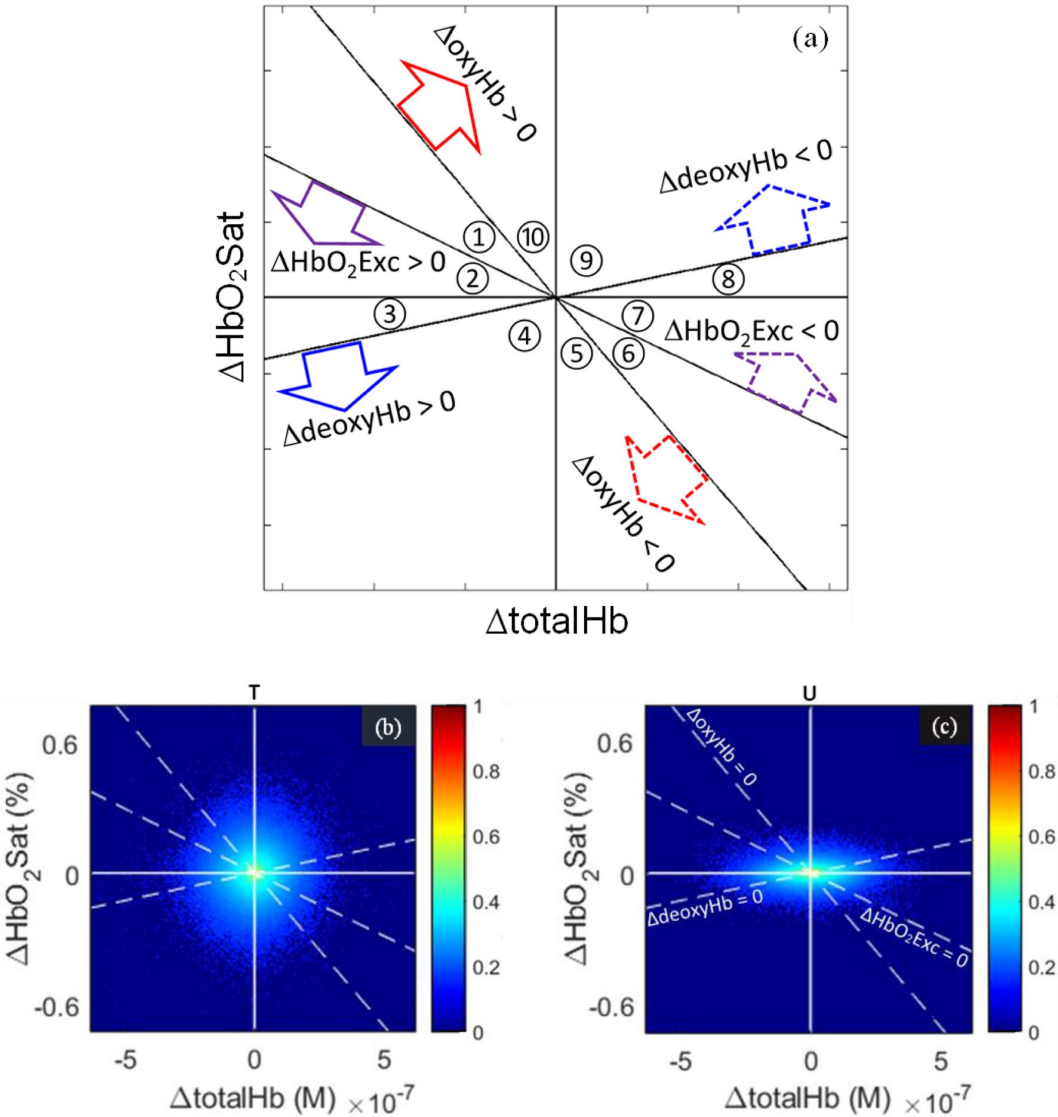
Mapping of continuous time-series signals into discrete Hb states. (**a**) Each Hb State is a region corresponding to one of the ten physiologically allowed permutations of algebraic signs of the five components of the Hb signal, depicted as regions in the ΔtotalHb-ΔHbO_2_Sat coordinate system (circled numbers are State assignments). Each secondary axis is the locus of points at which one of the other three components is equal to its baseline mean value (e.g., ΔdeoxyHb = 0 on the positively sloped axis). Colored arrows indicate which regions of the plane correspond to positive (solid-line arrows) and to negative (dashed-line arrows) deviations. (**b**–**c**) Paired resting-state ΔtotalHb and ΔHbO_2_Sat values for affected (**b**) and unaffected (**c**) breasts of a selected breast-cancer subject (50 y/o, BMI = 44, 4 cm intraductal carcinoma). Represented on the color axis is the (occurrence percentage)^1/7^ of temporally coincident resting-state ΔHbO_2_Sat and ΔtotalHb image values; the 7-th root, having a similar effect to logarithmic scaling, is plotted for ease of visualization.

Shown in Fig. 1a is the coordinate system used to define finite-state behaviors of the Hb signal from which network measures are derived. Orientations of the secondary axes relative to the primary ΔtotalHb and ΔHbO_2_Sat axes are determined by mathematical dependencies among the signal components (Supplementary Note 1).

A portion of the input data from which network measures are derived is shown in Fig. 1b,c. Plotted is the percentage of ΔHbO_2_Sat and ΔtotalHb values observed during the resting state for a representative subject. Seen are cloud-like distributions, with a span of values along the HbO_2_Sat axis for the tumor-bearing breast (T) exaggerated compared to the unaffected breast (U), suggesting that blood-volume adjustments maintain HbO_2_Sat within narrow limits in U but fail to exert a similar controlling effect in T. Apart from this gross feature, there is no evidence of structure within these distributions.

### Transition-type patterns and disease sensitivities of network coefficients

**Figure 2:**
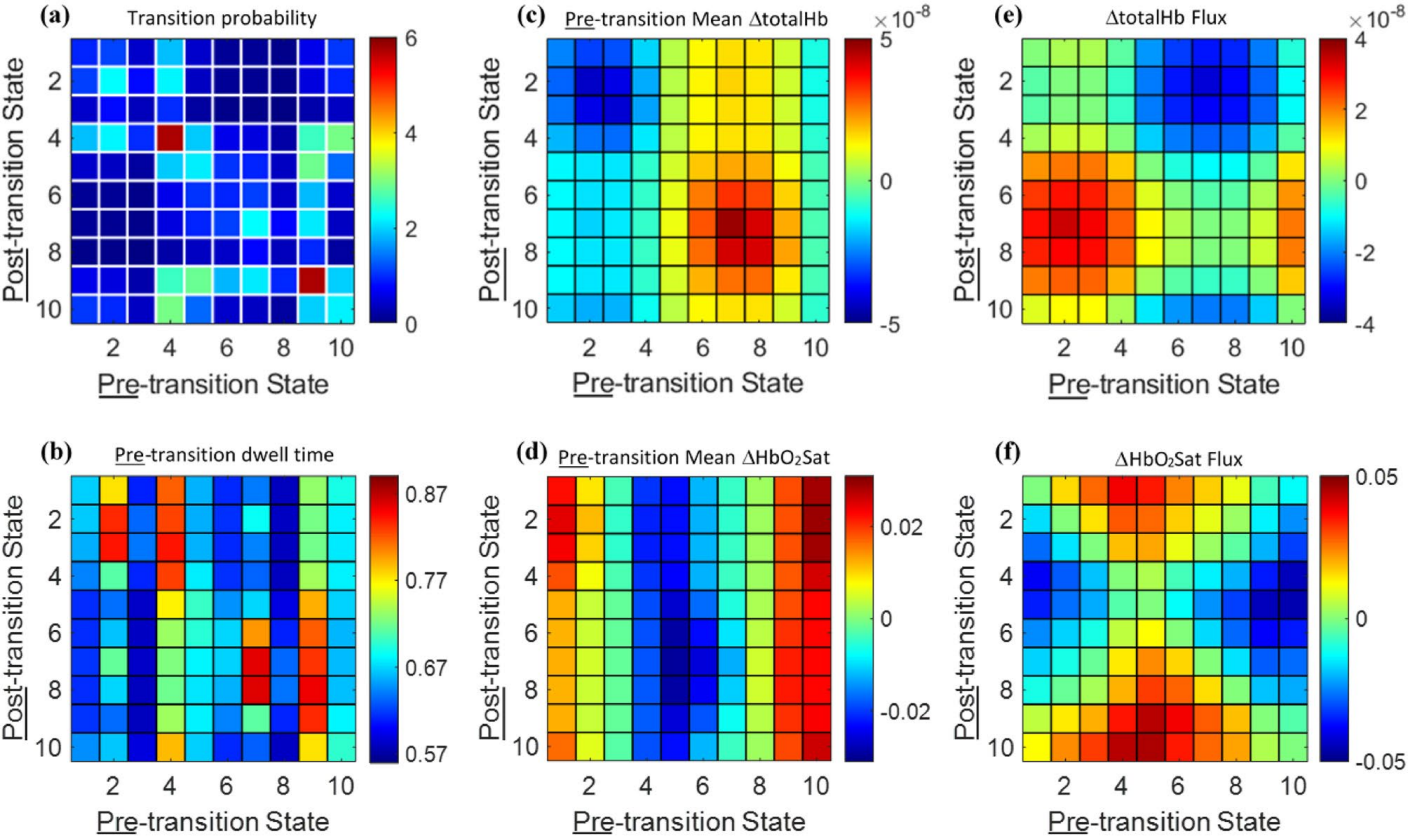
Transition-type patterns of individual network coefficients. Group-mean 10 × 10 adjacency matrices for non-cancer subjects (*n* = 45). In each panel, the plotted data is the average of left- and right-breast adjacency-matrix values. (**a**) transition probability (units are %). (**b**) pre-transition dwell time (seconds). (**c**–**d**) ΔtotalHb (M) and ΔHbO_2_Sat (%) pre-transition mean values. (**e**–**f**) ΔtotalHb (M) and ΔHbO_2_Sat (%) transition fluxes.

Shown in Fig. 2 are selected group-mean adjacency matrices computed using the fixed-reference ordinal partition network (fr-OPN) approach described in Methods, applied to control-subject data. Plotted are results for State-based (Hb-State transition probability in Fig. 2a, pre-transition dwell times in Fig. 2b) and Hb component-based measures (pre-transition mean values and fluxes for ΔtotalHb (Figs. 2c,e), and for ΔHbO_2_Sat (Fig. 2d,f)). Evident are varied contrasts compared to the absence of features in the cloud plots. Quantitative comparison of these features reveals correlations ranging from near zero (pre-transition dwell time vs. ΔHbO_2_Sat pre-transition mean) to a maximum of -0.68 (ΔHbO_2_Sat pre-transition mean vs. ΔHbO_2_Sat flux). The dissimilar patterns seen imply that varied information intrinsically exists in the measured time series but cannot be directly perceived from inspection of the primary data (Fig. 1b,c). Shown in Supplementary Figure 1 (Supplementary Note 2) is evidence that accompanying such varied patterns, are correspondingly dissimilar patterns of disease sensitivity. For the four network coefficients considered in Supplementary Figure 1, inter-breast comparisons yield *p* < 0.01 (unequal-variance *t* test) for 38–84% of transition types for breast-cancer subjects, but only 0–12% for non-cancer subjects.

Whereas these results reveal different behaviors within the steady-state measures, possible systematic trends among two or more classes of matrices cannot be appreciated by inspection. Phenomenological reviews of such trends can be an efficient strategy for recognizing key drivers of system behaviors, which are often obscured in the case of complex systems. To accomplish this, we have systematically explored co-dependent behaviors by plotting the quantity in one adjacency matrix against that in another. In the case of paired comparisons, this produced a total of 153 plots, with additional measures involving three or more adjacency matrices also available. The binary plots explored all possible pairings among the 18 classes of measures: the three Hb-State (transition probability and pre-, post-transition dwell times), and 15 Hb-component (flux and pre-, post-transition mean amplitude for each of the five Hb-signal components) adjacency matrices. Examination of these co-dependency findings constitute the remainder of this report.

### Evidence of structured co-dependent behaviors

If the transition-type patterns of the various adjacency matrices were as independent as inspection of Fig. 2 and Supplementary Figure 1 suggests, plots of one network coefficient against another would be expected to yield an unstructured distribution of points. While a minority of the plots generated do have this character (14%), most exhibit mainly smoothly varying trends including, unexpectedly, instances of fits to well-defined mathematical forms typical of saturable processes common in biology (e.g., enzyme catalyzed reactions). Because these findings represent a first report, where appropriate we have supplemented these observations with findings from modeling studies and noise measures, to document that trends seen have a biological origin and are not a consequence of applied mathematical transforms.

**Figure 3:**
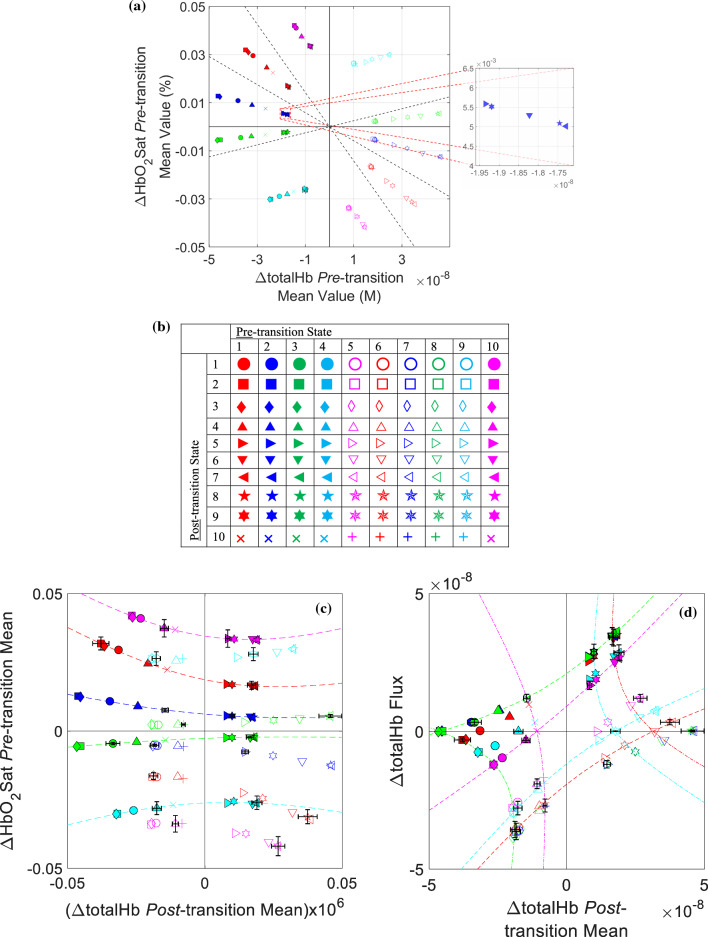
Exemplary highly structured co-dependence plots for Hb-component amplitudes. (**a**) Plot of ΔHbO_2_Sat pre-transition mean values versus ΔtotalHb pre-transition mean values. Group-mean data values for the tumor-bearing breast are shown (N = 18). Dashed lines are the secondary Hb-component null curves identified in Fig. 1a. Inset shows an expanded view of points that otherwise appear coincident. (**b**) Plot-marker color/fill/shape key, indicating the unique set of features assigned to each transition type. (**c**) Mean pre-transition ΔHbO_2_Sat versus mean post-transition ΔtotalHb (to improve the numerical stability of the curve-fitting algorithm, values on the abscissa are multiplied by a constant factor of 10^6^). (**d**) ΔtotalHb flux versus mean post-transition ΔtotalHb. In (**c**–**d**), coordinates of plotted points are T-breast group-mean values and dashed-line curves are hyperbolas that give the best-fitting approximations to the sets of same-color symbols (i.e., fixed pre-transition State). Dash-dot curves in (**d**) are hyperbolas that give the best-fitting approximations to the sets of same-shape symbols (i.e., fixed post-transition State). The curve-fitting function used^8^ implements the generalized eigenvector fit algorithm of Ref.^9^. This method returns the coefficients for the best-fitting conic section: *Ax*^2^ + *Bxy* + *Cy*^2^ + *Dx* + *Ey* + *F* = 0. The conclusion that all fits were hyperbolic follows from the empirical fact that in every case considered the value of the discriminant (i.e., *B*^2^ – 4*AC*) was positive^10^.

The ΔHbO_2_Sat versus ΔtotalHb plot in Fig. 3a (described more fully in Supplementary Note 3) is exemplary of a class of structured behaviors seen when the pre-transition mean amplitude for one Hb component is plotted against the pre-transition mean amplitude for another, for all 100 transition types. This figure reveals relationships that are not discernible by inspection of the same two coefficients when they are plotted separately (Fig. 2c,d). With 5 Hb-components, 10 paired plots can be generated, and the overall trends seen in Fig. 3a are obtained for all component pairings. To uniquely label each transition type, we applied different colors and symbol shapes and have further used open and filled symbols, as defined in Fig. 3b. The points in a given sector identify the paired pre-transition mean Hb-component amplitudes for the ten transition types that have a common pre-transition State. A similar plot of post-transition mean amplitudes for the same two Hb components would look almost the same as Fig. 3a, except that points in a given sector would have the same shape rather than the same color/fill (Supplementary Figure 2, Supplementary Note 4).

It is instructive to consider whether aspects of the Fig. 3a predominantly linear patterning and observed sequence of points within a sector follow inevitably from the way that States are defined here and in Refs.^6,7^. Our conclusion that they do not is established in Supplementary Note 5. The question was examined using the same model as adopted for the analysis of the State sequence, which ignores the radial dependence of data values (Fig. 1), in favor of an assumed uniform distribution. We computed the pre-transition mean-amplitude coordinates for all 100 transition types, under the null hypothesis that transitions between two points in ΔHbO_2_Sat-ΔtotalHb space will occur less often as the distance between the points increases. The findings were that the computed “spokes” are not linear, and that, unlike the pattern observed for real data, the post-transition State sequence differs across the pre-transition States, thus supporting the premise that the principal features of Fig. 3a are consequences of biological processes and not of any geometric dependence from applied State definitions.

The finding that trends in Fig. 3a depend on features of ΔtotalHb that are not otherwise evident (Supplementary Notes 3 and 5) raises the question whether this component or others influence trends seen in the individual adjacency matrices. To explore this, we implemented a strategy that compares parameter values for pairs of transitions that differ with respect to all Hb-signal components simultaneously, so that a dependence of a given component-related parameter on any of them can be detected (Supplementary Notes 6 and 7). Applying this method, we find that, whereas the transition-dependent patterning of contrast features varies significantly between State-transition and Hb-component matrices (Fig. 2, Supplementary Figure 1), all have an underlying dependency on ΔtotalHb (Table 1, Supplementary Figure 3). While the magnitude of the bias varies, in every case where the sign of ΔtotalHb reverses (category ‘1’ in Table 1) the average amplitude is reduced compared to when it is not changed (category ‘0’). We further note that the magnitudes of these biases are greater than their counterparts for the other Hb-signal components (not shown) (*p* = 2.5 × 10^−10^–5.4 × 10^−3^ (unequal-variance *t* test)). While physiological understandings may make the noted determinations unsurprising, they show that drivers of feature behavior can be effectively hidden.

**Table 1:** Distribution of adjacency-matrix values between transitions with/out reversal of ΔtotalHb algebraic sign.

Network coefficient	Breast group	‘0’^a^ Mean	‘1’^a^ Mean	‘0’-versus- ‘1’ *p*-value^b^
Transition probability (%)	T	1.19	0.64	*5.4 × 10*^*−3*^
	N^c^	1.27	0.53	*5.7 × 10*^*−6*^
	T-versus-N *p* val^d^	0.18	*4.5 × 10*^*−4*^	N/A
Pre-transition Dwell time (seconds)	T	0.68	0.63	*3.4 × 10*^*−3*^
	N	0.71	0.65	*1.0 × 10*^*−3*^
	T-versus-N *p* val^d^	*1.6 × 10*^*−4*^	*3.3 × 10*^*−4*^	N/A
|ΔHbO_2_Sat| Mean value^e^ (%)	T	0.022	0.017	*1.1 × 10*^*−3*^
	N	0.015	0.011	*1.2 × 10*^*−4*^
	T-versus-N *p* val^d^	*< 10*^*−10*^	* < 10*^*−10*^	N/A
|ΔtotalHb| Mean value^e^ (M)	T	2.7 × 10^−8^	1.5 × 10^−8^	*2.5 × 10*^*−10*^
	N	2.1 × 10^−8^	1.1 × 10^−8^	*5.2 × 10*^*−9*^
	T-vs.-N *p* val^d^	* < 10*^*−10*^	* < 10*^*−10*^	N/A

Italics indicate statistical significance (*p* < 0.05).

^a^‘1’ (‘0’) denotes transition types for which the algebraic sign of ΔtotalHb does (does not) change.

^b^Unequal-variance unpaired *t*-test; *n*(‘0’) = 40 (adjacency-matrix main diagonal is excluded), *n*(‘1’) = 50.

^c^Breast group N is the union of the U, L, and R groups.

^d^Correlated-samples *t*-test; inter-group correlations range from 0.82 to 0.998.

^e^Input data are averages of the pre- and post-transition means.

Additional evidence of structured co-dependent behaviors can be gained from examination of plots of paired coefficient values before and after a transition has occurred. Shown in Fig. 3c is a plot of the same Hb components as in Fig. 3a, but here the post-transition mean value of ΔtotalHb is plotted on the x-axis instead of its pre-transition mean. Inspection reveals that this substitution transforms the linear dependence to one having a well-defined hyperbolic functional form, a finding consistent with an underlying saturable phenomenon common among enzyme-driven behaviors. An alternative pairing shown in Fig. 3d involving substitution of ΔtotalHb flux on the y-axis, also reveals hyperbolic dependences, in this case extending to distinct trends for each pre- or post-transition State. As a qualitative measure of robustness of the data-value distributions in Fig. 3c,d, selected data points are annotated with two-dimensional (2D) error bars showing the standard errors for each plot variable. Unpaired and paired *t* tests were used for quantitative evaluations, as described in Supplementary Note 9.A. For the Fig. 3c pattern, the separations between data-point distributions for all nearest-neighbor pairs of pre-transition States are highly significant in one or both of the ΔHbO_2_Sat (*p* = 1.2 × 10^−9^–9.3 × 10^−4^) or ΔtotalHb (*p* = 5.5 × 10^−5^–4.3 × 10^−4^) dimensions. For Fig. 3d, all but the (largely superimposable) States 2,3 nearest-neighbor pair (and its reciprocal 7,8) have highly significant separations for both the pre- (*p* = 1.4 × 10^−11^–0.0097) and post-transition (*p* = 3.7 × 10^−11^–0.0029) groupings.

Examination of all 25 Hb-component pairings of flux versus post-transition mean value (Supplementary Figure 4) reveals that, while all produce smoothly varying trends, hyperbolic dependences are more limited than the linear trends seen for measures of mean component amplitude (Fig. 3a), being restricted to pairings that include the ΔtotalHb post-transition mean (Supplementary Note 8). Examination of the 25 pairings of pre- versus post-transition mean value likewise shows that hyperbolic dependences occur when ΔtotalHb mean values are plotted on one or both axes (Supplementary Note 8). For all such cases, an abbreviated representation is available from determination of the coordinates of the associated vertices and foci (Supplementary Figure 5, Supplementary Note 9)^11^.

### Informative alternative groupings of transition types

More careful inspection of Fig. 3c,d reveals a pattern that suggests an alternative strategy for appreciating evidence of structured behavior. It is seen that all trends for a fixed pre- or post-transition State have a similar functional form, but each has a unique amplitude dependence (e.g., distance from the origin), thus identifying two distinct classes of behavior (i.e., a State-dependent amplitude within a State-independent functional form). Recognizing that a common dependence suggests sensitivity to a common underlying process, we explored an alternative classification of transition types into groups having a fixed dependence between pre- and post-transition States.

**Figure 4:**
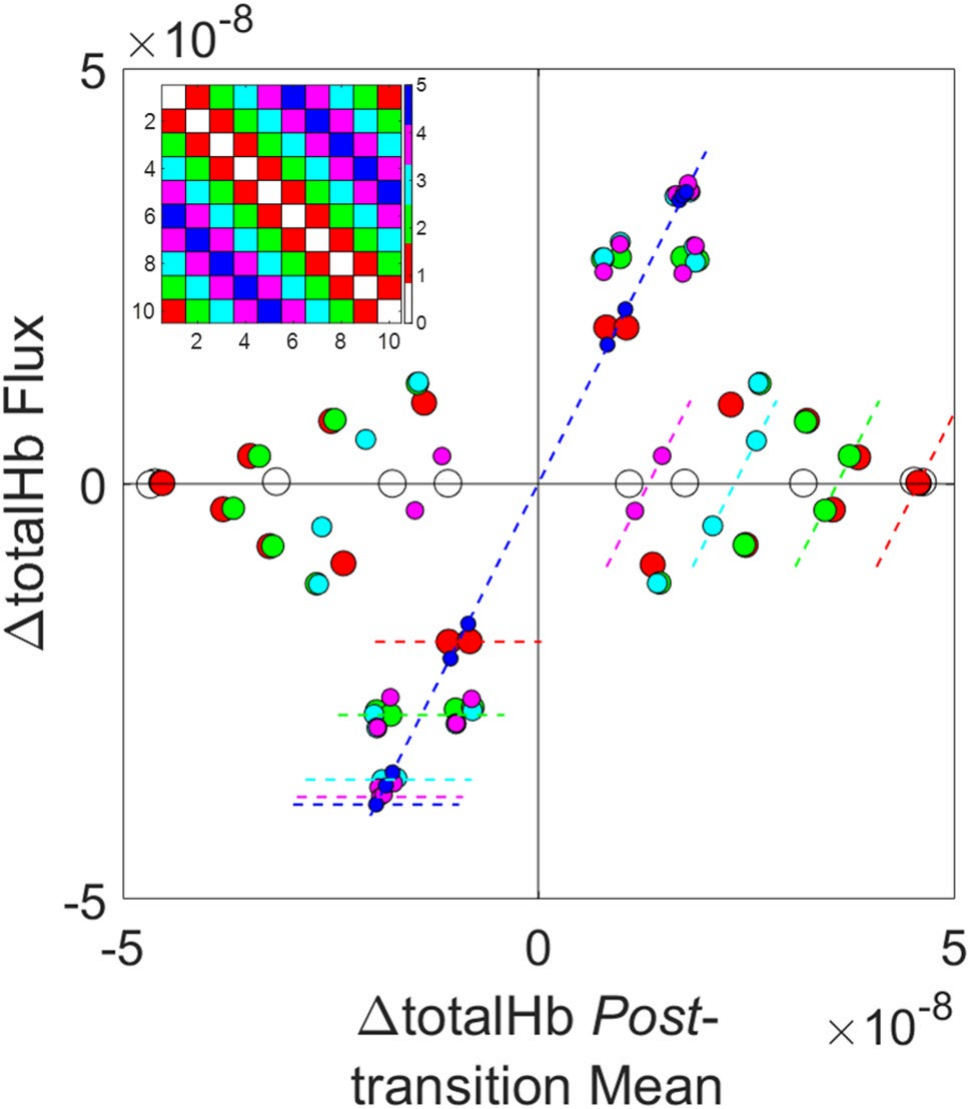
Transition-Class Trends Embedded in Binary Co-Dependences. Replot of the ΔtotalHb flux versus mean post-transition ΔtotalHb data in Fig. 3d, with points labeled according to the transition Class corresponding to each transition type (inset). Dashed line segments show the maximum values of ΔtotalHb flux (horizontal segments) and mean post-transition ΔtotalHb (oblique segments) for each Class, demonstrating an inverse relationship between the flux and mean-value coefficients.

A straightforward way to define such groupings is to select all transition types that lie a fixed distance above or below the adjacency matrices’ main diagonals (see inset of Fig. 4). These groupings are referred to as transition Classes. There are six Classes, numbered 0–5 in accordance with the distance, in State sectors, between the pre- and post-transition States. Each Class includes equal representations from all pre- and post-transition States, and all transition types in Class *m* (*m* = 0–5) have the property that exactly *m* Hb-signal components undergo changes of algebraic sign.

To demonstrate that transition Class information can provide additional insights into structured co-dependences, in Fig. 4 we have redrawn Fig. 3d with each marker relabeled according to its transition Class, revealing a systematic trend not evident by inspection of the original plot. It is seen that with increasing Class number, there are reciprocal trends in the ranges of values for the mean (decreasing) and flux amplitude (asymptotically approaches maximum value). The latter trend is present for all five Hb components (Supplementary Figure 6, Supplementary Note 10), demonstrating a binary response comprising a rapid rise phase (Classes 0–2) and an asymptotic phase (Classes 3–5). We conjectured that the bi-phasic trend reflects a transition from a larger or more heterogeneous set of unseen drivers affecting the Class 0–2 transition types to a smaller or more consistent set of influences on Classes 3–5. Accordingly, we anticipated that subsequent computations intended to reveal hidden-driver effects may yield notably different results for the 0–2 and 3–5 Class groupings.

### Transition class-dependent enzyme-like behaviors in Hb-signal component fluxes

**Figure 5:**
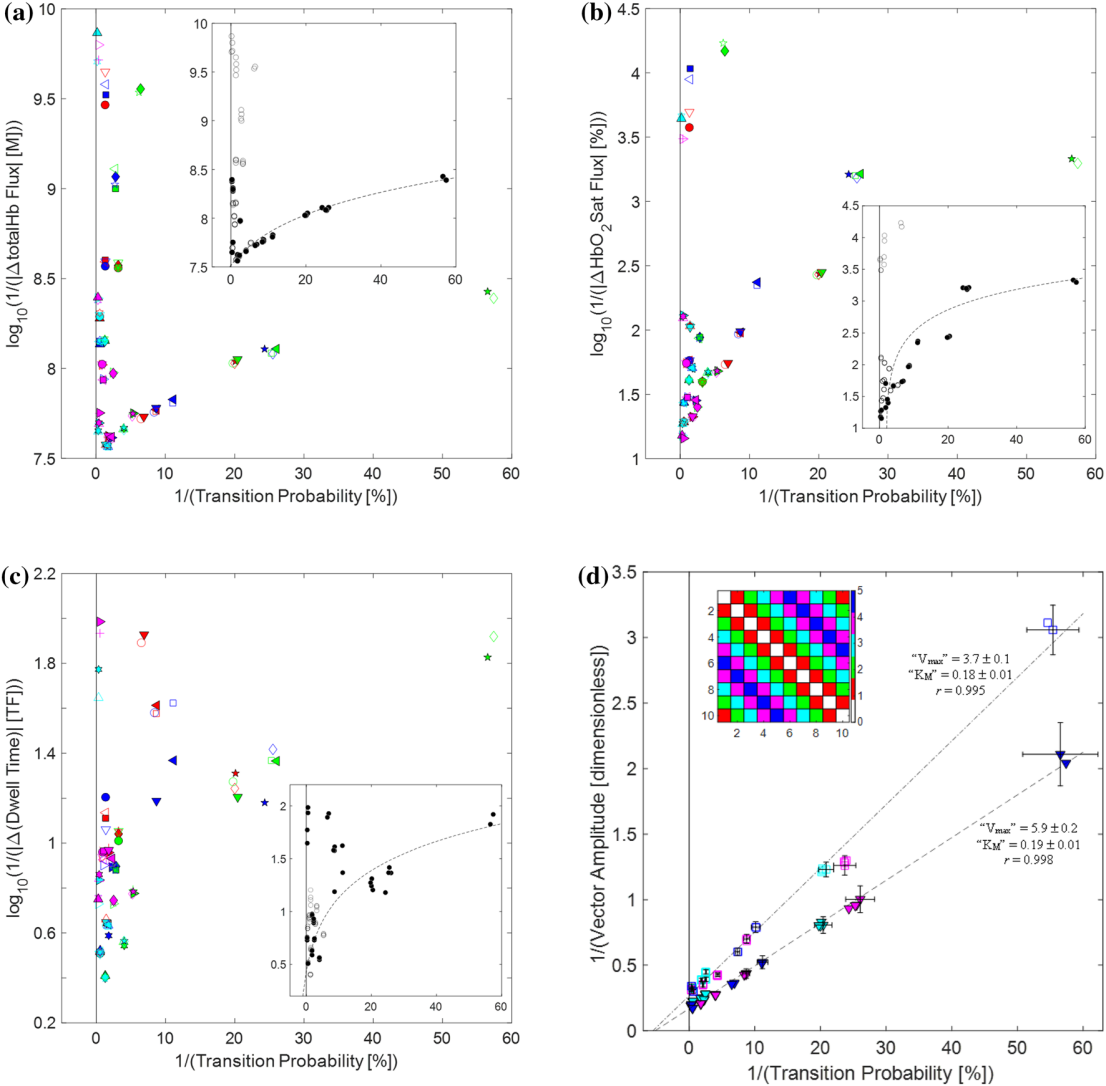
Enzyme-like behavior in composite network coefficient measures. Transition type-labeled plots, for T-breast group-mean data, of: (**a**) log_10_(1/|ΔtotalHb flux|) versus 1/(transition probability) [i.e., log_10_(1/|*ϕ*^ΔtotalHb^|) versus 1/*P*]; (**b**) log_10_(1/|*ϕ*^ΔHbO2Sat^|) versus 1/*P*; (**c**) log_10_(1/|Δ(dwell time)|) versus 1/*P*. Each panel’s inset is a replot of the same values as in the corresponding main plot, with filled and unfilled markers denoting transition types in Classes 3–5 and 0–2, respectively. Dashed-line curves are (log-transformed) linear fits to subsets of the filled points. In (**a**) (*n* = 38, *r* = 0.989), excluded are points in the 1/*P* < 2.5, log_10_(1/|*ϕ*^ΔtotalHb^|) > 7.64 region of the plot; in (**b**) the linear regression includes all filled points (*n* = 50, *r* = 0.90); in (**c**) (*n* = 40, *r* = 0.94), points in the 1/*P* < 15, log_10_(1/|Δ(dwell time)|) > 1.5 region of the plot are excluded. (**d**) Transition Class-labeled plot of 1/(vector amplitude) vs. 1/(transition probability) [i.e., 1/*A* vs. 1/*P*] for T- and L-breast group-mean data for Classes 3–5. The three network coefficients used as vector components (Eq. (11)) are ΔtotalHb flux, ΔHbO_2_Sat flux, and Δ(dwell time). For (**d**): inset 2D array shows the transition Class color coding; T- and L-breast data are plotted as filled triangles and unfilled squares, respectively; dashed (T) and dash-dot (L) lines are the linear fits to the full set (*n* = 50) of Classes 3–5 points.

The findings of hyperbolic relationships among coefficients of the Hb signal components can be initially considered as reflective of unseen enzyme-like actions. To further explore this along lines of classical enzyme activity plots, we invoked analogies between transition probability and a hypothesized canonical substrate, and between (absolute value of) transition flux and an associated enzymatic velocity (Supplementary Note 11), and generated plots of 1/|*ϕ*_*k*_^*X*^| (Eq. (10)) versus 1/*P*_*k*_ (Eq. (5)) for each Hb-signal component. Inspection reveals suggestions of (curvi) linear structure, but also notable deviations from the structured behavior. This is illustrated in Fig. 5a, for the case of *X* = ΔtotalHb and *ϕ*_*k*_^*X*^ computed using the SMTM averaging approach (Supplementary Note 12). A classical Lineweaver–Burk (L-B) analysis was performed that was limited to Classes 3–5 data values (black symbols in Fig. 5a inset). While the result is statistically significant (*r* = 0.38 (*p* = 0.018)), the dependence of 1/|*ϕ*_*k*_^*X*^| on 1/*P*_*k*_ is complex. Nevertheless, a large number of Classes 3–5 points plotted do show dependence consistent with a saturable process, with the deviations limited to a high transition-probability subset and nearly all elements of transition Classes 0–2. A second L-B computation, which considers only the non-deviating points (*n* = 38), yields a highly linear dependence (i.e., dashed curve in Fig. 5a inset; note that ordinate data values are log transformed), with *r* = 0.989 (*p* < 10^−10^). We tentatively concluded that an enzyme-like relationship is present, but it is only one of the factors that determine the overall of 1/|*ϕ*_*k*_^*X*^| versus 1/*P*_*k*_ dependence.

Examination of the full range of Hb components, averaging methods, and breast groups revealed that the subset of transition types that participate in the structured dependence is different, and seemingly arbitrary, for every component. This is illustrated in Fig. 5b,c, where Fig. 5b is a plot of 1/|*ϕ*_*k*_^*X*^| versus 1/*P*_*k*_ for *X* = ΔHbO_2_Sat (*r* = 0.90 (*p* < 10^−10^)), while in Fig. 5c the dependent variable is the reciprocal of (the absolute value of) the “dwell-time flux” defined as the difference between post- and pre-transition dwell-time values (Supplementary Note 13) (*r* = 0.94 (*p* < 10^−10^)). The composite of findings was taken as unsurprising, since flux values are expectedly influenced by multiple factors, but also as encouraging, in that the (assumed) enzymatic activity factor is sufficiently strong as to reveal itself in the plots at all.

Based on the parameter-sweep findings, we modified the double-reciprocal plotting procedure in a way that yielded improved evidence of structured behavior: flux values for each subject were transformed to a normalized quantity as described in Methods (Eq. (11)), which reduced the impact that inter-subject variability in flux amplitudes has on plotted group-mean values. Consequently, linear scales can be used for both axes, and there is a notable disease dependence. However, the Class 3–5 data points are still not aligned linearly and no non-arbitrary procedure is discernible for excluding the non-aligned transition types for any result based on a single Hb component.

The insight that enabled further development of the L-B analysis came from the recognition that the specific transition types that deviate from the structured behavior are different for each component considered in Fig. 5a,b,c. This raises the possibility that composite measures obtained by vectorially combining flux data for multiple components (in particular, the *t*′-scores, which are dimensionless, thereby avoiding incompatible-units concerns) may reveal a structured behavior that includes all Class 3–5 transition types. Shown in Fig. 5d is the result obtained when the mentioned refinements were applied to a 3-component flux measure (Eq. (11)) (Supplementary Note 13). Here we find an excellent linear fit over the entire span of coefficient values, demonstrating that off-trend values in each one-component fit are overcome by contributions from the other components. There are highly significant differences between both the Fig. 5d regression slopes (*p* = 1.6 × 10^−5^) and the y-intercepts (*p* < 10^−10^), and selected Fig. 5d data points are annotated with 2D error bars (± 1 standard error for each plot variable), as a qualitative measure of robustness of the linear trends. Similar-quality fits were obtained for a selected individual (slopes: *p* = 0.0026; intercepts: *p* = 1.1 × 10^−6^; Supplementary Figure 7), emphasizing that neither the qualitative aspects nor the goodness of fit require variance reduction from group averaging. Notably, the effect in tumor subjects is to enhance V_max_, which is consistent with the known action of NO, whose levels are increased in breast cancer^12^, on the enzyme soluble guanylyl cyclase^13^, a key driver of vascular smooth muscle contractility. We invoke this interpretation in recognition that vascular pulsatility is the dominant phenomenology affecting Hb signal dynamics.

### Evidence for tumor biomarker sensitivity in L-B plot coefficients

For the Fig. 5d demonstration of disease sensitivity, the plotted T-group vector amplitudes are averages over all breast-cancer subjects. We have extended the analysis to investigate whether the vector-computation results exhibit sensitivity to either the ER or Her2 breast-cancer biomarkers, by performing separate L-B analyses for the ER( +) and ER( −) subjects (*n* = 13 and 5, respectively), and for the Her2( +) and Her2( −) subjects (*n* = 9 and 9). Statistically significant differences between the regression slopes were found in both cases (ER: *p* = 2.2 × 10^−8^, Her2: *p* < 10^−10^), and between the ER( +) and ER( −) y-intercepts (*p* = 0.0081) (Supplementary Figure 8, Supplementary Note 14). Additionally, *t* tests were applied to vector amplitudes averaged across subjects (i.e., one net value per transition type) to compare the amplitudes for marker-( +) and marker-( −) subjects. Tests performed considered either all Classes 3–5 transition types, or only those for one of the four vector-component rankings identified in Table 2. Results demonstrate that vector amplitudes are sensitive to the presence or absence of a particular biomarker, and that the biomarker sensitivity differs across rank-order groupings (Table 2, Supplementary Note 14), thereby demonstrating that embedded within the resting-state time series are disease-sensitive discriminators that can aid in selection of a treatment approach.

**Table 2:** Impact of tumor marker status on tumor-breast flux-vector amplitude.

Tumor marker	Statistic	Considered transition types				
		All classes 3–5 (*n* = 50)	Rank-order 1 (*n* = 20)	Rank-order 2 (*n* = 10)	Rank-order 3 (*n* = 12)	Rank-order 4 (*n* = 8)
ER (*N*_(+)_ = 13, *N*_(−)_ = 5)	( +)-group mean [SD]	3.2 [1.4]	3.2 [0.8]	0.95 [0.26]	4.3 [0.7]	4.5 [0.3]
	( −)-group mean [SD]	3.9 [1.8]	3.7 [0.9]	1.1 [0.3]	5.5 [1.0]	5.6 [0.6]
	( +) vs. ( −) *t* test *p*-value	*0.030*	0.081	0.18	*0.0034*	*0.0015*
Her2 (*N*_(+)_ = 9, *N*_(−)_ = 9)	( +)-group mean [SD]	3.7 [1.6]	3.6 [0.9]	1.1 [0.3]	5.0 [0.9]	5.2 [0.5]
	( −)-group mean [SD]	3.1 [1.4]	3.0 [0.8]	0.87 [0.25]	4.2 [0.7]	4.4 [0.3]
	( +) v. ( −) test *p*-value	*0.044*	*0.031*	0.063	*0.026*	*0.0020*

Italics indicate statistical significance (*p* < 0.05).

The tabulated rank-order categories refer to the relative magnitudes of the three normalized quantities (i.e., *t*′-scores) used in computing vector amplitudes (Eq. (11)). Specifically, 1: **|***t*′(ΔtotalHb)**|** >|*t*′(ΔHbO_2_Sat)| >|*t*′(Δ(dwell time))|, 2: **|***t*′(ΔtotalHb)**|** >|*t*′(Δ(dwell time))| >|*t*′(ΔHbO_2_Sat)|, 3: **|***t*′(ΔHbO_2_Sat)**|** >|*t*′(∆totalHb)| >|*t*′(∆(dwell time))|, 4: **|***t*′(∆HbO_2_Sat)**|** >|*t*′(∆(dwell time))| >|*t*′(∆totalHb)|. (A colormap depicting the rank-order category, for each transition type in Classes 3–5 is shown in Supplementary Figure 14 (Supplementary Note 15)).

### Additional method validation studies

Recognizing that the methodology applied in this report has identified several novel parameter trends, we conducted a number of noise studies demonstrating that such trends are not an inevitable consequence of the applied mathematical manipulations (Supplementary Note 16).

## Discussion

The understanding that the varied organizational scales of biology and their homeostatic balance are strongly influenced by lifestyle, environmental factors and varied forms of molecular expression has motivated a focused buildout of resources to achieve a granular description of molecular expression and their responses to principal drivers^1,14,15^. This effort builds on the idea that descriptions of this type will support similarly individualized approaches to disease prevention, early detection, stratification and therapeutic interventions. Recognized as precision medicine, its implementation thus far is mainly built on gaining deep phenotypic descriptions of the compositional elements of biology.

Generally, not considered by this information mix are varied forms of physiological measures^4,5^. Reflecting the integration of a host of body functions, the relationship between macroscale behaviors recognized by such measures and molecular–scale descriptions and associated phenomenology are restricted principally to qualitative trends. As such, the information densities achievable from physiological measures are orders of magnitude lower than multi-omics measures.

It is our view that this dichotomy is not due to any fundamental information barrier, but rather speaks to current limits on feature recognition. The understanding that drivers of time-varying phenomenology are hidden to the sensing method motivates adoption of methods that can serve as useful surrogates. Among the varied approaches used to explore dynamic behaviors, attention is given to network methods with the understanding that useful descriptions of system behaviors can be appreciated without requiring detailed knowledge of the drivers of system dynamics. Experience shows that network formulations of physiological time-series measures (e.g., hemodynamic, bioelectric) is typically directed to assessing a network’s structure and stability^16^. While features of system organization are thereby revealed, network measures that explore system dynamics per se have been mainly absent. As recognized, the common approach of reducing information to a single metric (e.g., temporal correlation) strongly limits the granularity of accessible information compared to the rich details of the native signal^17^. Lost are dependences between the *fleeting* originating signals, whose short-term amplitudes vary owing to the feedback actions of a multitude of unseen factors.

Being data-driven, State descriptions are free to consider any of a host of observable phenomena. Here we have extended principal features of the hemoglobin signal (i.e., oxyHb, deoxyHb) to include other computable quantities (i.e., HbO_2_Sat, totalHb, and HbO_2_Exc). As the latter are dependent phenomena, it might be thought that they are simply redundant information. However, their inclusion provides a principled approach to objectively expanding the definable network space (Supplementary Note 1). Also, as recognized, the State definitions applied here are coarser than necessary^6^. Descriptions that are more restricted in the spatial and time domains are available, including the history dependence of State transitions. In the limit, network descriptions can be generated from even a single measured time series. Also available are descriptions that make use of information acquired from multiple forms of simultaneous measures. In terms of the hemoglobin signal, attention is drawn to the technique of diffuse correlation spectroscopy^18^, which provides measures of blood flow and has been shown sensitive to elements of the tumor phenotype^19^. Inclusion of these would extend the network to six components, rather than the five explored here. Similar considerations would also apply to other forms of time-varying physiological measures (see below).

Further recognized is the particular approach used to define network features. Here we assume that signals satisfying the same State definition are responses to similar levels of underlying drivers. It is our contention that this assumption is more strongly justified under the fixed-reference network-node definitions used in this report and in Ref.^6^ (fr-OPN; see “Methods”) than would be true if the time-varying multivariate OPN method^20^ had been employed instead (Supplementary Note 17). As outlined, the former supports more granular descriptions of network behaviors. Such noted flexibility of State descriptions, combined with other sensing measures, suggests that access to a broad range of small-scale behaviors might prove feasible.

As alternative network to time series methods are available^17^, it is useful to consider their expected utility for the aims of this study. Recognized are three principal strategies comprising use of either recurrence, visibility, or ordinal partition operations (RN, VN, and OPN respectively). Comparisons among them directs attention to how network nodes and edges are defined. For univariate measures, RN or VN methods are less desirable because network parameters are defined from sequences of temporal information, thus convolving temporal behaviors. Similarly, for RN, while multivariate methods can limit temporal convolution in node descriptions, a comparable impact on the variable recurrence times associated with network edges is lacking. Also, as mentioned, while more typical implementations of multivariate OPN methods can be used, these have the effect of blurring assignments of network transitions relative to those considered here (Supplementary Note 17). Overall, because the goal of these methods has been to appreciate topological features, it is unsurprising that their typical uses are not well suited for the aims of this study. It follows that appropriate exploration of other phenomenology (e.g., short-term dynamics, thermodynamics) can be expected to require careful curation of network properties.

It is likewise appreciated that well-established non-network time-series methods, while useful for classification^21^ or feature-extraction^22–24^, and potentially useful for pre-conditioning operations for the method of this report, do not generate data from which low-variance measures of short-term dynamics can be directly read. Additionally, while these methods lie on a spectrum with regard to the requisite degree of prior knowledge of the processes governing system dynamics (e.g., low for wavelets^21,22^, high for Kalman filtering and related methods^25,26^), mathematically they are more strongly assumption-dependent or model-based than the fr-OPN method used here.

Extension of the applied methodology to other forms of physiological monitoring also appears feasible. For the case of EEG or MEG, time-varying amplitudes of different frequency bands can serve as a mathematical counterpart to the hemodynamic components. The time series for each band would have a temporal mean value, with the instantaneous amplitude alternately above ( +) and below ( −) it, allowing for the definition of States and adjacency matrices in a manner directly parallel to that used for the fNIRS Hb-signal components. While adjacency-matrix computation could include temporal integration over the full measurement period as in this report, also plausible would be combining the time series of frequency-band States with those for EEG microstates computed as described in Ref.^27^. For example, the sub-intervals corresponding to only a specified microstate could be selected, and these contiguously arranged to create a modified time series. The State-network analysis applied to microstate-specific time series could help distinguish between intra-microstate and microstate-switching phenomenology.

For fMRI, counterparts to the EEG microstates can be computed from resting-state image time series^28^, but equivalency to the EEG frequency bands is mainly lacking due to lower sampling rates. As an alternative, a States definition can be based on the algorithmic feature that, for every image frame, a probability of each microstate being the “true” one is generated^28^. For their purposes, the authors took the reasonable approach of changing the largest probability value to 1 and all others to 0. To define counterparts of the Hb-signal components and States, we would propose instead to compute mean values for each microstate’s probability time series, and at every time frame note which microstates have probabilities above ( +) or below ( −) their temporal means. In this way, each microstate (which is a spatial distribution of BOLD-signal amplitudes) is regarded as a signal component, and the States as different combinations of probability-based algebraic signs. From the State and component-amplitude time series, network adjacency matrices would be computed in the same manner as reported here.

Returning to the goal of including physiological measures as indicators for precision medicine, our demonstration of specific coefficient patterning across the varied adjacency matrices (e.g., Fig. 2 and Ref.^6^), sensitivities to ER and Her2 expression, and the ability to recognize particular forms of structured behaviors (e.g., Fig. 5d), supports the hypothesis that the considered methodological extensions would likely identify associations to other forms of small-scale behaviors. In one form, recognition of these could look to parallel methods currently applied to explore multi-omics findings to identify correlations between feature classes and their dependence on varied disease characteristics^15^. For fNIRS measures, attention is drawn to inflammatory responses and factors impacting oxidative metabolism. Another would be to recognize dependencies between specific pharmacological manipulations and their impact on individual coefficient patterns or their co-dependencies. Then, because fNIRS-based network features and multi-omics information reflect different biological determinants and time scales, in combination it may be possible, for example, to improve on the reported accuracy of the PINSPlus-tumor subtyping algorithm^15,29^. While we have emphasized recognition of structured behaviors, more generally it seems likely that disease-sensitive coefficient patterns need not follow any particular mathematical form. In such cases, exploration of coefficient matrices using varied data classification schemes would be warranted.

In summary, we have shown that by invoking methodology that can reveal low-variance measures of short-term dynamics, and by exploring their higher-order co-dependencies, evidence of behaviors consistent with actions arising from molecular-cellular processes is revealed, even though the primary sensing method is macroscopic. We expect that this methodology can be extended to other forms of physiological monitoring. We further recognize that extension of State class descriptions and their expected history dependences will aid in identifying the actions of other forms of unseen drivers which, when combined with multi-omics methods, will extend the information base upon which decisions of precision medicine depend, while offering the advantage of on-demand monitoring.

## Methods

### Instrumentation and data collection

Optical time-series measures of the Hb signal were acquired as previously described^30^. Simultaneous bilateral resting-state measures of the breast were subsequently acquired at a rate of 1.8 image frames per second, for a period lasting ~ 5–10 min. Raw data were screened for evidence of signal-quality degradation resulting from signal attenuation by very large breasts, or by poor skin-optode contact for very small breasts, using methods described in Ref.^31^. To avoid introducing asymmetry into the bilateral signal, channels excluded from one breast were also excluded from the other. Under-sampling biases were avoided by including data only from subjects who had at least 60% of channels pass the data-quality checks.

### Preliminary data processing

3D-tomographic images were reconstructed by normalizing measured signal intensities of each data channel to their respective temporal mean values, and then solving a system of linear equations based on solutions to the diffusion equation^32,33^. The computed wavelength-dependent absorption coefficient values were transformed to yield spatial maps of concentration variations about their respective temporal mean values, for the independent (*i.e.*, oxygenated and deoxygenated) components of the Hb signal. A linear detrending operation (MATLAB ‘detrend’ function) was applied across the time dimension of the reconstructed ΔoxyHb and ΔdeoxyHb images, to remove any long-term drifts that may be present in the data, while retaining fluctuations on shorter time scales. Values of three dependent Hb components—total Hb, Hb oxygen exchange (HbO_2_Exc) and Hb oxygen saturation—were computed from the detrended ΔoxyHb and ΔdeoxyHb measures, via the formulas: ΔtotalHb = ΔdeoxyHb + ΔoxyHb, ΔHbO_2_Exc = ΔdeoxyHb—ΔoxyHb, and ΔHbO_2_Sat = 100⋅Δ(oxyHb/totalHb), yielding 5 classes of image time-series measures of the Hb signal that served as input to the methods described below.

### Fixed reference ordinal partition transition network (fr-OPTN) representation of the Hb signal

Shown in Fig. 1a is the coordinate system used to define an ordinal partitioning of the five time-evolving Hb signal components relative to their respective temporal mean values^6,34^. Derived is a ten-state system whose state assignments are determined by a particular combination of the algebraic signs of the signal components. Identified is a time-evolving Markov chain from which improved statistics of mean coefficient values can be obtained using expressions listed below. The ten States constitute the vertices or nodes of a network that is fully connected (i.e., every pair of nodes is joined by an edge), as transitions between all possible pairings of States are observed in every image time series. Any of the network coefficients described subsequently may be considered as network edge weights; thus the States and transition types are the vertices (*V*) and edges (*E*), respectively, of a graph (*G*), while each of the subsequently defined network features constitutes a different form of edge weight (*w*); formally, *G* = (*V*, *E*, *w*).

This network mapping methodology invokes a fixed reference comparison (temporal mean) in contrast to a previously described OPN procedure^20^, which employs a time-varying reference. As emphasized in Supplementary Note 17, this difference notably extends the granularity by which adjacency profiles can be considered, which, as shown in Results, provides access to previously unrecognized structured behaviors and to further evidence of physiologically relevant embedded features.

### Quantification of inter-state transition coefficients

Every reconstructed image time series has the form of a *N*_*t*_ × *N*_*v*_ matrix, where *N*_*t*_ and *N*_*v*_ are the numbers of measurement time frames and image voxels, respectively. In turn, each voxel occupies exactly one of the ten Hb States at every time frame. Using *i* and *j* for the time and space variables, respectively, *s*_*ij*_ denotes the State of the *j*-th voxel in the *i*-th time frame, and is the *ij*-th element of the *N*_*t*_ × *N*_*v*_ matrix **S**. The method applied in this report for counting transitions differs from that of Ref.^6^, in that here we compare the Hb State in each time frame to the one immediately following it. A consequence of this “synchronous” definition is that here even the case where a voxel is in the same State at successive times frames is counted as a transition, with the result that 100 transition types are defined, in contrast to 90 in Ref.^6^; in other respects, the change in transition-counting method does not have a substantial impact on results obtained previously. However, computation of the transition matrix **T** becomes more straightforward than under the previously described “asynchronous” definition: here, **T** is a linear combination of two time-shifted copies of **S**:

1 $${\mathbf{T}}_{{1:N_{t} - 1,1:N_{v} }} = 10 \cdot \left( {{\mathbf{S}}_{{1:N_{t} - 1,1:N_{v} }} - 1} \right) + {\mathbf{S}}_{{2:N_{t} ,1:N_{v} }} .$$

Thus, all values in the ranges of 1–10, 11–20, …, 91–100 correspond to transitions from State 1, 2, …, 10 (the nomenclature we adopt for this report is that this is the pre-transition State), while all values ending in 1, 2, …, 9, 0 correspond to transitions into State 1, 2, …, 9, 10 (the post-transition State). We also note that the dimensions of **T** are (*N*_*t*_–1) × *N*_*v*_, and that the interval between the *i*-th and (*i* + 1) -th time *frames* constitutes the *i*-th time *step*.

#### Computation of inter-state transition probabilities

The absolute transition count for a given subject and the *k*-th transition type, *k* = 1, …, 100, is obtained by first generating the matrix **U**_*k*_, whose *ij*-th element is:

2 $$\left( {U_{k} } \right)_{ij} = \left\{ {\begin{array}{*{20}c} {1,\quad T_{ij} = k} \\ {0,\quad T_{ij} \ne k} \\ \end{array} } \right.\quad .$$

Summing **U**_*k*_ over its spatial dimension and transposing yields **C**_*k*_, the 1 × (*N*_*t*_–1) matrix of type-*k* transition counts, whose *i*-th element is:

3 $$C_{ki} = \sum\limits_{j = 1}^{{N_{v} }} {\left( {U_{k} } \right)_{ij} } .$$

Performing the computation in Eq. (3) for all 100 transition types produces **C**, a 100 × (*N*_*t*_–1) matrix of transition counts, where **C**_*k*_ is the *k*-th row of **C**.

As an illustration of method, we show an example of a **C** matrix in Supplementary Figure 9. While the qualitative properties of the plotted **C** matrix (*e.g.*, alternately high and low counts), are typical, the durations and starting times of the transition-count bands are subject-specific. Thus the information in **C** is not immediately amenable to inter-subject comparison and quantitative analysis. We convert it to a more tractable form by, first, summing over the temporal dimension to produce the 100-element transition-count vector **c**, whose *k*-th element is:

4 $$c_{k} = \sum\limits_{i = 1}^{{N_{TS} }} {C_{ki} } \;,$$

and *N*_*TS*_ = *N*_*t*_ – 1 is the number of measurement time steps. Each *c*_*k*_ value is directly proportional to the measurement duration, which ranges from 400 to 1000 time frames for different study participants (median value and mode = 700, and disease and non-disease subject groups are measurement duration-matched (*p* = 0.14)). Accordingly, for inter-subject comparisons we normalize **c** to the sum of all values in **c**:

5 $${\mathbf{P}} = 100\frac{{\mathbf{c}}}{{\sum\limits_{k = 1}^{100} {c_{k} } }}\;.$$

The *k*-th element of (dimensionless) **P** is the probability for the *k*-th transition type, in units of percent.

It should be noted that the summation order in the preceding description—first across image voxels (Eq. (3)), then time (Eq. (4))—could be reversed, or the **U**_*k*_ matrix (Eq. (2)) could be summed across both dimensions simultaneously, without impact on the computed values of transition probability (Eq. (5)). In contrast, computed values of the network parameters defined in the remainder of this section are dependent on the sequence of operations. Simultaneous summation across both dimensions—the “grand average” (GA)—is the method that is most straightforward, is presented in subsequent equations, and was used to generate all Results in this report except where indicated otherwise. A description of the alternative data-averaging schemes—“temporal mean of the spatial mean” (TMSM), “spatial mean of the temporal mean (SMTM)—and a consideration of circumstances in which their use may provide useful counterpoints to GA, is presented in Supplementary Note 12.

#### Computation of inter-transition dwell times

There are ten transition types that correspond to a voxel being in the same Hb State in successive time frames (using the single-number indexing defined in Eq. (1), they are types 11*n* – 10, where *n* = 1–10). The term adopted here to describe occurrences of these transition types is that the voxel “dwells in” State *n* during that time step. For each transition type *k*, two distinct average dwell times are extracted from transition matrix **T**. These are the mean number of time frames that voxels dwell in the pre-transition state ($s_{1} = \left\lceil {k/10} \right\rceil$, where $\left\lceil x \right\rceil$ (i.e., the ceiling function) is the smallest integer ≥ *x*) prior to transition *k*, and the mean number of frames that they dwell in the post-transition state (*s*_2_ = *k* – 10(*s*_1_ – 1)) following it. We introduce the quantities $\left( {n_{{s_{1} }}^{k} } \right)_{ij}$ and $\left( {n_{k}^{{s_{2} }} } \right)_{ij}$, to denote the pre- and post-transition dwell times, respectively, for the *i*-th time-step transition in the *j*-th voxel. That is, $n_{{s_{1} }}^{k}$ is the number of time frames dwelt in State *s*_1_ prior to a type-*k* (i.e., from *s*_1_ to *s*_2_) transition, and $n_{k}^{{s_{2} }}$ is the number of time frames dwelt in State *s*_2_ following a type-*k* transition. We further introduce *τ*_*k*_^(1)^ and *τ*_*k*_^(2)^ to denote the mean pre- and post-transition dwell times, respectively, for type-*k* transitions. Thus, we have:

6 $$\tau_{k}^{\left( 1 \right)} = \frac{1}{{c_{k} }}\sum\limits_{j = 1}^{{N_{v} }} {\sum\limits_{i = 2}^{{N_{TS} }} {\left( {U_{k} } \right)_{ij} \left( {n_{{s_{1} }}^{k} } \right)_{ij} } } ,\quad \tau_{k}^{\left( 2 \right)} = \frac{1}{{c_{k} }}\sum\limits_{j = 1}^{{N_{v} }} {\sum\limits_{i = 1}^{{N_{TS} - 1}} {\left( {U_{k} } \right)_{ij} \left( {n_{k}^{{s_{2} }} } \right)_{ij} } } ,$$

where (*U*_*k*_)_*ij*_ (Eq. (2)) is 1(0) if a type-*k* transition does (does not) take place in the *j*-th voxel in the *i*-th time step, and *c*_*k*_ (Eq. (4)) is the total number of type-*k* transitions in the image time series. The two dwell times have different time-step summation limits in the index-*i* summations of Eq. (6), because pre (post)-transition dwell time cannot be evaluated for transitions that occur during the first (last) time step.

For the ten *s*_*n*_ → *s*_*n*_ transition types there can be two or more consecutive occurrences of the same type, and this would produce multiple values of $n_{{s_{1} }}^{k}$ and $n_{k}^{{s_{2} }}$ for a single dwell period. In these instances, only the largest values of $n_{{s_{1} }}^{k}$ and $n_{k}^{{s_{2} }}$ in each sequence of consecutive occurrences are included in the Eq. (6) summations.

#### Quantification of transition-linked hemoglobin-concentration and -saturation changes

The Hb States and transitions are defined in a manner that considers a fixed-reference ordinal dependence (i.e., independent of component amplitudes, and hence of distance from the axes or origin in Fig. 1). At the same time, image time series contain information on the magnitude of each Hb-signal component, which can be combined with the categorical information to compute the average levels of the five components of the Hb signal before and after each type of transition, as well as the average amounts by which those levels change during transitions.

The starting point for the concentration- and saturation-dependent quantities is five matrices, one for each component of the Hb signal, defined in a similar manner to the **T** matrix in Eq. (1):

7 $$\begin{gathered} \Delta^{2} {\mathbf{D}}_{{1:N_{t} - 1,1:N_{v} }} = \Delta {\mathbf{D}}_{{2:N_{t} ,1:N_{v} }} - \Delta {\mathbf{D}}_{{1:N_{t} - 1,1:N_{v} }} , \\ \Delta^{2} {\mathbf{E}}_{{1:N_{t} - 1,1:N_{v} }} = \Delta {\mathbf{E}}_{{2:N_{t} ,1:N_{v} }} - \Delta {\mathbf{E}}_{{1:N_{t} - 1,1:N_{v} }} , \\ \Delta^{2} {\mathbf{O}}_{{1:N_{t} - 1,1:N_{v} }} = \Delta {\mathbf{O}}_{{2:N_{t} ,1:N_{v} }} - \Delta {\mathbf{O}}_{{1:N_{t} - 1,1:N_{v} }} , \\ \Delta^{2} {\mathbf{S}}_{{1:N_{t} - 1,1:N_{v} }} = \Delta {\mathbf{S}}_{{2:N_{t} ,1:N_{v} }} - \Delta {\mathbf{S}}_{{1:N_{t} - 1,1:N_{v} }} , \\ \Delta^{2} {\mathbf{To}}_{{1:N_{t} - 1,1:N_{v} }} = \Delta {\mathbf{To}}_{{2:N_{t} ,1:N_{v} }} - \Delta {\mathbf{To}}_{{1:N_{t} - 1,1:N_{v} }} , \\ \end{gathered}$$

where Δ**D**, Δ**E**, Δ**O**, Δ**S** and Δ**To** denote the image time series (formatted as *N*_*t*_ × *N*_*v*_ matrices) of ΔdeoxyHb, ΔHbO_2_Exc, ΔoxyHb, ΔHbO_2_Sat and ΔtotalHb, respectively, while Δ^2^**D**, Δ^2^**E**, Δ^2^**O**, Δ^2^**S** and Δ^2^**To** are the corresponding concentration and saturation changes that attend each transition.

Proceeding in parallel with the transition probability derivation, the analogue of Eq. (2) is:

8 $$\left( {U_{k}^{X} } \right)_{ij}^{\left( 1 \right)} = \left\{ {\begin{array}{*{20}r} \hfill {\Delta X_{ij} ,\quad T_{ij} = k} \\ \hfill {0,\quad T_{ij} \ne k} \\ \end{array} } \right.,\quad \left( {U_{k}^{X} } \right)_{ij}^{\left( 2 \right)} = \left\{ {\begin{array}{*{20}r} \hfill {\Delta X_{{\left( {i + 1} \right)j}} ,\quad T_{{\left( {i + 1} \right)j}} = k} \\ \hfill {0,\quad T_{{\left( {i + 1} \right)j}} \ne k} \\ \end{array} } \right.,\quad \left( {U_{k}^{X} } \right)_{ij}^{\left( 3 \right)} = \left\{ {\begin{array}{*{20}r} \hfill {\Delta^{2} X_{ij} ,\quad T_{ij} = k} \\ \hfill {0,\quad T_{ij} \ne k} \\ \end{array} } \right.\;.$$

Thus, the arrays with superscripts ‘(1)’, ‘(2)’ and ‘(3)’ contain pre-transition, post-transition, and transition-associated change data for the *k*-th transition type, respectively. We sum the arrays defined in Eq. (8) over position and time, and account for inter-subject variations in measurement duration by normalizing to the transition count:

9 $$\left( {\mu_{k}^{X} } \right)^{\left( 1 \right)} = \frac{1}{{c_{k} }}\sum\limits_{j = 1}^{{N_{v} }} {\sum\limits_{i = 1}^{{N_{TS} }} {\left( {U_{k}^{X} } \right)_{ij}^{\left( 1 \right)} } } ,\quad \left( {\mu_{k}^{X} } \right)^{\left( 2 \right)} = \frac{1}{{c_{k} }}\sum\limits_{j = 1}^{{N_{v} }} {\sum\limits_{i = 1}^{{N_{TS} }} {\left( {U_{k}^{X} } \right)_{ij}^{\left( 2 \right)} } } ,$$

and

10 $$\phi_{k}^{X} = \frac{1}{{c_{k} }}\sum\limits_{j = 1}^{{N_{v} }} {\sum\limits_{i = 1}^{{N_{TS} }} {\left( {U_{k}^{X} } \right)_{ij}^{\left( 3 \right)} } } .$$

The (*μ*_*k*_^*X*^)^(1)^ parameter is the mean value, per transition, of pre-transition concentration or saturation, while (*μ*_*k*_^*X*^)^(2)^ is the corresponding post-transition value. We refer to *ϕ*_*k*_^*X*^ as the flux for signal component *X* and transition type *k*. (A related quantity, called the transition mass in Ref.^6^, is computed by substituting the product of *N*_*TS*_ and *N*_*v*_ for *c*_*k*_ in Eq. (10) but is not considered in this report.)

### Computation of higher-order adjacency matrix co-dependent values

If a selected number *M* of State- or transition-derived parameters are treated as coordinate axes of a mathematical space of *M* dimensions, then the parameter values for the *k*-th transition type can be plotted as a single *M*-dimensional point in that space. For most of the results presented in this report, *M* = 2 and the parameter values are plotted in their original, dimensional units (e.g., seconds for dwell times, molar (concentration) for ΔtotalHb pre-transition mean values). However, also explored is a variation on the same idea, in which the *k*-th-transition-type parameter values are converted to dimensionless units; this additional, normalization step allows us to compute an amplitude (and direction, if desired) for the resulting *M*-dimensional vector.

A particular example considered in this report is the vectors obtained for the three-parameter combination of ΔtotalHb transition flux, ΔHbO_2_Sat transition flux, and transition dwell time. A vector amplitude cannot be directly computed as *A* = [(ΔtotalHb flux)^2^ + (ΔHbO_2_Sat flux)^2^ + (Δ(dwell time))^2^]^1/2^ (where Δ(dwell time) = (post-transition dwell time) – (pre-transition dwell time)), because the quantities considered have different units. To eliminate unit inconsistency, each parameter was converted to dimensionless *t*′-scores: *t*_*k*_′ = (*x*_*k*_ – *m*)/*s*, where *x*_*k*_ = parameter value for the *k*-th transition type, *m* = mean value across all 100 transition types, *s* = standard deviation across all types. However, in order to preserve inter-breast disparities that may be present in the original data^6^, the data values for each breast were referenced to the mean and standard deviation for the contralateral breast (e.g., *t*′(T) = (*x*(T) – *m*(U))/*s*(U), where T, U = tumor-bearing and contralateral unaffected breast, respectively, of subjects who have unilateral breast cancer; *t*′(L) = (*x*(L) – *m*(R))/*s*(R), where L, R = left and right unaffected breast, respectively, of non-cancer subjects). Vector amplitudes for the *k*-th transition type and breast group *X* (where *X* is one of L, R, T or U) were subsequently computed as:

11 $$A_{k} \left( X \right) = \sqrt {t_{k}^{\prime } \left( X \right)_{\Delta totalHb}^{2} + t_{k}^{\prime } \left( X \right)_{{\Delta HbO_{2} Sat}}^{2} + t_{k}^{\prime } \left( X \right)_{{\Delta \left( {dwell \, time} \right)}}^{2} } .$$

### Study participants

Findings reported herein were derived from publicly accessible, de-identified data from 63 subjects—18 with confirmed breast cancer (6 right-breast, 12 left-breast, average tumor size ~ 2.7 cm, range 0.5–6 cm), and 45 age- and BMI-matched control subjects, 23 of whom had evidence of non-malignant pathologies in one or both breasts^35^. A more detailed description of enrolled subjects is given in Ref.^30^ and in Supplementary Table S1.

### Supplementary Information

Supplementary Information.

## Data Availability

The volumetric image time series data used are available on the Open Science Framework: “Resting-State Simultaneous Dual-Breast Imaging.” OSF Resting-State Simultaneous Dual-Breast Imaging (https://osf.io/4cr3z/).
