# Supplementary material for: Hemoglobin signal network mapping reveals novel indicators for precision medicine
Source: Sci Rep. 2023 Oct 25;13:18257. doi: 10.1038/s41598-023-43694-7 (PMC10600136; doi:10.1038/s41598-023-43694-7)
Supplement: Supplementary file 1 — Supplementary Information. [file 41598_2023_43694_MOESM1_ESM.pdf]

Hemoglobin Signal Network Mapping Reveals Novel Indicators for Precision Medicine

Randall L. Barbour[1] and Harry L. Graber[1,2]

[1]SUNY Downstate Health Sciences University, Department of Pathology, 450 Clarkson Avenue, Brooklyn, NY 11203

[2]Photon Migration Technologies Corp., 15 Cherry Lane, Glen Head, NY 11545

Supplementary Material

Supplementary Notes

1. Functional form of secondary axes / null lines in five-component coordinate systems.

As shown throughout this report, the availability of Hb-State sector boundaries is instrumental for interpretation of findings such as those of Fig. 3a and Supplementary Fig. 2. Here we describe the mathematical procedure used to generate the null curves for the non-orthogonal signal components (i.e., ΔdeoxyHb, ΔHbO_2Exc and ΔoxyHb) in the ΔHbO_2Sat-vs.-ΔtotalHb coordinate system of Fig. 1a. It is also shown that in coordinate systems having ΔHbO_2Sat plotted on the *x*- or *y*-axis, some of the null curves are nonlinear.

As explained in [1], mathematical expressions for spatiotemporal changes in HbO_2Sat must take into account the absolute levels of oxyHb and totalHb. Here we use the symbols O_0, S_0 and T_0 to denote the baseline mean values of oxyHb, HbO_2Sat and totalHb, respectively, and O, S and T for the respective instantaneous values of those same components. It follows that $O = O_0 + \Delta O$, $S = S_0 + \Delta S$ and $T = T_0 + \Delta T$, where ΔO, ΔS and ΔT are ΔoxyHb, ΔHbO_2Sat and ΔtotalHb, respectively.

The definition of HbO_2Sat in terms of oxyHb and totalHb gives us:

$$S_0 = 100\frac{O_0}{T_0}, \quad S = S_0 + \Delta S = 100\frac{O}{T} = 100\frac{O_0 + \Delta O}{T_0 + \Delta T},$$

from which we can derive a general expression, valid at all points in the ΔHbO_2Sat vs. ΔtotalHb coordinate system (Fig. 1a), for ΔS in terms of ΔO and ΔT:

$$\begin{aligned}\Delta S &= 100\frac{O_0+\Delta O}{T_0+\Delta T}-S_0 \\ &= 100\left(\frac{O_0+\Delta O}{T_0+\Delta T}-\frac{O_0}{T_0}\right)=100\frac{T_0\cdot\left(O_0+\Delta O\right)-O_0\cdot\left(T_0+\Delta T\right)}{T_0\cdot\left(T_0+\Delta T\right)} \\ &= 100\frac{T_0\Delta O-O_0\Delta T}{T_0\cdot\left(T_0+\Delta T\right)}.\end{aligned} \tag{S1.1}$$

While it was not necessary to explicitly consider the other two Hb components in the preceding derivation, they will appear in subsequent ones. We therefore introduce the symbols ΔD and ΔE to denote ΔdeoxyHb and ΔHbO_2Exc, respectively, and D_0 for the baseline mean value of deoxyHb.

Mathematically, each null curve in Fig. 1a is a constraint on the general formula in Eq. (S1.1). For example, movement along the ΔdeoxyHb (ΔoxyHb) null curve in the direction of increasing ΔtotalHb means that the concentration of totalHb is rising while the deoxyHb (oxyHb) concentration is held constant. The addition of pure oxyHb (deoxyHb) is required in order to satisfy the constraint, and the value of HbO_2Sat consequently rises (falls). Similarly, raising totalHb while holding HbO_2Exc fixed (i.e., moving along the ΔHbO_2Exc null curve in the direction of increasing ΔtotalHb) requires the addition of oxyHb and deoxyHb in equal amounts; since the baseline HbO_2Sat value is >50%, HbO_2Sat falls (approaching 50% as its limiting value) as totalHb increases. Subsequently we will show that the constraint associated with each Hb component allows us to derive the corresponding null-curve formula from Eq. (S1.1). As a final preliminary point, we note that there are biological limits on possible deoxyHb and oxyHb concentrations, but we do not take these as mathematical constraints. In the following we demand only that D, O and T be non-negative, and that S lie in the range of 0-100%.

ΔdeoxyHb = 0:

Here the constraint is $\Delta D = 0$, from which it follows that $\Delta T = \Delta O$. Then we can replace ΔO with ΔT in Eq. (S1.1), leading to the following derivation:

$$\Delta S = 100\frac{T_0\Delta O - O_0\Delta T}{T_0\cdot(T_0+\Delta T)} = 100\frac{T_0\Delta T - O_0\Delta T}{T_0\cdot(T_0+\Delta T)} = 100\frac{(T_0 - O_0)\Delta T}{T_0\cdot(T_0+\Delta T)}$$

$$= 100\frac{T_0 - O_0}{T_0}\cdot\frac{\Delta T}{T_0+\Delta T} = 100\left(1-\frac{O_0}{T_0}\right)\frac{\Delta T}{T_0+\Delta T} \qquad \text{(S1.2)}$$

$$= (100 - S_0)\frac{\Delta T}{T_0+\Delta T}.$$

The ΔdeoxyHb null curve in Fig. 1a is a plot of the ΔS vs. ΔT functional dependence of Eq. (S1.2).

Note that the Eq. (S1.2) dependence of ΔS on ΔT has a hyperbolic functional form. This result is intuitively plausible, because HbO_2Sat will monotonically rise as oxyHb is added with deoxyHb held constant, approaching 100% as a limiting value. In addition, the Eq. (S1.2) expression satisfies the physical requirement that HbO_2Sat falls to 0% (i.e., that $\Delta S = -S_0$) when $\Delta T = -O_0$ (i.e., when all of the initially present oxyHb is removed).

ΔoxyHb = 0:

The constraint is $\Delta O = 0$, which means that the first term in the numerator of Eq. (S1.1) vanishes. This gives us:

$$\Delta S = 100\frac{T_0\Delta O - O_0\Delta T}{T_0\cdot(T_0+\Delta T)} = 100\frac{-O_0\Delta T}{T_0\cdot(T_0+\Delta T)} = \left(-100\frac{O_0}{T_0}\right)\frac{\Delta T}{T_0+\Delta T} \qquad \text{(S1.3)}$$

$$= -S_0\frac{\Delta T}{T_0+\Delta T}.$$

The ΔoxyHb null curve in Fig. 1a is a plot of the ΔS vs. ΔT functional dependence of Eq. (S1.3).

Here too, the ΔS vs. ΔT function is a hyperbola. In this case the HbO_2Sat value falls to 0% in the limit as positive ΔT increases without bound, and it becomes 100% (i.e., $\Delta S = 100 - S_0$) for ΔT equal to $-D_0$ (i.e., when all of the initially present deoxyHb is removed).

ΔHbO_2Exc = 0:

In this case the constraint is $\Delta E = \Delta D - \Delta O = 0$, or $\Delta O = \Delta D$, from which it follows that $\Delta T = 2\Delta O$. Then we can replace ΔO with $\Delta T/2$ in Eq. (S1.1), which leads to:

$$\begin{aligned}\Delta S &= 100\frac{T_0\Delta O - O_0\Delta T}{T_0\cdot\left(T_0+\Delta T\right)} = 100\frac{T_0\dfrac{\Delta T}{2} - O_0\Delta T}{T_0\cdot\left(T_0+\Delta T\right)} = 50\frac{T_0\Delta T - 2O_0\Delta T}{T_0\cdot\left(T_0+\Delta T\right)}\\ &= 50\frac{\left(T_0 - 2O_0\right)\cdot\Delta T}{T_0\cdot\left(T_0+\Delta T\right)} = 50\frac{T_0-2O_0}{T_0}\cdot\frac{\Delta T}{T_0+\Delta T} = 50\left(1-\frac{S_0}{50}\right)\frac{\Delta T}{T_0+\Delta T}\\ &= \left(50-S_0\right)\frac{\Delta T}{T_0+\Delta T}.\end{aligned} \tag{S1.4}$$

The ΔHbO_2Exc null curve in Fig. 1a is a plot of the ΔS vs. ΔT functional dependence of Eq. (S1.4).

Once again, the result is a hyperbolic dependence of ΔS on ΔT. The value of HbO_2Sat falls to 50% in the limit as (positive) ΔT increases without bound. (If, hypothetically, S_0 were <50%, the hyperbola would be a monotonically increasing function of ΔT.)

In what may seem like a discrepancy with the preceding descriptions, direct inspection of plots such as Fig. 1a gives the impression that the ΔdeoxyHb, ΔoxyHb and ΔHbO_2Exc null curves, or secondary axes, are straight lines. The limited ranges of values for ΔHbO_2Sat and ΔtotalHb in the figures is the reason why their curvature is not apparent. However, for completeness it should also be noted that, of the ten unique coordinate systems for pairings of Hb components, the ΔHbO_2Sat-vs.-ΔtotalHb case is the only one that has hyperbolic null curves for all three of the other components. In every other case, at least one of the null curves is a straight line. Specifically, in the six coordinate systems that do <u>not</u> have ΔHbO_2Sat as one of the primary axes (e.g., the ΔoxyHb-vs.-ΔdeoxyHb system of [1]), all three secondary axes are linear. In contrast, in the ΔHbO_2Sat-vs.-(ΔdeoxyHb, ΔoxyHb, or ΔHbO_2Exc) systems the ΔtotalHb null curve is linear and the other two are hyperbolas. The latter finding is intuitively understandable in addition to being mathematically derivable:

- If, for example, the concentration of oxyHb increases without bound while that of deoxyHb is fixed (i.e., here we consider points on a ΔdeoxyHb null curve), HbO_2Sat will never reach 100% but will approach it asymptotically. Asymptotic approach to a limiting value similarly follows for other cases in which a component <u>other than</u> totalHb is fixed.

- In contrast, if totalHb is the component whose concentration is fixed, then there is a mathematical (as opposed to physiological) upper limit to the amount by which the oxyHb concentration can increase. This follows because holding the totalHb concentration constant entails removing a molecule of deoxyHb for every one of oxyHb that is added, and there is a finite amount of deoxyHb available for removal. When the last deoxyHb molecule is replaced with oxyHb, HbO_2Sat is 100%. At that point, no further increase in oxyHb that does not also raise the totalHb concentration is possible.

2. Network Coefficient-Dependent Patterns of Disease (Breast Cancer) Sensitivity.

While the data values in Fig. 2 are for non-cancer subjects, those in Supplementary Fig. 1 show varied disease sensitivity patterns for the breast-cancer subjects. Plotted are parameters related to breast-cancer diagnostic potential, for the (top row to bottom) transition probability, post-transition dwell time, ΔHbO_2Sat flux and ΔtotalHb flux. Plotted in the four columns (left to right) are the T group-mean coefficient value, the group-mean T–U difference, and unequal-variance t-test *p*-values for the null hypotheses T – U = 0 and L – R = 0. Markedly dissimilar patterns of T and T–U coefficient values are evident (inter-coefficient |correlations| are in the range of 0.12-0.62 and 0.04-0.55, respectively). Also, while every considered network coefficient exhibits high disease sensitivity (i.e., there are 38, 54, 84, and 65 transition types with significant differences between T and U adjacency-matrix values, and only 6, 12, 3, and 0 significant differences between L and R values), the adjacency matrices considered have markedly different patterns of sensitivity (range of inter-coefficient |correlation| values is 0.0070-0.42).

3. Properties of Hb Signal Component-Amplitude Co-dependence Plots.

An immediately apparent feature of Fig. 3a is that the 10 points in each sector fall nearly on a straight line, giving the set of lines an overall shape similar to the spokes of a wheel. On first take, such structure appears to conflict with the finding of a weak correlation (r = -0.246) between the adjacency matrices for the same quantities, plotted in Fig. 2c and Fig. 2d. Consistency between the findings is apparent when it is appreciated that the slopes observed are State dependent: a single linear regression applied to all 100 points in Fig. 3a yields the noted low correlation, while regressions that consider only points in one sector give $|r|$ values in the range of 0.981-0.9998. Further inspection reveals two additional trends that are shown assignable to adjustments of the ΔtotalHb signal.

One of the noted trends is the appearance of two groupings of 5 points along each spoke, one group lying farther from the origin and in most cases well-separated, and the other nearer the origin and more densely packed together. Because adjustments to both Hb components occur in all spokes, the pattern of groupings seen could arise from adjustment to either or both components. Closer examination, however, reveals that the trend seen is assignable to whether the magnitude of ΔtotalHb adjustment upon a transition is sufficient to produce a change in its algebraic sign. For pre-transition States 10 and 1-4 (i.e., those that have ΔtotalHb<0, left of the origin in Fig. 3a), the farther-from-origin points along a given spoke are those for post-transition States 10 and 1-4, while the bunched-together points are those for post-transition States 5-9; the converse is true for pre-transition States 5-9 (i.e., those that have ΔtotalHb>0). That is, in every case the well-spaced points farther from the origin correspond to transition types in which the algebraic sign of ΔtotalHb does not change, while the five tightly grouped nearer the origin are for the transition types in which the algebraic sign of ΔtotalHb reverses.

The second trend observed recognizes that the sequence of post-transition States (i.e., symbol shapes) is independent of the pre-transition state and of which pair of Hb components is considered. Proceeding from left to right in Fig. 3a (e.g., along the solid blue State-2 spoke), that sequence is 2/3, 1, 4, 10, 5, 9, 6, 8/7. Referring back to the State definitions diagram of Fig. 1a, we see that, proceeding from the ends of the sequence to the center, associated pairs (e.g., States 2 and 7) are reciprocals of each other. By this we mean that they are diametrically opposed in the diagram and they have opposite algebraic signs for all five Hb components (ΔdeoxyHb<0 in State 2 and >0 in State 7, etc.). This sequence correlates with the State-dependent post-transition mean value of ΔtotalHb, and not to those of the other components (Supplementary Note 5). Thus, both trends are assignable to the influence of adjustments to ΔtotalHb.

Other factors that can be shown to depend on the algebraic sign of ΔtotalHb are the overall amplitude of adjacency-matrix values, and their disease dependence, as shown in Table 1. The tabulated findings are consistent with the plausible assumption that it is energetically more favorable to maintain a steady-state condition though smaller adjustments in blood volume than through larger ones. We further note that the observed shift in transition probabilities in favor of types that do not undergo changes in ΔtotalHb algebraic sign, together with an increase in the overall amplitude of the Hb-signal, is consistent

with an expected inflammatory response in T subjects. The ability to define such trends from steady-state measures represents a novel capability.

4. Variable and Invariant Properties of Hb Signal Component-Amplitude Co-dependence Plots.

As a demonstration of complementarity between pre- and post-transition network coefficient co-dependencies, in Supplementary Fig. 2 we show a plot of post-transition ΔHbO_2Sat vs. post-transition ΔtotalHb. Comparison to Fig. 3a illustrates a point asserted in the description of Fig. 3: the two sets of 100 markers form the same spatial pattern, but a given transition type lies at different position in the second plot than in the first. Thus, the data points on a selected "spoke" share a common pre-transition State in Fig. 3a, and a common post-transition State in Supplementary Fig. 2.

5. Impact of Hb-State definitions on observed co-dependences between Hb-component amplitudes.

Co-dependency plots such as those in Fig. 3a and Supplementary Fig. 2 display a number of features that are shown for the first time in this report: the ten mean-value points in each pre-transition State sector are arranged along a straight line; the left-to-right sequence of points is the same in each sector; the points in each sector divide into two groups of five, those nearer the origin corresponding to transition types in which the ΔtotalHb component undergoes a change in algebraic sign. Before attributing biological significance to such findings, it is important to determine whether they could be mathematically inevitable consequences of the way that the Hb States have been defined, which introduce a fixed set of geometrical relationships among the State-specific regions of the ΔHbO_2Sat-vs.-ΔtotalHb coordinate system (and of the corresponding system for any other choice of primary axes).

We have examined the possibility that features may be geometrically forced by performing a set of computations on a model consisting of a unit-radius disk centered at the origin of the ΔHbO_2Sat-vs.-ΔtotalHb coordinate system (Fig. 1a, Supplementary Fig. 13). The disk model can be taken as an idealized representation of an image time-series point cloud (Fig. 1b-c) in the limit of a uniform distribution of data points within a finite distance from the origin. Thus, the model is even simpler than the apparently unstructured data it is based on, which do show radial and angular (and disease) dependences; as shown here, however, it is sufficient for our purposes.

A. Physiological correlates of the "bicycle wheel" plot (Fig. 3a) data-point sequence.

As noted above, inspection of the Fig. 3a and Supplementary Fig. 2 plot revealed that the line segment formed by the data points in each State sector has the same sequence of data points. In Results it is further noted that examination of analogous plots for coordinate systems comprising all possible unique pairings of Hb-signal components showed that the same sequence occurs in all ten cases. Here we show that a geometric property of the uniform-disk model aids in interpreting the observations; in doing so, the result also addresses concerns that the model may be too simplified to permit reproduction of relevant properties of the system that we use it to approximate.

For the computation, the centroid coordinates for each State sector of the model were found using the analytical formula for the centroid of a circle sector: $A = 4r\sin[(\theta_2-\theta_1)/2]/[3(\theta_2-\theta_1)]$ [2], where r is the disk radius and θ_1 and θ_2 are the sector's angular limits (e.g., for State 1, $\theta_1 \approx 125°$ and $\theta_2 \approx 150°$). The x- and y- coordinates for a given centroid are $x_0 = A\cos[(\theta_1+\theta_2)/2]$, $y_0 = A\sin[(\theta_1+\theta_2)/2]$, and x_0 and y_0 are the mean values of ΔtotalHb and ΔHbO_2Sat, respectively, for the considered disk sector. The mean values for the other Hb components were found using the analytic formula for distance from a point to a line: $d = |ax_0 + by_0 + c|/(a^2 + b^2)^{1/2}$ [3], where (x_0,y_0) are the centroid coordinates, $ax + by + c = 0$ is the general equation for a straight line, and the hyperbolic ΔdeoxyHb, ΔHbO_2Sat and ΔtotalHb null curves (Supplementary Note 1) are approximated by straight lines.

A plot of Hb-component centroid value vs. disk-sector number (Fig. 1a, Supplementary Fig. 13) is shown in Supplementary Fig. 10. Comparisons of the centroid-value sequences to the invariant real-data sequence for the "spokes" in Fig. 3a and Supplementary Fig. 2 reveal qualitative agreement between the latter and the sequence of the disk-model ΔtotalHb mean values and, equal noteworthy, no evident relationship to the trends for any other components. In contrast, dependences on the values of multiple components could be expected for a sequence determined by the coordinate-system geometry. The selective concordance thus indicates that the observed invariant sequence is biologically driven. The suggestion is that ΔtotalHb values in the post-transition State influence the distributions of points from which transitions preferentially begin in the pre-transition State (Fig. 3a), and, likewise, that ΔtotalHb values in the pre-transition State influence the distributions of points that transitions preferentially lead to in the post-transition State (Supplementary Fig. 2). We next consider more explicitly the question of how the co-dependence plots might be expected to appear if geometry were the controlling factor.

B. Essential features of "wheel spokes" plot do not follow trivially from State and transition definitions. An intuitively plausible way in which findings reported as being physiologically based could instead be consequences of the coordinate-system geometry is that the number (or probability, or rate) of transitions from one point to another in the ΔHbO$_2$Sat-vs.-ΔtotalHb space is simply proportional to the distance between them, or to some monotonically increasing function of the distance. Indeed, this suggestion gains apparent support from casual inspection of findings such as Supplementary Fig. 11, wherein the Fig. 1b-c cloud plots are modified to show the percentage of State-7 and State-9 events that participate in either 7→9 or 9→7 transitions. The quantity plotted in Supplementary Fig. 11 is $100 \cdot N_{7\leftrightarrow 9}/N_{\mathrm{all}}$, where N_{all} is the total number of occurrences of every pairing of ΔtotalHb and ΔHbO$_2$Sat values (Fig. 1b-c), and $N_{7\leftrightarrow 9}$ is the number of only those occurrences that take part in 7→9 or 9→7 transitions. It is seen that the percentage of State-9 events participating in the specified transition types is larger for positions in the State-9 sector that lie close to the State-7 sector, than it is for State-9 locations farther away from State 7. Accordingly, we investigated the hypothesis of simple geometric distance-determined network coefficients using the uniform-disk model.

The first computational step was to randomly select, from spatially uniform distributions, a large number of points in each State sector of the ΔHbO$_2$Sat-vs.-ΔtotalHb coordinate system (it was empirically found that 4000 points per sector gave excellent repeatability). In order to achieve spatial uniformity, angular coordinate values (θ) were obtained by sampling from a uniform distribution, and radial coordinate values (r) by computing the square roots of values sampled from a uniform distribution [4]. The (r,θ) coordinates were converted to (x,y) format via the formulas $x = r\cos\theta$, $y = r\sin\theta$. For each of the 100 transition types, a pre-transition mean-value point consistent with the geometric-distance assumption was obtained by combining the sets of randomly sampled coordinates with the previously computed sector centroid coordinates, as follows:

i. Distances between every randomly sampled point in the pre-transition State and the geometric centroid of the post-transition State are computed:

$$d_k^{ij} = \sqrt{\left(x_k^j - x_0^i\right)^2 + \left(y_k^j - y_0^i\right)^2}, \quad i = 1-10,\ j = 1-10, k = 1-4000, \qquad \text{(S5.1)}$$

where $\left(x_k^j, y_k^j\right)$ are coordinates for the kth randomly sampled point in State j (i.e., pre-transition State), and $\left(x_0^i, y_0^i\right)$ are the State-i (i.e., post-transition State) centroid coordinates. As noted in the previous section, the coordinate system is defined such that the x and y coordinates of any point are values for ΔtotalHb and ΔHbO_2Sat, respectively.

ii. Mean pre-transition values of both components, inversely weighted by distance from the post-transition centroid, are computed as:

$$\bar{x}_\alpha^{ij} = \frac{\sum_{k=1}^{4000} \frac{x_k^j}{\left(d_k^{ij}\right)^\alpha}}{\sum_{k=1}^{4000} \left(\frac{1}{d_k^{ij}}\right)^\alpha}, \quad \bar{y}_\alpha^{ij} = \frac{\sum_{k=1}^{4000} \frac{y_k^j}{\left(d_k^{ij}\right)^\alpha}}{\sum_{k=1}^{4000} \left(\frac{1}{d_k^{ij}}\right)^\alpha}, \quad i = 1-10,\ j = 1-10. \tag{S5.2}$$

The exponent α in Eq. (S5.2) is a parameter that governs the rate at which the importance of pre-transition State points falls as their distance to the post-transition State centroid increases; in particular, the contributions of short-distance points grow as α increases.

A plot of pre-transition State coordinates $\left(\bar{x}_3^{ij}, \bar{y}_3^{ij}\right)$ for all 100 transition types is shown in Supplementary Fig. 12. The identities of the transition types are encoded by symbol shape and color/fill, in the manner specified in Fig. 3b. The effect of in(de)creasing the value of α is to in(de)crease the distances among the points in each sector, but qualitatively the shapes of the 10-point structures and the relative positions of points within them are not affected. In contrast to the plot in Fig. 3a, the arrangement of points is not linear in any sector. In addition, the distance-from-origin sequence is different in every sector, in no case does it agree with that seen in the breast-data result (the closest approximation is found in Sectors 3 and 8), and in no case is there a sharp separation into two 5-point subsets (the closest approximation is found in Sectors 2 and 7). In all, the Supplementary Fig. 12 findings indicate that the suggested geometric factor is not sufficient to account for the structure of the Fig. 3a plot.

C. Dependence of extrapolated "wheel spoke" intercepts on Hb State

With the aim of gaining an expanded appreciation of feature behaviors seen in Fig. 3a, here we address a property of the Fig. 3a plot that has not previously been mentioned, but that readers are likely to have noticed. Namely, that if the points in each State sector are fit with a straight line, and the line is extended until it intercepts the ΔHbO_2Sat axis (i.e., ΔtotalHb = 0), the intercept is near zero in six of the ten sectors (i.e., States 1-3 and 6-8) and far from zero in the other four (i.e., States 10, 4, 5 and 9). The cause that we have ascertained for this difference follows from a defining property of the Hb States.

It will be recalled from Results that the five points that lie nearest the origin on any "spoke" correspond to transition types in which the algebraic sign of ΔtotalHb reverses. (It also is intuitively reasonable that a sign-reversing transition would more easily be accomplished the closer the pre-transition ΔtotalHb amplitude is to zero.) As sketched in Supplementary Fig. 13, values of ΔtotalHb that are smaller, in absolute value, than a specified threshold are confined to finite-area regions in States 1-3 and 6-8, but in States 10, 4, 5 and 9 they may lie anywhere within strips that are (in principle) infinitely long. (The gray rectangle in Supplementary Fig. 13 illustrates this for the case of $|\Delta totalHb| \leq 0.2$.) Thus the States 1-3, 6-8 "spokes" must approach the origin to intercept the ΔHbO_2Sat axis, while the States 10, 4, 5, 9 "spokes" need not do so.

6. Simultaneous Evaluation of Algebraic-Sign Effects of All Hb-Signal Components.

As a follow-up to the Fig. 3a demonstration that the clustering of points depends on the algebraic sign of ΔtotalHb, it is of interest to determine whether network coefficients in general exhibit dependencies on the algebraic sign of any Hb component. This led us to design a computation that can evaluate, for any network coefficient, the dependence of adjacency matrix values on the algebraic-sign properties of all five Hb components (Supplementary Note 7). The algorithm computes, for any specified adjacency matrix **A**, all 100 ratios $A_{i'j}/A_{ij}$, where matrix rows i and i' contain data values for reciprocal post-transition States (e.g., for $i = 1$, $i' = 6$). Thereby, the ratios compare adjacency-matrix values between pairs of transition types having opposite algebraic-sign properties for all five Hb components. Because each Hb component has a distinct transition-dependent pattern to its algebraic sign [1], a comparison between the computed ratios for each adjacency matrix and ideal templates corresponding to the five components' algebraic-sign patterns identifies which Hb-component best fits the computed ratio.

Results for representative cases are shown in Supplementary Fig. 3: transition probability (Panel a), pre-transition dwell time (b), and ΔoxyHb pre-transition mean value (c). Every plotted data value is a ratio of two adjacency-matrix elements, $A_{i'j}/A_{ij}$, where the numerator matrix rows are permuted in a specific order (i' = 6-10, then 1-5) while the denominator rows are in the original order (i = 1-10). Overlaid on each panel is a line patterning representing the algebraic sign behavior of ΔtotalHb, which, among the five Hb-signal components, is the one that most closely matches the spatial patterns of contrast features. The '0' and '1' annotations in (a) identify the sign-preserving and sign-reversing transition types, respectively. Further analysis shows that the pre- and post-transition mean values for all Hb components have ratio patterns similar to that of (c).

While the finding that the predominant contrast pattern is that of totalHb is unsurprising, given known mechanisms for modulation of blood delivery to tissue, it is noteworthy that here its impact is demonstrated in volumetric images computed from noninvasive steady-state measurements. Further, the Supplementary Fig. 3 patterns can be regarded as "hidden" phenomenology, in that the algebraic-sign dependence evident in the adjacency-matrix ratios is not obvious upon inspection of the corresponding primary matrices.

7. Method of generating an array of ratios of adjacency-matrix values.

In the findings presented in Supplementary Fig. 3, the numerical value in each array element (which is encoded by the color of the corresponding square) is a ratio of two adjacency-matrix values. It is further reported, but not proved, that every such ratio compares two transition types that simultaneously differ with respect to the sign-change properties of all five Hb components (Supplementary Note 6). For the benefit of interested readers, here we present a mathematical derivation of the ratio computation.

The first operational step is to generate the following 10×10 permutation matrix [5]:

$$\mathbf{P}_{10} = \begin{bmatrix} 0 & 0 & 0 & 0 & 0 & 1 & 0 & 0 & 0 & 0 \\ 0 & 0 & 0 & 0 & 0 & 0 & 1 & 0 & 0 & 0 \\ 0 & 0 & 0 & 0 & 0 & 0 & 0 & 1 & 0 & 0 \\ 0 & 0 & 0 & 0 & 0 & 0 & 0 & 0 & 1 & 0 \\ 0 & 0 & 0 & 0 & 0 & 0 & 0 & 0 & 0 & 1 \\ 1 & 0 & 0 & 0 & 0 & 0 & 0 & 0 & 0 & 0 \\ 0 & 1 & 0 & 0 & 0 & 0 & 0 & 0 & 0 & 0 \\ 0 & 0 & 1 & 0 & 0 & 0 & 0 & 0 & 0 & 0 \\ 0 & 0 & 0 & 1 & 0 & 0 & 0 & 0 & 0 & 0 \\ 0 & 0 & 0 & 0 & 1 & 0 & 0 & 0 & 0 & 0 \end{bmatrix}. \qquad \text{(S7.1)}$$

As is true in general for permutation matrices, the product of $\mathbf{P}_{10}$ and an arbitrary matrix $\mathbf{X}$, i.e, $\mathbf{X}_{p,r} = \mathbf{P}_{10}\mathbf{X}$, preserves the numerical values in $\mathbf{X}$ but rearranges, or permutes, the order of the rows. (In the complementary product $\mathbf{X}_{p,c} = \mathbf{X}\mathbf{P}_{10}$, the columns of $\mathbf{X}$ are re-ordered.) For the particular matrix defined in Eq. (S7.1) (which is one of 10! ≈ 3.6 million possible order-10 permutation matrices [5]), the result of the indicated multiplication is:

$$\mathbf{P}_{10}\mathbf{X} = \mathbf{P}_{10}\begin{bmatrix} a1 & a2 & a3 & a4 & a5 & a6 & a7 & a8 & a9 & a10 \\ b1 & b2 & b3 & b4 & b5 & b6 & b7 & b8 & b9 & b10 \\ c1 & c2 & c3 & c4 & c5 & c6 & c7 & c8 & c9 & c10 \\ d1 & d2 & d3 & d4 & d5 & d6 & d7 & d8 & d9 & d10 \\ e1 & e2 & e3 & e4 & e5 & e6 & e7 & e8 & e9 & e10 \\ F1 & F2 & F3 & F4 & F5 & F6 & F7 & F8 & F9 & F10 \\ G1 & G2 & G3 & G4 & G5 & G6 & G7 & G8 & G9 & G10 \\ H1 & H2 & H3 & H4 & H5 & H6 & H7 & H8 & H9 & H10 \\ I1 & I2 & I3 & I4 & I5 & I6 & I7 & I8 & I9 & I10 \\ J1 & J2 & J3 & J4 & J5 & J6 & J7 & J8 & J9 & J10 \end{bmatrix}$$

$$= \begin{bmatrix} F1 & F2 & F3 & F4 & F5 & F6 & F7 & F8 & F9 & F10 \\ G1 & G2 & G3 & G4 & G5 & G6 & G7 & G8 & G9 & G10 \\ H1 & H2 & H3 & H4 & H5 & H6 & H7 & H8 & H9 & H10 \\ I1 & I2 & I3 & I4 & I5 & I6 & I7 & I8 & I9 & I10 \\ J1 & J2 & J3 & J4 & J5 & J6 & J7 & J8 & J9 & J10 \\ a1 & a2 & a3 & a4 & a5 & a6 & a7 & a8 & a9 & a10 \\ b1 & b2 & b3 & b4 & b5 & b6 & b7 & b8 & b9 & b10 \\ c1 & c2 & c3 & c4 & c5 & c6 & c7 & c8 & c9 & c10 \\ d1 & d2 & d3 & d4 & d5 & d6 & d7 & d8 & d9 & d10 \\ e1 & e2 & e3 & e4 & e5 & e6 & e7 & e8 & e9 & e10 \end{bmatrix}. \qquad \text{(S7.2)}$$

Eq. (S7.2) illustrates that left-multiplying any 10×10 matrix by $\mathbf{P}_{10}$ has the effect of interchanging the rows of the upper and lower halves of the matrix, while having no effect on the ordering of the columns, or on that of the rows within each half.

For a selected adjacency matrix **W** (i.e., a matrix of network-edge weights), we compute the ratios matrix as:

$$\mathbf{R}^{\mathbf{W}} = (\mathbf{P}_{10}\mathbf{W}) \oslash \mathbf{W}, \qquad \text{(S7.3)}$$

where $\oslash$ denotes Hadamard division, i.e., $\mathbf{K} \oslash \mathbf{L} = \mathbf{M}$ means that $m_{ij} = k_{ij}/l_{ij}$ [6]. Substituting Eq. (S7.2) into Eq. (S7.3) gives:

$$\mathbf{R}^{\mathbf{W}} = \begin{bmatrix} w_{61}/w_{11} & w_{62}/w_{12} & \cdots & w_{610}/w_{110} \\ w_{71}/w_{21} & w_{72}/w_{22} & \cdots & w_{710}/w_{210} \\ \vdots & \vdots & \ddots & \vdots \\ w_{41}/w_{91} & w_{42}/w_{92} & \cdots & w_{410}/w_{910} \\ w_{51}/w_{101} & w_{52}/w_{102} & \cdots & w_{510}/w_{1010} \end{bmatrix}, \tag{S7.4}$$

That is, every element of $\mathbf{R}^{\mathbf{W}}$ is a ratio of two elements of **W**.

To verify the additional claim that every ratio value in $\mathbf{R}^{\mathbf{W}}$ considers two transition types that have opposite algebraic sign-change properties for all five Hb-signal components, here we focus on the case of pre-transition State 4, and in Table S2 we explicitly show the sign-change property of every component, for each of the ten transition types. As shown by Eq. (S7.4), the ratio computation pairs the 4→1 (1st row of Table S2) and 4→6 (6th row) transition types, 4→2 and 4→7, etc. Accordingly, direct comparison of the 1st and 6th (red), 2nd and 7th (orange), etc., rows of Table S2 confirms that each such pairing of transition types has opposite sign-change properties for all five Hb-signal components. The same is found for the sign patterns for the other pre-transition States (not shown).

Because adjacency-matrix ratios thus consider the algebraic signs of all Hb components, the patterns of high and low values in results such as Fig. 3a and Supplementary Fig. 3 could be correlated with the sign-change pattern of any of them, or with more than one. Therefore, the observation that in every case only one component (i.e., ΔtotalHb) has a dominant effect is not algorithmically required; rather, it is a manifestation of a biology-based pattern in the considered adjacency matrices. In many cases, however, the noted pattern is effectively "hidden" by other, larger-amplitude and thus more obvious trends. It is therefore not apparent by inspection of the primary data but can be revealed by the ratio computation.

8. Flux vs. Post-transition Mean Value Plots for All Possible Pairings of Hb Components.

Results plotted in Supplementary Fig. 4 are an extension of Fig. 3d findings, to the full set of co-dependences between flux and post-transition mean values for all 25 two-component pairings. This information is presented in support of the claim that structured behaviors are observed for all pairings.

Group-mean data for the non-cancer subjects (average of L and R) is used here, as it was for the representative adjacency matrices shown in Fig. 2.

In Supplementary Fig. 4, for 13 of the 25 subplots, the plotted points are color- and shape-labeled in the same manner as Fig. 3, to allow identification of the transition types. For the remaining 12 subplots, the alternative rendering uses black dots for all data points, to facilitate appreciation of aspects of the spatial distributions that are obscured by the size of the colored markers. Every subplot in the first row of the 5×5 array has ΔdeoxyHb flux as the y-coordinate, and in the first column every subplot has ΔdeoxyHb post-transition mean values as the x-coordinate. Likewise, x(y)-coordinates in the second through fifth columns (rows) are post-transition mean values (fluxes) of ΔHbO_2Exc, ΔoxyHb, ΔHbO_2Sat and ΔtotalHb, respectively. Thus, inspection reveals that the lower right-corner panel, which is a plot of ΔtotalHb flux vs. ΔtotalHb post-transition mean value (average of L and R groups), has a patterning of markers closely resembling that seen in Fig. 3d (T group).

It is evident that every subplot contains a structured "constellation" of 100 data points, rather than a random cluster. The groups of points for a selected pre- or post-transition State likewise form structured sets, which in some cases are near linear (e.g., row 1, column 4) and in others distinctly oval (e.g., row 4, column 5). Pairings that contain hyperbolic co-dependences are found only in the fifth row, i.e., the plots of ΔtotalHb flux vs. post-transition mean values for any Hb component. In the black-dot plots, potentially interesting variations in the density of the 100-point distributions (e.g., row 3, column 5) are identifiable, as are groupings of points that form linear (e.g., row 2, column 2) or closed-loop (e.g., row 4, column 4) structures without necessarily sharing a common pre- or post-transition State.

Examination of the analogous pre- vs. post-transition mean value plots (not shown) revealed that in this case too, there is a structured co-dependence for every pairing of Hb components. Additionally, the 5×5 array contains a degree of symmetry not seen in Supplementary Fig. 4: the pattern of points in any row-*i*, column-*j* subplot is the geometric transpose of the row-*j*, column-*i* pattern. Accordingly, hyperbolic trends are found in every column-5 subplot as well as in every row-5 one (for a total of nine pairings). However, the noted transpose relationship applies only at the level of the complete 100-point spatial distribution and does not carry over to individual transition types. Instead, what is found is a

complementarity similar to that between Fig. 3a and Supplementary Fig. 2, so that a structure consisting of same-color markers in one subplot has a counterpart consisting of same-shape markers in its transpose.

9. Quantitative Considerations from Highly Structured Dependences Observed in Heterogeneous Co-Dependence Plots

The observation of highly structured co-dependences in some pairings of network coefficients, as in the sets of data points with excellent fits to hyperbolic curves in Fig. 3c-d, allows us to consider whether structured patterns occur also in analytic parameters (e.g., coordinates of hyperbola vertices and foci) of the fitted functions. The presence of such structure (as opposed to, say, a random scatter of vertex-focus coordinates) of would lend support to the suggestion that the distributions of co-dependence values reflect the operation of biological drivers of tissue hemodynamics.

A. Statistical Significance of Hemodynamic-State Differences Among Fig. 3 Hyperbolic Trends

A prerequisite to undertaking any higher-order quantitative analysis of findings such as the varied hyperbolic trends seen in Fig. 3c-d is to establish that those trends actually are different. Here we describe the combination of unpaired and paired t-tests used to show that differences between trends observed for different pre- and post-transition States in Fig. 3c-d are statistically significant.

For the Fig. 3c result, unpaired t-tests were used to evaluate differences between the ΔHbO_2Sat distributions for nearest-neighbor pairs of pre-transition States (e.g., filled magenta (State 10) and unfilled cyan (State 9) plot symbols). The differences are highly significant ($p = 1.2\times10^{-9} - 9.3\times10^{-4}$) in all cases except for the pairing of States 1 (filled red symbols) and 9, where statistical significance is achieved for the T breast group ($p = 0.027$), but not for U ($p = 0.80$) or the non-cancer subjects ($p = 0.16$, left- and right-breast data combined). For the corresponding ΔtotalHb distributions, statistical significance was evaluated via paired t-tests (e.g., the set of differences between ΔtotalHb values for the $1\rightarrow k$ and $9\rightarrow k$ transition types, $k = 1–10$). The differences are highly significant ($p = 5.5\times10^{-5} - 4.3\times10^{-4}$) in all cases except for the States 1 and 2 (filled blue) pair, where statistical significance is achieved for T ($p = 0.034$), but not for U ($p = 0.11$) or the non-cancer subjects ($p = 0.085$). Thus, every nearest-neighbor pair (and by extension, all more-distant pairs) is well-separated in at least one coordinate-system dimension.

Statistical testing for the Fig. 3d data must consider both plot variables together, owing to the obliquity of the 2D distribution. Paired t-tests were carried out on the 2D distances between the plotted points, e.g., $[(Y_{1\rightarrow k} - Y_{2\rightarrow k})^2 + (X_{1\rightarrow k} - X_{2\rightarrow k})^2]^{1/2}$ for comparisons between nearest-neighbor pre-transition States, $[(Y_{k\rightarrow 1} - Y_{k\rightarrow 2})^2 + (X_{k\rightarrow 1} - X_{k\rightarrow 2})^2]^{1/2}$ for post-transition States comparisons, where Y and X denote the ΔtotalHb flux and post-transition mean value, respectively. To generate a quantity that can have either algebraic sign—thereby allowing us to use 'mean(paired difference) = 0' as the null hypothesis—each distance either was left as a positive value or was multiplied by -1, depending on which data point is farther from the origin (e.g., '+' ('-') if $(X_{1\rightarrow k},Y_{1\rightarrow k})$ is farther from (closer to) the origin than $(X_{2\rightarrow k},Y_{2\rightarrow k})$). The differences are highly significant (pre-transition State comparisons: $p = 1.4\times10^{-11} - 0.0097$; post-transition comparisons: $p = 3.7\times10^{-11} - 0.0029$) in all cases except for the States 2 and 3 (filled green) pairs ($p = 0.060 - 0.65$), which are largely superimposable in Fig. 3d.

B. Vertex-and-Focus Plots Derived from Heterogeneous Co-dependence Plots.

For the specific case of hyperbolic co-dependence, Supplementary Fig. 5 illustrates how a high degree of data compression can be achieved when depicting a highly structured behavior. Clear evidence of State-dependent structure, which differs in detail for co-dependences between pairings of mean values, and between a flux and a mean value, is present in the compact representation.

Plotted in Supplementary Fig. 5a are the vertices (circles) and foci (diamonds) of the hyperbolas fitted to the ten sets of fixed pre-transition State points (see Fig. 3b) in Fig. 3c. Thus, for example, the filled red circle and diamond are the vertex and focus, respectively, for the hyperbola fitted to the ten filled red symbols in Fig. 3c. Supplementary Fig. 5b is the corresponding vertex-and-focus plot for the hyperbolas fitted to the groupings of points plotted in Fig. 3d. The latter case has hyperbolic trends in sets of points with either a common pre- or post-transition State, and accordingly there are twenty vertex-focus pairs. Thus, for example, the filled red circle and diamond in QII are the vertex and focus for the hyperbola fitted to the ten filled red markers (i.e., pre-transition State 1) in Fig. 3d, while those in QIII are for the hyperbola fitted to the ten circle markers (i.e., post-transition State 1).

It can be shown that a complete hyperbola can be constructed from a knowledge of the coordinates of a vertex and a focus [7]. However, more important than the degree of data compression achieved is the observation of patterns that may not be apparent by direct inspection of Fig. 3c-d (consequently, these

may be regarded as additional examples of “hidden” behaviors). One example of this is seen in the mean pre-transition ΔHbO_2Sat vs. mean post-transition ΔtotalHb results (Fig. 3c, Supplementary Fig. 5a), where the oppositely directed rotations between the curves for States 3 and 4 and those for States 10, 1 and 2 is more evident in Supplementary Fig. 5a than in Fig. 3c. A second example, seen in the ΔtotalHb flux vs. mean post-transition ΔtotalHb results (Supplementary Fig. 5b), is the displacement from the origin of the mean trends for vertices and foci in QI,III, but not in the corresponding trends in QII,IV. (In this context, we note that the dotted lines in Supplementary Fig. 5a-b are hand-sketched overlays intended to highlight the indicated trends and are not fitted regression lines.)

An additional benefit obtained from the vertex-and-focus representation is that it facilitates the determination of disease sensitivity in the hyperbolic co-dependences, as is demonstrated in Supplementary Fig. 5c-d for the ΔtotalHb flux vs. mean post-transition ΔtotalHb case. Plotted in Supplementary Fig. 5c are T – U inter-breast differences between vertex and focus coordinates (note that the ranges of values on the coordinate axes are halved with respect to those of Supplementary Fig. 5b), and the corresponding L – R differences are plotted in Supplementary Fig. 5d. Large differences, which also are seen to largely retain the structure seen in Supplementary Fig. 5b, are present in the cancer-subject differences.

10. Dependence of Hb-component flux magnitudes on transition Classes.

While Fig. 4 reveals a dependence of flux magnitude on transition Class for ΔtotalHb, results in Supplementary Fig. 6 extend this analysis to all of the Hb-signal components. It will be recalled that the Classes range from 0 to 5, and that the Class for a given transition type is equal to the number of Hb components that undergo changes in algebraic sign during the transition. Each curve is a plot of the average |flux| magnitude for the transition types in each of the six Classes for the indicated Hb component, normalized to that component’s maximum |flux| value. Qualitatively similar trends were obtained for all breast groups and all data averaging methods (Supplementary Note 12).

It is seen that Classes 3-5 have larger average |flux| magnitudes than Classes 0-2, for all five Hb-signal components. Additionally, in every case a bi-phasic trend is seen, with the |flux| value first rising with increasing transition Class and then reaching (ΔdeoxyHb, ΔHbO_2Exc, ΔHbO_2Sat) or approaching (ΔoxyHb, ΔtotalHb) a maximum limiting value. As noted in Results, we hypothesized that the consistent

trend reflects a Class-dependent impact of non-observable tissue drivers on the macroscopic observable quantities, with a more consistent set of influences across transition types in the saturation phase. This interpretation gains support from the subsequent observation of Class-dependent trends in findings from the vector-amplitude analyses.

11. Rationale for Vector-Amplitude Computations and Lineweaver-Burk (L-B) Plots.

Guiding the vector amplitude computations (Fig. 5, Supplementary Fig. 7) is the understanding that hemodynamic phenomenology is driven by a composite of enzymatic actions whose resultant magnitude is in some way proportional to the levels of molecular machinery present, and to the concentrations of the agents they act upon. Because the network coefficients considered in this report are influenced by the amplitudes and rates of enzyme-mediated reactions, it can be useful to consider how the different coefficients might best be aligned with such behaviors.

We began by conjecturing that flux measures are proportional to the magnitude of some composite of enzyme activities. The activity amplitudes are sensitive to prevailing substrate concentrations, with the effect of rendering some transition types more probable, and other less so. Invoking classical enzyme-action theory, we could expect that plots of variations in substrate concentration (i.e., transition probability) vs. enzyme activity (Hb-component flux values) should produce a saturable trend that is well approximated by a rectangular hyperbola, which in turn would yield a classical L-B plot. Findings that show the presence of such behaviors are presented in Results; however, it is also seen that this requires computation of a new metric which is a function of more than one "flux" measure.

12. Dependence of spatiotemporal measures on averaging methods.

The grand average (GA) method was, in recognition of its simplicity, the principal approach used to generate reported network parameters (e.g., Eqs. (6), (9), (10)). However, the natural pulsatile behavior of the vascular bed, intrinsic bias of NIRS measures to superficial structures, and structural heterogeneity of the breast make clear that volumetric time-series data could exhibit structured variability in either the temporal or spatial dimension. Here we present equations for the transition-dependent dwell times, and for the Hb-component mean values and fluxes, that recognize these dependences.

Summing first over time (thereby generating a time-averaged spatial map as an intermediate result) and then over volume yields expressions for the spatial mean of the temporal mean (SMTM). Thus:

$$\tau_{k\ \mathrm{SMTM}}^{(m)} = \frac{1}{N_v}\sum_{j=1}^{N_v}\left[\sum_{i=3-m}^{N_{TS}+1-m}\left(U_k\right)_{ij}\left(n_{s_1}^k\right)_{ij} \Big/ \sum_{i=3-m}^{N_{TS}+1-m}\left(U_k\right)_{ij}\right], \quad m=1,2, \tag{S12.1}$$

for the dwell times (m = 1,2 correspond to <u>pre</u>- and <u>post</u>-transition values, respectively), and

$$\phi_{k\ \mathrm{SMTM}}^{X} = \frac{1}{N_v}\sum_{j=1}^{N_v}\left[\sum_{i=1}^{N_{TS}}\left(U_k^X\right)_{ij}^{(3)} \Big/ \sum_{i=1}^{N_{TS}}\left(U_k\right)_{ij}\right] \tag{S12.2}$$

for the flux of Hb-signal component X. The corresponding SMTM formulas for <u>pre</u>- and <u>post</u>-transition mean values are obtained by substituting '(1)' or '(2)', respectively, for '(3)' in Eq. (S12.2).

In like manner, summing first over volume (thereby generating a volume-averaged time series) and then over time gives the formulas for the temporal mean of the spatial mean (TMSM) quantities:

$$\tau_{k\ \mathrm{TMSM}}^{(m)} = \frac{1}{N_{TS}-1}\sum_{i=3-m}^{N_{TS}+1-m}\left[\sum_{j=1}^{N_v}\left(U_k\right)_{ij}\left(n_{s_1}^k\right)_{ij} \Big/ \sum_{j=1}^{N_v}\left(U_k\right)_{ij}\right], \quad m=1,2, \tag{S12.3}$$

for the dwell times,

$$\phi_{k\ \mathrm{TMSM}}^{X} = \frac{1}{N_{TS}}\sum_{i=1}^{N_{TS}}\left[\sum_{j=1}^{N_v}\left(U_k^X\right)_{ij}^{(3)} \Big/ \sum_{j=1}^{N_v}\left(U_k\right)_{ij}\right] \tag{S12.4}$$

for the component-X flux, and formulas for <u>pre</u>- and <u>post</u>-transition mean values are obtained by substituting '(1)' or '(2)', respectively, for '(3)' in Eq. (S12.4).

A careful comparison of Eqs. (10) and (S12.4), for example, reveals that the GA and TMSM values for ϕ_k^X <u>would be</u> identical <u>if</u> the $\sum_{j=1}^{N_v}\left(U_k\right)_{ij}$ term in the latter equation had the same value at all time

steps, because in that case $\sum_{j=1}^{N_v}(U_k)_{ij}$ could be pulled out of the brackets in Eq. (S12.4), and we would have:

$$\begin{aligned}
\phi_{k\ \mathrm{TMSM}}^{X} &= \frac{1}{N_{TS}}\sum_{i=1}^{N_{TS}}\left[\sum_{j=1}^{N_v}\left(U_k^X\right)_{ij}^{(3)} \Big/ \sum_{j=1}^{N_v}\left(U_k\right)_{ij}\right] \\
&= \frac{1}{N_{TS}}\frac{1}{\sum_{j=1}^{N_v}\left(U_k\right)_{ij}}\sum_{i=1}^{N_{TS}}\sum_{j=1}^{N_v}\left(U_k^X\right)_{ij}^{(3)} \\
&= \frac{1}{N_{TS}\cdot C_{ki}}\sum_{i=1}^{N_{TS}}\sum_{j=1}^{N_v}\left(U_k^X\right)_{ij}^{(3)} \\
&= \frac{1}{c_k}\sum_{i=1}^{N_{TS}}\sum_{j=1}^{N_v}\left(U_k^X\right)_{ij}^{(3)} \\
&= \frac{1}{c_k}\sum_{j=1}^{N_v}\sum_{i=1}^{N_{TS}}\left(U_k^X\right)_{ij}^{(3)} = \phi_{k\ \mathrm{GA}}^{X}.
\end{aligned} \qquad \text{(S12.5)}$$

In Eq. (S12.5), $\sum_{j=1}^{N_v}(U_k)_{ij} = C_{ki}$ by Eq. (3), $c_k = \sum_{i=1}^{N_{TS}} C_{ki}$ (Eq. (4)) becomes $c_k = N_{TS}{\cdot}C_{ki}$ if C_{ki} has the same value at all time steps (i.e., for all values of *i*), and the commutativity of addition ensures that changing the order of the summations in the final line does not affect the result.

Reasoning parallel to that used in deriving Eq. (S12.5) leads to analogous conclusions for the TMSM pre- and post-transition dwell times and Hb-component mean values. In like manner, the SMTM and GA values for each adjacency matrix would be identical if $\sum_{i=1}^{N_{TS}}(U_k)_{ij}$ had the same value in all image voxels. Conversely, the difference between the GA and SMTM (TMSM) values of a given parameter is a measure of the parameter's coefficient of variation across the spatial (temporal) dimension.

13. Inclusion of Normalized Δ(Dwell Time) in Three-Flux Vector Amplitude Computation.

As previously reported in preliminary form [8], findings grossly similar to those of Fig. 5d and Supplementary Fig. 7 were seen with a reduced component fit (i.e., normalized fluxes (*t*′ scores) for two Hb-components: either ΔHbO_2Sat and ΔtotalHb, or ΔdeoxyHb and ΔoxyHb), but these differ from the results presented here with respect to important quantitative features such as disease impact on the

apparent "V_{max}" and "K_M" parameters. Extension to a three-component vector involved not a third Hb flux, but a qualitatively distinct network coefficient. The most straightforward choice involved the dwell-time parameters, for which a "flux" can computed as the difference between the post- and pre-transition values (Eq. (6)) for each transition type. Clear evidence that each of included fluxes have distinct contributions can be seen in Fig. 5a-c. Closer examination of the normalized flux values for the different transition types also provides evidence that the composite structured behavior arises from yet more elementary dependences that are themselves structured. We demonstrate this by rank-ordering the contributions of the three vector components to the net vector amplitude, for all Class 3-5 transition types. Orderly groupings among the transition types is observed (Table 2, Supplementary Note 14).

14. Supporting Information for sensitivity of vector amplitude to biomarkers of breast cancer.

In Supplementary Fig. 8 we show results of extending the L-B analysis of Fig. 5d to the question of whether the computed vector amplitudes exhibit sensitivity to the breast-cancer biomarkers ER (estrogen receptor) and Her2 (human epidermal growth factor receptor 2). This was accomplished by performing separate L-B computations for the ER(+) and ER(-) subjects (n = 13 and 5, respectively), and for the Her2(+) and Her2(-) subjects (n = 9 and 9). For the ER-status result plotted in Supplementary Fig. 8a, the regression lines have significantly different slopes ($p = 2.2\times10^{-8}$) and y-intercepts (p = 0.0081). In the corresponding Her2-status result, the regression lines have significantly different slopes ($p < 10^{-10}$) and x-intercepts (p = 0.0018). These observations of biomarker sensitivity in a result that considered all Classes 3-5 transition types was an impetus to the subsequent analyses to investigate the impact of vector-component rank order on the magnitude of the sensitivity.

Results in Table 2 demonstrate that the vector amplitudes are sensitive to the presence or absence of a particular biomarker. It is also seen that the biomarker sensitivity differs among the rank-order groupings (Supplementary Note 15), lending support to the suggestion that the clearly non-random patterning in Supplementary Fig. 14 is evidence of underlying structured dependences.

15. Transition-Type Rank Ordering by Relative Contributions of the Vector Components.

Presented in Table 2 is evidence of a structured dependence between net vector amplitude and the relative magnitudes of the contributions of the three components of the vector. Each transition type in Classes 3-5 (n = 50) was assigned a one-digit code determined by the rank-order of the t'-scores for the three network

coefficients. Plotted in Supplementary Fig. 14 are the rank-order codes for the Class 3-5 transition types, for the L-breast group-mean vector amplitudes. A non-random pattern of rank-orderings is observed (in which only four of the six theoretically possible rank-orderings are seen to occur: 1 = $|t'(\Delta \text{totalHb})|>|t'(\Delta \text{HbO}_2\text{Sat})|>|t'(\Delta(\text{dwell time}))|$, 2 = $|t'(\Delta \text{totalHb})|>|t'(\Delta(\text{dwell time}))|>|t'(\Delta \text{HbO}_2\text{Sat})|$, 3 = $|t'(\Delta \text{HbO}_2\text{Sat})|>|t'(\Delta \text{totalHb})|>|t'(\Delta(\text{dwell time}))|$, 4 = $|t'(\Delta \text{HbO}_2\text{Sat})|>|t'(\Delta(\text{dwell time}))|>|t'(\Delta \text{totalHb})|$), suggesting the possible existence of additional structured behaviors determining that pattern. While there is variability among patterns for different breast groups and between individuals, a result qualitatively similar to the L-group colormap is found in all cases.

The evidence of sensitivity to “hidden” enzyme-like behavior that invariably arises from a composite of actions, and the form of the disease sensitivity seen, strongly suggests that this composite is dominated by a specific enzymatic type. For example, it is recognized that pulsatile behavior of the vascular bed is modulated by vascular smooth muscle that itself is principally influenced by the enzyme soluble guanylate cyclase [9]. Strongly modulating the latter is nitric oxide, whose levels are increased in breast cancer [10] and is known to selectively enhance the apparent V_{max} of this enzyme [11], which is the behavior seen.

16. Method validation—Demonstration that applied mathematical treatments do not replicate experimental findings.

As recognized, a necessary validation step is to rule out the hypothesis that findings such as those presented in Results are inevitable consequences of applying the considered sequences of mathematical operations to time-series data. Here we have conducted this evaluation with a focus on four distinct feature behaviors seen in experimental measures: i) adjacency matrix patterning (Fig. 2), ii) its underlying sensitivity to ΔtotalHb (Supplementary Fig. 3), iii) occurrence of hyperbolic trends (Fig. 3c-d), iv) distinctive linear trends seen in L-B plots (Fig. 5d). We performed the validation by applying the same operations to simulated time-series data consisting of: A) random (Gaussian) noise, B) perfectly periodic (sinusoidal) continuous and smooth functions, or C) surrogates of real-data time series, generated by applying a phase randomization process [12]. The random-data study considers whether the reported findings necessarily result from treating correlated noise in the described manner, while the sinusoidal-data study looks at whether they necessarily result from applying those operations to periodic functions.

The surrogate-data study evaluates the impact on computed network coefficients of distorting the short-term dynamics information in the original time-series data.

For the noise and sinusoidal-data cases, the simulated data were tuned to match several basic properties of the real image data: baseline mean values of oxyHb and deoxyHb, mean amplitudes (standard deviations, in the gaussian noise case) of oxyHb and deoxyHb temporal variations about their respective mean values, and correlation between the simulated ΔoxyHb and ΔdeoxyHb time series. For the noise-data computations, it was empirically found that 2.5×10^6 points per simulated time series gave excellent repeatability. For the sinusoidal-data computations, where repeatability is not an issue, the simulated time series has the same length (i.e., 600 time frames) as that of the data used in generating Fig. 1b-c, Supplementary Fig. 7, and Supplementary Fig. 9.

A. Noise-based Computations and Findings.

To distinguish simulated data from real measurements, for the former we use 'dxy', exc', 'oxy', 'sat' and 'tot' to denote the deoxyHb, HbO_2Exc, oxyHb HbO_2Sat and totalHb components, respectively. Simulated time series are denoted by appending 'AC' to a three-letter Hb-component label, while the 'DC' suffix is used to indicate modeled baseline mean values. Details of the simulation performed to generate the findings shown in Supplementary Figs. 15-19 were:

1. oxyDC = 5.1×10^{-5} M, dxyDC = 9×10^{-6} M. These lead to baseline mean values 6×10^{-5} M for totDC and and 85% for satDC, respectively, in agreement with parameter settings used for image reconstruction [13].
2. Two sequences, x and y, of 2.5×10^6 pseudorandom numbers are generated, using the MATLAB 'randn' function with the default mean = 0, standard deviation (SD) = 1 settings.
3. The correlation-value target between oxyAC and dxyAC time series is set at r_{DO} = -0.4. Accordingly, a third pseudorandom sequence, z, is generated by computing $z = x \cdot r_{DO} + y \cdot \sqrt{1-r_{DO}^2}$ [14]; z has the same length and the same theoretical mean as x and y.
4. oxyAC = (0.02·oxyDC)·x; dxyAC = (0.01·oxyDC)·z. Simulated values for the remaining components are computed via the same formulas as applied to experimental data: totAC = dxyAC+oxyAC, excAC = dxyAC–oxyAC, satAC = 100·(oxyDC+oxyAC)/[(oxyDC+oxyAC) + (dxyDC+dxyDC)].

5. Values for the various network adjacency matrices were computed using the formulas in "Methods. Quantification of inter-state transition coefficients" with the simulated Hb-component time series as the input.

Plotted in Supplementary Fig. 15 are noise-derived adjacency matrices for the (a) transition probability (P) and (b) pre-transition dwell time ($\tau^{(1)}$) network parameters. Comparison of these to their measurement-data counterparts in Fig. 2a and Fig. 2b, respectively, shows that while some of the gross features of the biological adjacency matrices are present in the noise-based counterparts (this is not unexpected, given the effort to match principal elements of the measurement data), physiology-related properties of the former, such as dependence on the algebraic sign of ΔtotalHb, are absent in the latter. Plotted in Supplementary Fig. 16 are the ratios of adjacency-matrix values (Supplementary Note 7) for the noise-based P (Supplementary Fig. 16a) and $\tau^{(1)}$ (Supplementary Fig. 16b). The ratio-value patterns exhibit no asymmetry between algebraic sign-changing and sign-preserving transition types, or any suggestion of the types of structure found in their measurement-data counterparts (P – Supplementary Fig. 3a, $\tau^{(1)}$ – Supplementary Fig. 3b). The Supplementary Figs. 15-16 results thus demonstrate that the structured behaviors seen in measurement data-based adjacency matrices are not inevitable consequences of the operations performed in computing them.

Shown in Supplementary Fig. 17 is a simulated-noise counterpart to the "bicycle wheel" plot of Fig. 3a. In the Supplementary Fig. 17 plot of noise-based satAC pre-transition mean values vs. totAC pre-transition means, it is seen that the pattern of 100 data points grossly resembles its measurement-data counterpart in Results Fig. 3a, in that data points having the same color/fill labeling are found in the same State sector. However, in Supplementary Fig. 17 the points in each sector appear to lie atop each other, and zooming in on the grouped points reveals no consistent structure at any scale.

Shown in Supplementary Fig. 18 is a simulated-noise counterpart to the hyperbolic trends shown in Fig. 3d for experimental measures. In the Supplementary Fig. 18 plot of noise-based totAC flux vs. noise-based totAC post-transition mean values, it is seen that the pattern of 100 data points has some gross features—the ordering of groups of same-color points in the flux dimension, and the lower-left to upper-right trend in each same-color set—that resemble the measurement-data counterpart in Results Fig. 3d, but the Supplementary Fig. 18 plot lacks the highly structured co-dependences seen in the latter. In

particular, no evidence of hyperbolas (or any curvilinear trend) is observed. For example, the set of points circled in Supplementary Fig. 18 are the data for the 10 transition types having post-transition State 10, and here they are seen to lie on a straight line, in contrast to the hyperbolic co-dependence in the corresponding points in Fig. 3d.

In like manner, Supplementary Fig. 19 shows that the simulation-data L-B plot, obtained by substituting noise-based adjacency matrix values for their measurement-data counterparts, does not exhibit any dependence on transition probability of the sort seen in Fig. 5d.

Additional results obtained when a limited sweep across values of the input parameters was performed show that in all cases the primary findings of Fig. 2, Fig. 3c-d, Fig. 4, and Fig. 5d do not appear in the noise-substituted data. For example, lowering the value of satDC (by simultaneously decreasing oxyDC and increasing dxyDC, while holding their sum constant) produces a nearly uniform shrinkage of the pattern of data points in Supplementary Fig. 18 (i.e., a smaller distance from each data point to the origin), without altering its shape, its sequences of data-point colors and shapes, or the lack of hyperbolic (or other non-linear) co-dependences.

In contrast, one result from the model-parameter sweeps warrants mention here, because it suggests a biological interpretation for a detail seen in, e.g., Fig. 2b. Namely, as the correlation between oxyAC and dxyAC is increased, from negative values to zero and then to positive values, the relative magnitudes of noise-simulation dwell-time values for States 2 and 7 increase, while those for States 4 and 9 fall. This suggests that the observation of higher pre-transition dwell times in both pairs of States in Fig. 2b can be partially attributed to the expectation that the plotted group-mean result is a composite of data spanning a wide range of correlations between ΔoxyHb and ΔdeoxyHb.

Finally, it is recognized that the simulation model described here deliberately generates a crude approximation to the properties of the measurement data, and that refining model assumptions could produce simulation results that reproduce more features of findings reported in Results. As an example, increasing the autocorrelation time of the simulated time series could be expected to affect Supplementary Fig. 15a by increasing the probabilities of transitions from a State to itself (i.e., values on the main diagonal of the adjacency matrix) and decreasing the reciprocal-transition probabilities. However, any model

feature that improves the accuracy of the result will do so by increasing the similarity between the measured and simulated data. That is, simulation-based findings will more closely resemble those in Results to the extent that genuinely biology-determined properties of measurement data are incorporated into the simulation model.

B. Sinusoidal function-based Computations and Findings.

As an extension of method validation, we recognize that vascular pulsations produce quasi-sinusoidal behaviors. Here we show that plots equivalent to the **C** matrix shown in Supplementary Fig. 9 are not replicated by this class of phenomenology.

The same nomenclature (e.g., ‘oxy’ for the oxyHb hemodynamic-signal component, ‘DC’ for baseline mean values, and ‘AC’ for amplitudes of the temporal fluctuations), baseline mean values (e.g., oxyDC = 5.1×10^{-5} M), fluctuation amplitudes (e.g., $|\text{oxyAC}| \leq 0.02\cdot\text{oxyDC}$), and temporal correlation (i.e., r_{DO} = -0.4) were used here as in the noise-data computations. In comparison to the 5-step computation described in Supplementary Note 16.A, the only difference here is that Step 2 becomes:

2. Two 600-number sequences, $x = \cos(0.05t)$ and $y = \sin(0.05t)$, are generated, for $t = 0.25, 0.75, \ldots, 299.75$. The sinusoidal frequency is intentionally given an irrational-number value (i.e., $(40\pi)^{-1}$), to guarantee that distinct sets of function values will be sampled in every period.

We further note, in passing, that the Step-3 operation of computing z as a linear combination of x and y is equivalent to imposing a fixed phase difference of $\cos^{-1}(r_{DO})$ between x and z.

The **C** matrix computed from the sinusoidal-model data is shown in Supplementary Fig. 20. Its structure differs notably from that of Supplementary Fig. 9, in that only 20 of the 100 transition types are seen, and those occur in a specific, invariant sequence. Closer examination reveals that every observed transition type is either in Class 0 or Class 1; that is, they correspond to either a dwelling in the current State (e.g., 1→1, 2→2), or a transition from a State to one immediately adjacent to it in Fig. 1a (e.g., 1→2, 2→3). Owing to the fixed value of r_{DO}, transitions in the reverse direction (e.g., 2→1) do not occur.

It should be noted that some features of the Supplementary Fig. 20 plot—e.g., the occurrence of only a single transition at any given time—are a consequence of the simulation considering only one set of co-varying time series (i.e., a single "image voxel"). If multiple voxels having a distribution of sinusoidal phases or frequencies were modeled, then the transition counts could have values >1 and there could be more than one non-zero value at a specified time. However, non-zero count values still would be seen for only the 20 Class-0 and Class-1 transition types that have non-zero values in Supplementary Fig. 20.

C. Surrogate data-based Computations and Findings.

As a further extension of method validation, we recognize a theoretical possibility that reported Results might be determined by features of the volumetric-image time series that are of biological origin (e.g., their frequency-dependent power-spectral densities) but unrelated to the short-term behaviors that the fr-OPN method purports to capture. Details of the calculation performed to evaluate this conjecture were:

1. For a selected subject and breast (i.e., the same as used to generate the Fig. 1b result), discrete Fourier transforms (FT) were computed for the ΔdeoxyHb and ΔoxyHb time series for each image voxel. The number of FT frequencies was set at $N_t' = 2^{\lfloor \log_2 N_t \rfloor}$, where N_t is the number of image time frames and $\lfloor X \rfloor$ (i.e., the floor function) is the largest integer ≤ X.
2. A random permutation of the integers 1 through N_t' was generated for each image voxel, and the FT phases for both ΔdeoxyHb and ΔoxyHb were arranged in the permuted order. Thus the power spectrum for every time series was unperturbed, while the phases were randomized.
3. Values for the remaining Hb-signal components were computed via the same formulas as applied to the unperturbed experimental data.
4. Values for the network adjacency matrices were computed as described in "Methods. Quantification of inter-state transition coefficients," with the surrogate Hb-component time series as the input.
5. Steps 2-4 were repeated for a total of 100 iterations, thereby permitting construction of an empirical distribution of surrogate-data values of every network coefficient for each transition type.

The statistical significance of each experimental-data network-coefficient value was evaluated via a t-test, on the null hypothesis that the unperturbed value is the true mean of the surrogate-data distribution. Test results are reported in Table S3, for the four network coefficients used in generating a L-B plot (e.g., Fig. 5d): the three fluxes that are vectorially combined, and the transition probability. In every case it is

seen that, for a large majority of transition types (i.e., 93–99 out of 100), the difference between the original-data adjacency-matrix values and the corresponding surrogate-data mean is highly significant (i.e., $<10^{-4}$). This shows that the reported findings are determined by features of the biological time-series data that are of higher order than its frequency-amplitude structure.

D. Impact of Tomographic Reconstruction on Detection of Enzyme-Like Behaviors.

We have separately explored the potential impact of tomographic reconstruction on the character of findings reported here. Being a spatially non-linear weighting scheme, one effect of reconstruction is to reduce expected spatial biases that otherwise are inherently present in the native signal as a consequence of light diffusion. Explicitly evaluated has been the individual-subject result reported in Supplementary Fig. 7. Computed was an equivalent plot, but input measures from the native signal, which were not subject to 3D tomography computations, were used prior to applying the fr-OPN scheme and evaluating the plotted ordinate and abscissa quantities. Regression-line parameters were: tumor breast $V_{max} = 8.3 \pm 1.6$, $K_M = 0.25 \pm 0.05$, $r = 0.89$; unaffected breast $V_{max} = 1.7 \pm 0.4$, $K_M = 0.14 \pm 0.04$, $r = 0.81$. Comparison to Supplementary Fig. 7 reveals that implementation of 3D tomography does not qualitatively impact the findings reported.

17. Comparison of fr-OPN to OPN Network Descriptions.

The OPN approach is a member of the transitions-networks category of strategies for converting times series to networks [15]. In common with all methods in this class, a network is constructed by assigning a symbolic encoding to the time-series data, then mapping the symbols into network nodes and the transition function between the symbols into network edges [15].

The original, univariate OPN implementation operates on sets of *N* data values that are separated by a constant interval of τ intervening data values (i.e., $\{x(t_0), x(t_0+\tau), x(t_0+2\tau), \ldots, x(t_0+(N-1)\tau)\}$, where $x(t)$ is the value of the time-varying function *x* at time *t*, and *N*, τ and t_0 are chosen as appropriate for the specific context of the phenomenon under consideration). The ordinality attribute of the indicated sequence is abstracted by retaining the "greater than" and "less than" relations among the data values while discarding all other numerical information; thus, the sequences {1, 2, 3}, {1, 1.2, 30} and $\{1, 10^3, 10^6\}$ are ordinally identical even though they have markedly different intervals between successive data values. Each permutation of the data-value order information is defined as a state or node of the network

[15]. Thus, a univariate OPN can have as many as $N!$ nodes, although dependences within the process that generates the time series may have the effect of reducing the number actually observed. Likewise, if, for example, the probabilities for transitions among pairs of OPN states are taken as network edge weights, then dependences in the generative process can have the effect of enhancing some while attenuating, or even eliminating, others.

Many of the attributes of univariate OPN networks carry over to the multivariate case, while others undergo modifications, as will be noted. Conversion of multivariate time-series data (i.e., $a(t)$, $b(t)$, $c(t)$, etc.) to an OPN network is performed by considering pairs of data values for each time-varying function (i.e., $\{a(t_0), a(t_0+\tau); b(t_0), b(t_0+\tau); c(t_0), c(t_0+\tau); \ldots\}$). A value of 1 (or '+') is assigned to any pair whose value is greater at time $t_0+\tau$ than at time t_0, and a 0 (or '-') to any pair whose value decreases from t_0 to $t_0+\tau$ [15,16]. Each permutation of 1s and 0s is defined as a network node. Thus, if a multivariate data comprises n time series, the corresponding OPN can have as many as 2^n nodes. That is, the (exponential) dependence of maximum multivariate network size on n is different from the (factorial) dependence of maximum univariate network size on N. However, common to the uni- and multivariate cases is the possibility that data dependences can lead to the number of observed nodes being less than the theoretical maximum.

The procedure for generating a multivariate OPN network as considered by [16] can be applied to the set of five Hb signal-component time series. When we do so, we find that, just as for the States-transitions network described in this report, dependences among the components lower the observed number of OPN states from 32 (i.e., 2^5) to 10. However, the two networks are not equivalent, and as shown below the OPN approach of [16] in more than one way convolves information that is kept separate in the State-transitions method (fr-OPN). Importantly, in at least one way the noted convolution involves mixing types of information that we regard as differing in their biological significance. In contrast, the conventional OPN approach was developed in contexts more remote from the biological phenomenology that is considered here. We illustrate the preceding claims by presenting a more detailed comparison of findings from the two time-series-to-network strategies, when both are applied to image time series for the five Hb-signal components, for the non-cancer subjects in the breast imaging study.

Examination of Fig. 1a shows that in the network described in this report, a voxel is in State 1 in the ith time frame if:

$$\Delta\text{deoxyHb}_i < 0, \quad \Delta\text{HbO}_2\text{Exc}_i < 0, \quad \Delta\text{oxyHb}_i < 0, \quad \Delta\text{HbO}_2\text{Sat}_i > 0, \quad \Delta\text{totalHb}_i < 0. \tag{S17.6}$$

The analogous criteria for OPN state 1 (using $\tau = 1$, to match the time intervals of OPN states with those of our network's transitions) are:

$$\begin{gathered}\Delta\text{deoxyHb}_i < \Delta\text{deoxyHb}_{i-1}, \quad \Delta\text{HbO}_2\text{Exc}_i < \Delta\text{HbO}_2\text{Exc}_{i-1}, \quad \Delta\text{oxyHb}_i < \Delta\text{oxyHb}_{i-1}, \\ \Delta\text{HbO}_2\text{Sat}_i > \Delta\text{HbO}_2\text{Sat}_{i-1}, \quad \Delta\text{totalHb}_i < \Delta\text{totalHb}_{i-1}.\end{gathered} \tag{S17.7}$$

Note that the directions of the inequalities in Eq. (S17.7) match those in Eq. (S17.6), while the zeros on the right-hand sides of the Eq. (S17.6) inequalities are replaced by data values for the $(i - 1)^{th}$ time frame (more generally, for the $(i - \tau)^{th}$ frame) in Eq. (S17.7). The same correspondence holds for the remaining nine states.

While the State definitions that we employ assume that there is biological significance to whether an instantaneous component value is above or below the temporal mean, the OPN states definitions discard precisely that information. This can be seen by noting that, for example, the $\Delta\text{HbO}_2\text{Sat}_i > \Delta\text{HbO}_2\text{Sat}_{i-1}$ inequality in Eq. (S17.7) does not draw any distinction among transitions in which $\Delta\text{HbO}_2\text{Sat}$ increases from a negative value to a positive one, from a positive value to a more-positive one, or from a negative value to a less-negative one. Rather, and in contrast to the network methodology described in this report, the three cases are treated as equivalent. In consequence, the OPN states, and the transitions among them, may fail to capture biologically important events.

As the state definitions for the multivariate OPN network entail comparing Hb signal-component values for two time frames, those states correspond more closely to the transition types of the States-transitions network outlined here than they do to its States. However, that correspondence cannot be one-to-one, since there are 100 transition types and only 10 OPN states. To determine exactly how the two approaches are related, we computed the specific OPN state corresponding to every transition, for the breast-image time series of the 45 non-cancer subjects. As shown in Supplementary Fig. 21, each OPN state is a non-uniform mixture of contributions from 39 distinct transition types. Here again, to the extent that our biology-based rationale for defining the States as we do is valid, then different transition types

contributing to a given OPN state can have different physiological significance, resulting in a convolution of information in the OPN findings.

Inspection of Supplementary Fig. 21 shows that there are overlaps between the sets of transition types that contribute to each OPN state; in particular, states j and j±1 have 30 transition types in common, decreasing to 10 in common for states j and j±5. The observed phenomenology suggests that physiological information pertaining to a given transition type may be spread across multiple states. This complementary aspect of the distribution of transition types over OPN states is summarized in Supplementary Fig. 22. Plotted is the number of OPN states that include transitions of each of the 100 types. It is seen that only the Class-5 transition types have one-to-one mappings into unique OPN states, while types in the other transition Classes are distributed over 2, 3, 4, 5 or 10 states. Also worth noting is the perfect Spearman correlation between the Supplementary Fig. 22 result and the transition-Class structure (e.g., Fig. 4 inset), with, for example, every Class-1(2) transition type mapping to 5(4) states. This phenomenon adds support to the premise that the Classes constitute functionally distinct transition-type groupings.

Also evident by inspection of Supplementary Figs. 21-22 is that every OPN state contains contributions from all 10 Class-0 transition types. These are the types that are self-loop transitions in the States-transitions network, where they represent fluctuations in signal levels that are too small to qualify as changes from one State (i.e., one gross physiological condition) to a different one. To the extent that this is a valid premise, it would follow that the OPN states would be mixtures of events that span a wide range of importance. The magnitudes of the Class-0 contributions to the OPN states are plotted in Supplementary Fig. 23. It is seen that they account for at least one-fifth (19.3%), to more than one-third (37.2%), of the individual transitions for each state.

We acknowledge here that counterparts for all of the adjacency matrices (i.e., network edge weights) considered in this report can be defined and computed for a multivariate OPN network. Indeed, analysis of transition probabilities among the states has yielded informative findings for the considered model systems [16]. But if, as suggested here, the multivariate OPN approach is applied to a problem area for which it is not well-motivated, then it could be expected that the issues identified here for the OPN states would carry over to the other network parameters.

Finally, strategies of mapping time series to a network have been considered in the form of visibility graphs [17], which have been applied to physiological measures [18-20]. This approach does not generate coefficients based on the temporal evolution of the signal. Instead, interactions between data values are permitted for any time lag, leading to idiosyncratic convolutions of temporal information. In contrast, our sampling of time-evolving signals preserves the opportunity to explore intact behaviors, some of which may reflect actions of hidden factors. A further attribute of the visibility-graph computation is that the network size is determined by the length of the time series. In our formalism the dimensions of adjacency matrices are independent of time-series length (and the number of spatial elements), which facilitates comparisons of results from different data sets.

Supplementary Figures

Supplementary Figure 1

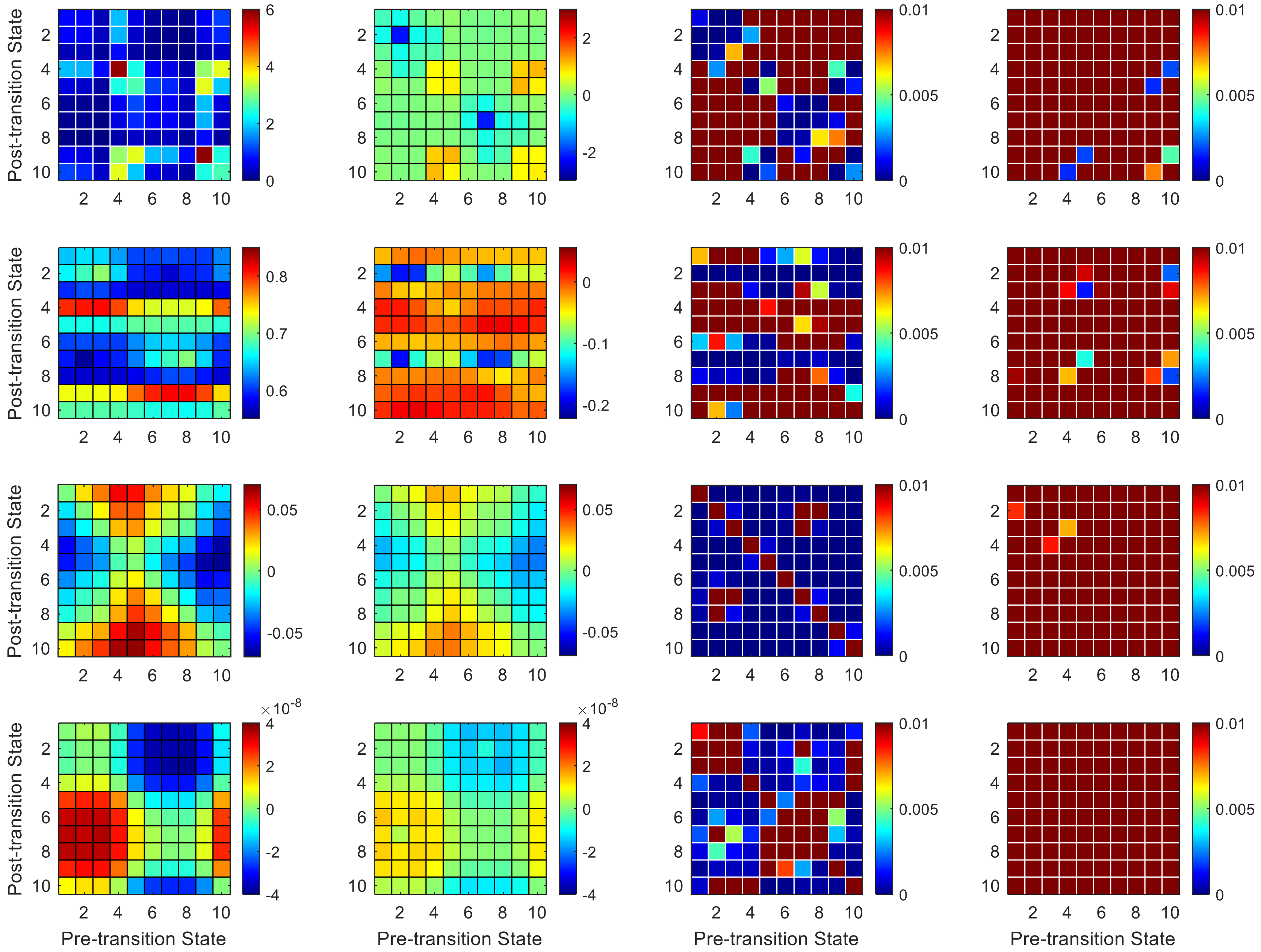

Supplementary Figure 1. Disease Sensitivities of Individual Network Coefficients. Left-to-right in the four columns of each panel: T group-mean coefficient value, T-U difference, and unequal-variance t-test *p*-values for the null hypotheses T – U = 0 and L – R = 0. Results are shown for (top to bottom rows): State transition probability (units are %), post-transition dwell time (seconds), and flux values for ΔHbO_2Sat (%) and ΔtotalHb (M).

Supplementary Figure 2

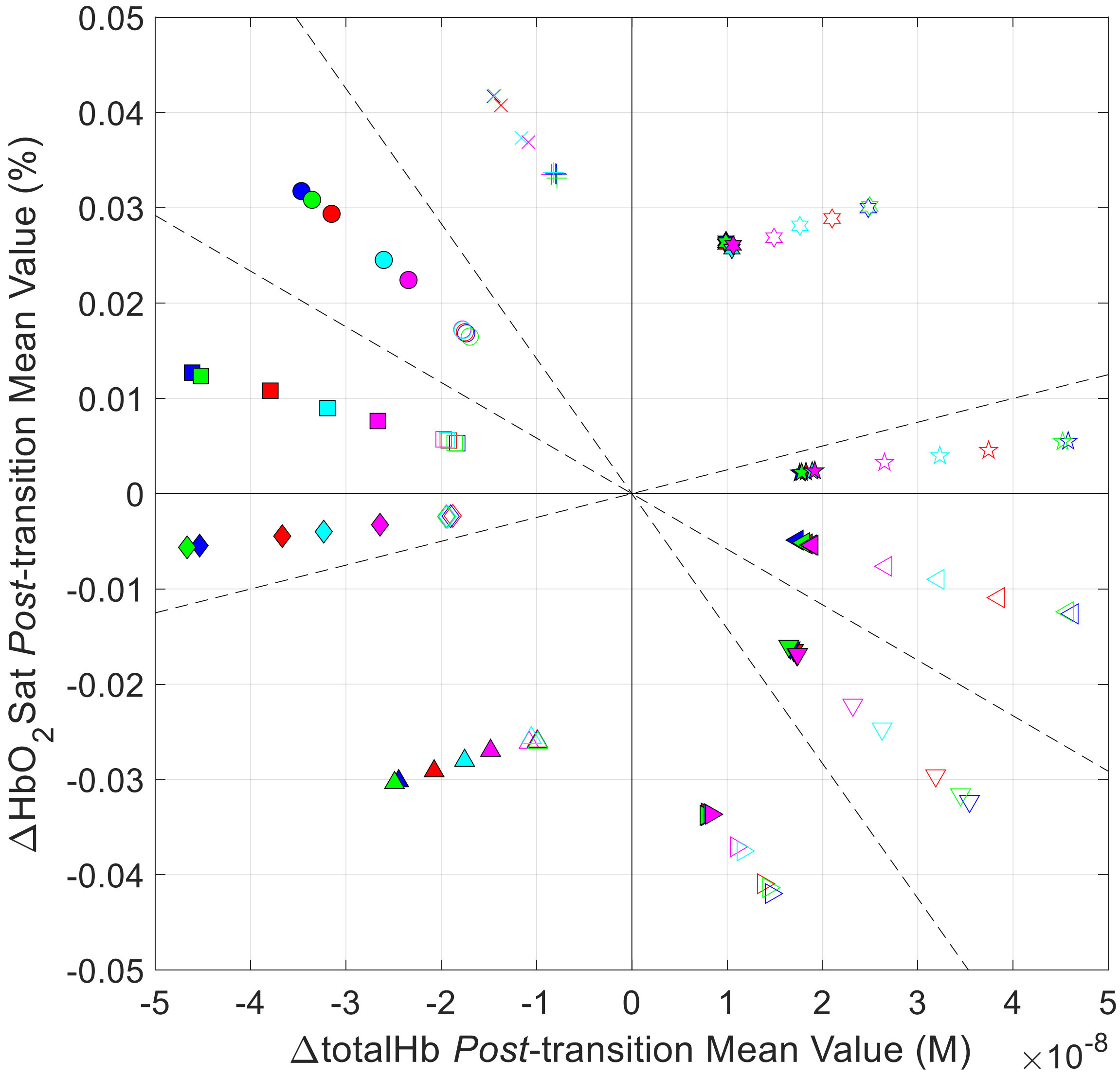

Supplementary Figure 2. Additional Features of Co-dependence Plots for Hb-Component Amplitudes. Shown is a plot of ΔHbO_2Sat post-transition means vs. ΔtotalHb post-transition means (cf. Fig. 3a). Group-mean data values for the tumor-bearing breast are shown. Each distinct symbol shape denotes a different one of the 10 post-transition States, and each combination of symbol color and open-or-filled symbol denotes a different one of the 10 pre-transition States (Fig. 3b). Dashed lines are the null curves for the other Hb components (Fig. 1a,c).

Supplementary Figure 3

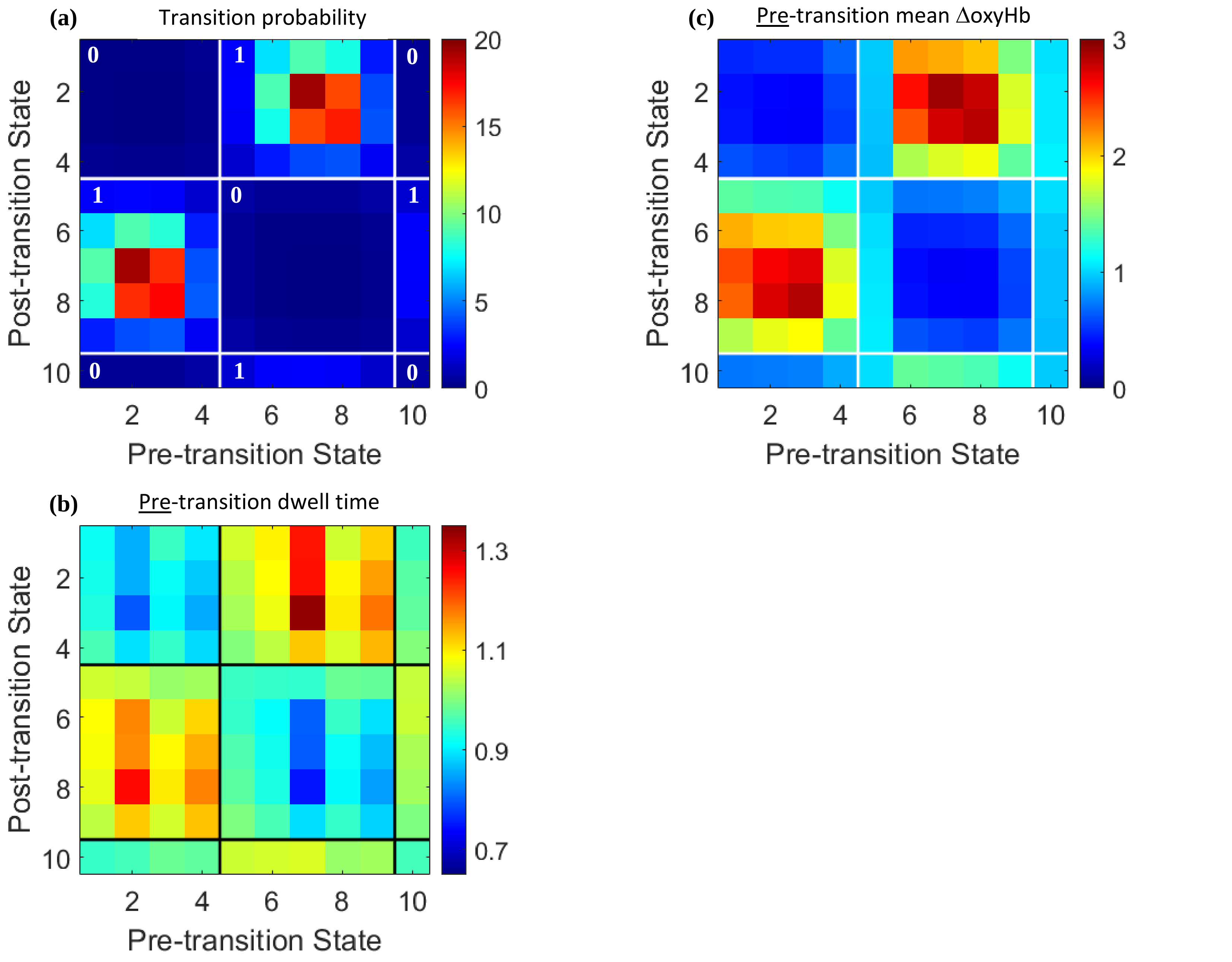

Supplementary Figure 3. Dependence of adjacency-matrix values on transition-related changes in Hb-component algebraic signs. Plotted in each matrix element is the ratio of adjacency-matrix values for two transition types, which are chosen so that every signal component undergoes a sign change in one transition type and is unchanged in the other type. Input data are averages of left- and right-breast group means for non-cancer subjects. Parameters considered are: **a**, transition probability; **b**, pre-transition dwell time; **c**, pre-transition level of ΔoxyHb. Thin horizontal and vertical lines separate the 10×10 matrices into transition types for which the algebraic sign of ΔtotalHb does (‘1’) and does not (‘0’) change.

Supplementary Figure 4

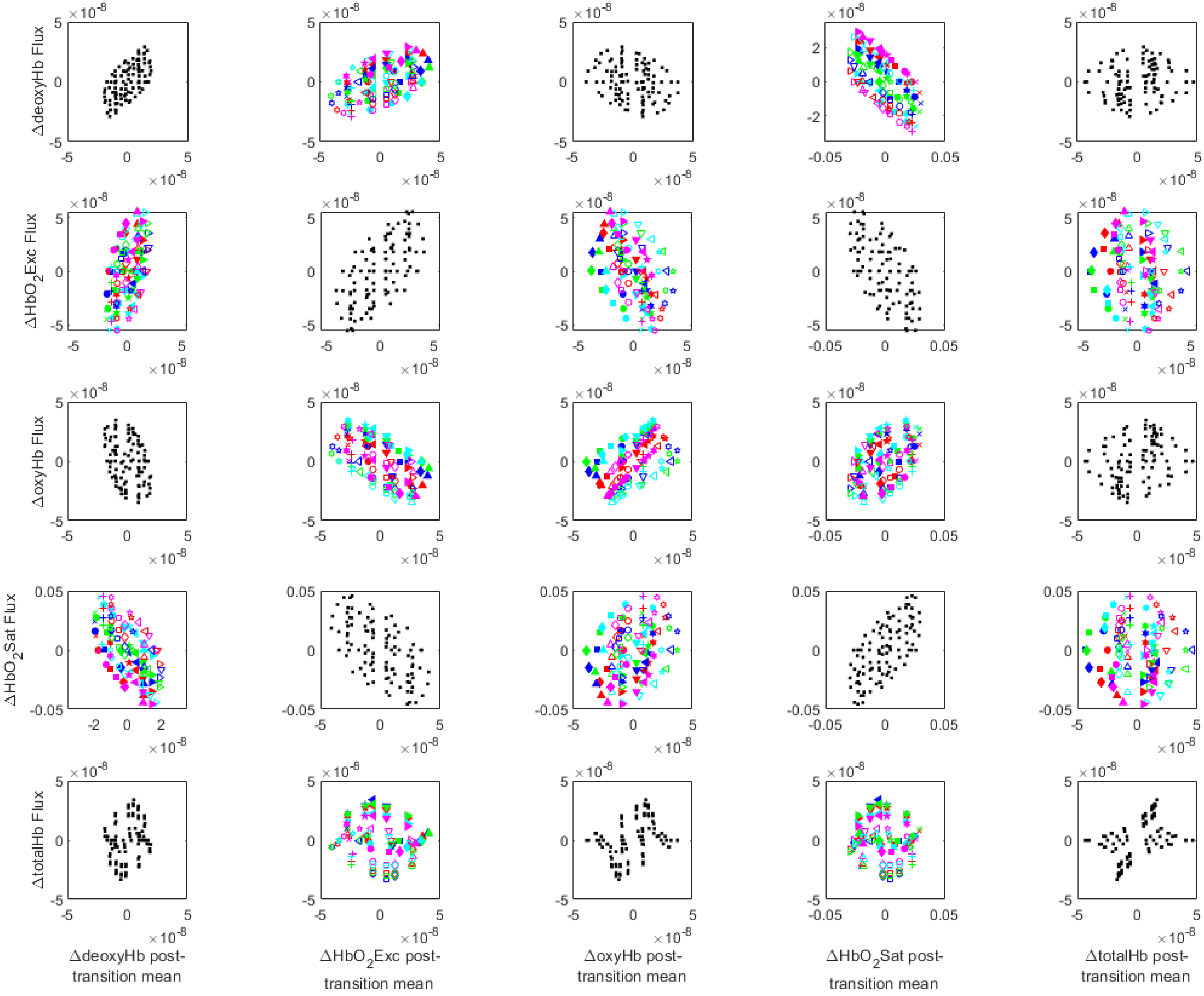

Supplementary Figure 4. Plots of co-dependences between flux and post-transition mean values, for all 25 two-component pairings. Input data are averages of left- and right-breast group means for non-cancer subjects. Vertical-axis labels in column-1, row-*i* apply to all five panels in the ith row; horizontal-axis labels in row-5, column-*j* apply to all five panels in the jth column. Color-fill-shape-labeling (Fig. 3b) is applied to the markers in 13 of the 25 subplots, while in the remaining 12 the markers are black dots (Supplementary Note 8).

Supplementary Figure 5a-b

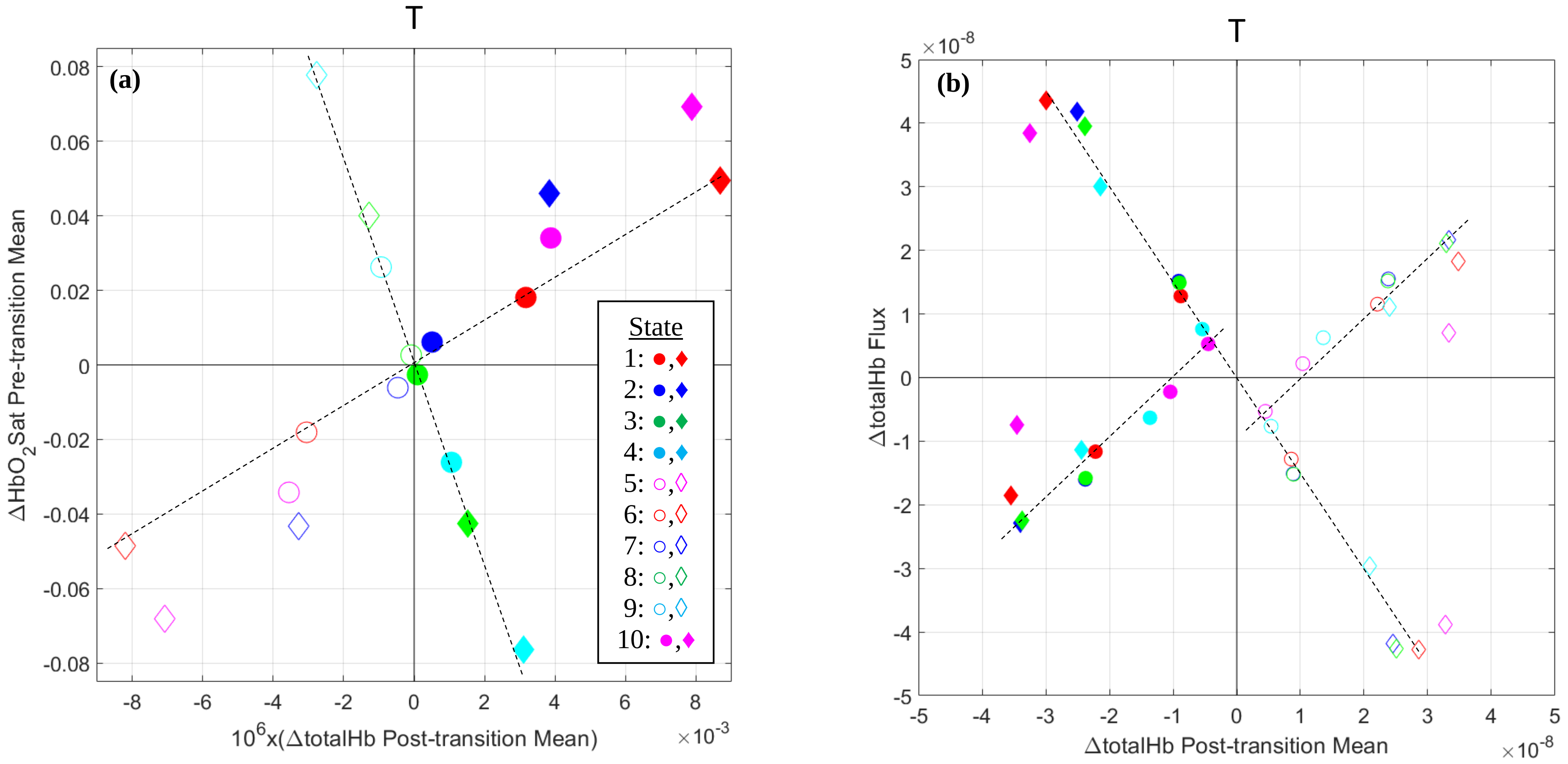

Supplementary Figure 5c-d

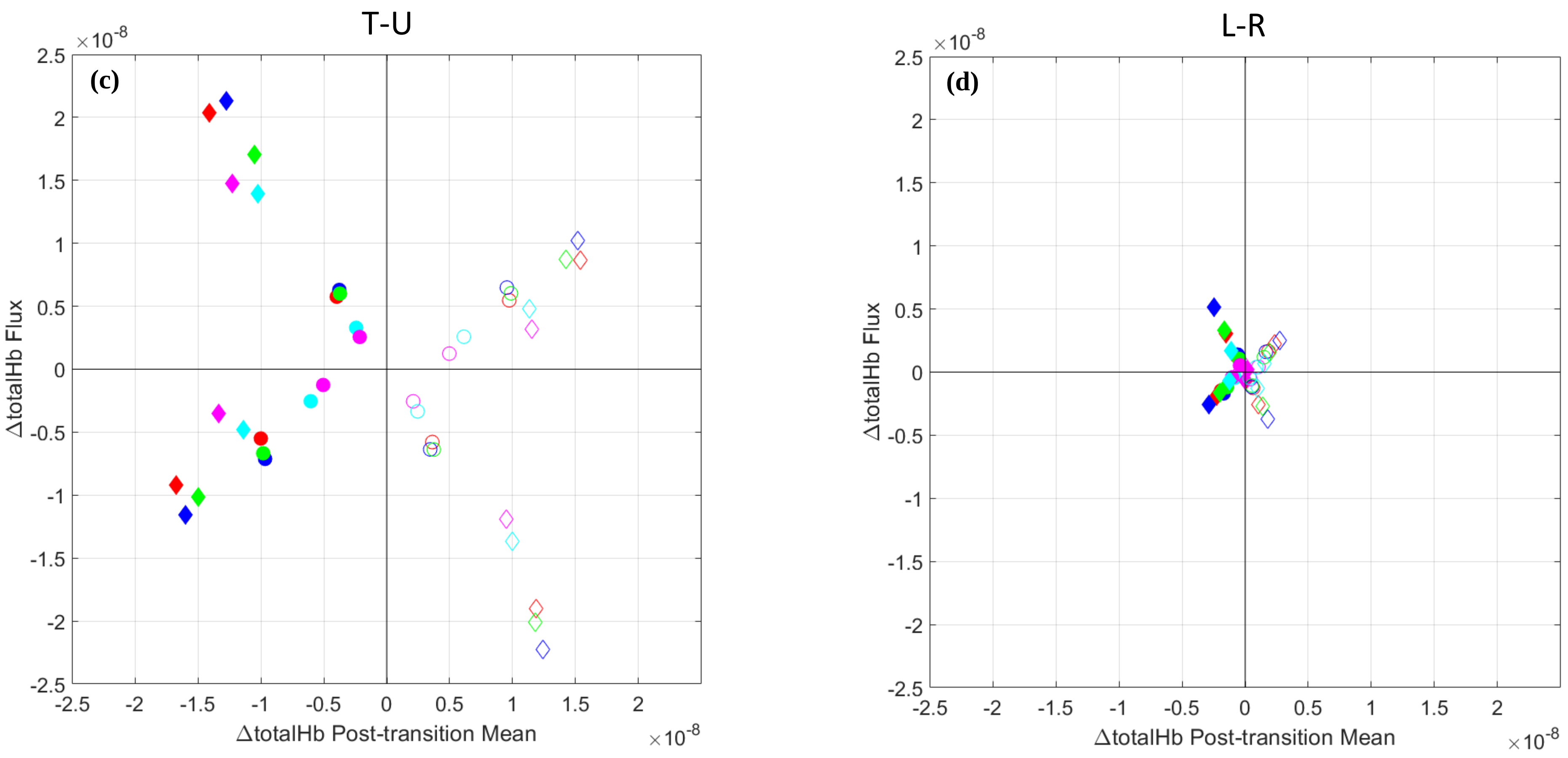

Supplementary Figure 5. Plots of the foci and vertices of Fig. 3c-d hyperbolic co-dependences. **a**, Plotted points are foci (diamonds) and vertices (circles) of the hyperbolas in the Fig. 3c plot of ΔHbO_2Sat pre-transition mean vs. ΔtotalHb post-transition mean, T-breast group. **b**, Points are foci and vertices of the hyperbolas in the Fig. 3d plot of ΔtotalHb flux vs. ΔtotalHb post-transition mean, T-breast group. Dotted lines in (**a**) and (**b**) are manually overlaid to highlight the indicated trends and are not fitted regression lines. **c-d**, 2D differences between the focus and vertex coordinates for the T and U groups (**c**), and between the L and R groups (**d**), for ΔtotalHb flux vs. ΔtotalHb post-transition mean.

Supplementary Figure 6

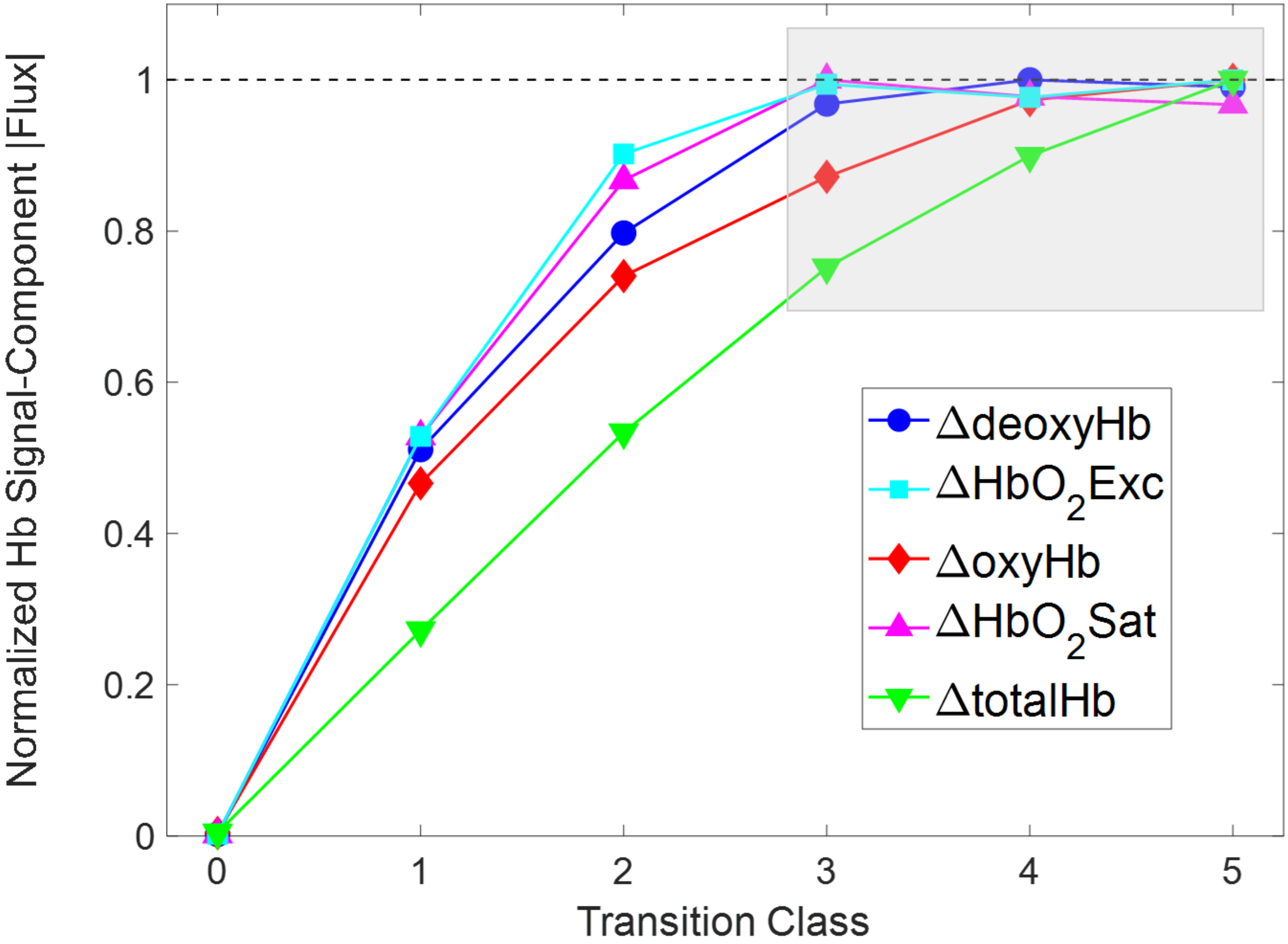

Supplementary Figure 6. Transition-Class Trends in Hb-Component Flux Magnitudes. Plots of the absolute value of transition flux for all transition types in each transition Class, for each of the five Hb-signal components. To permit plotting of all curves on a single set of axes, the |flux| values for each component were normalized to that component's maximum |flux| value. Gray shading indicates the transition Classes used in computation of results shown in Fig. 5d. Shown are data for the T breast group, with fluxes computed using the GA averaging method (Eq. (10)).

Supplementary Figure 7

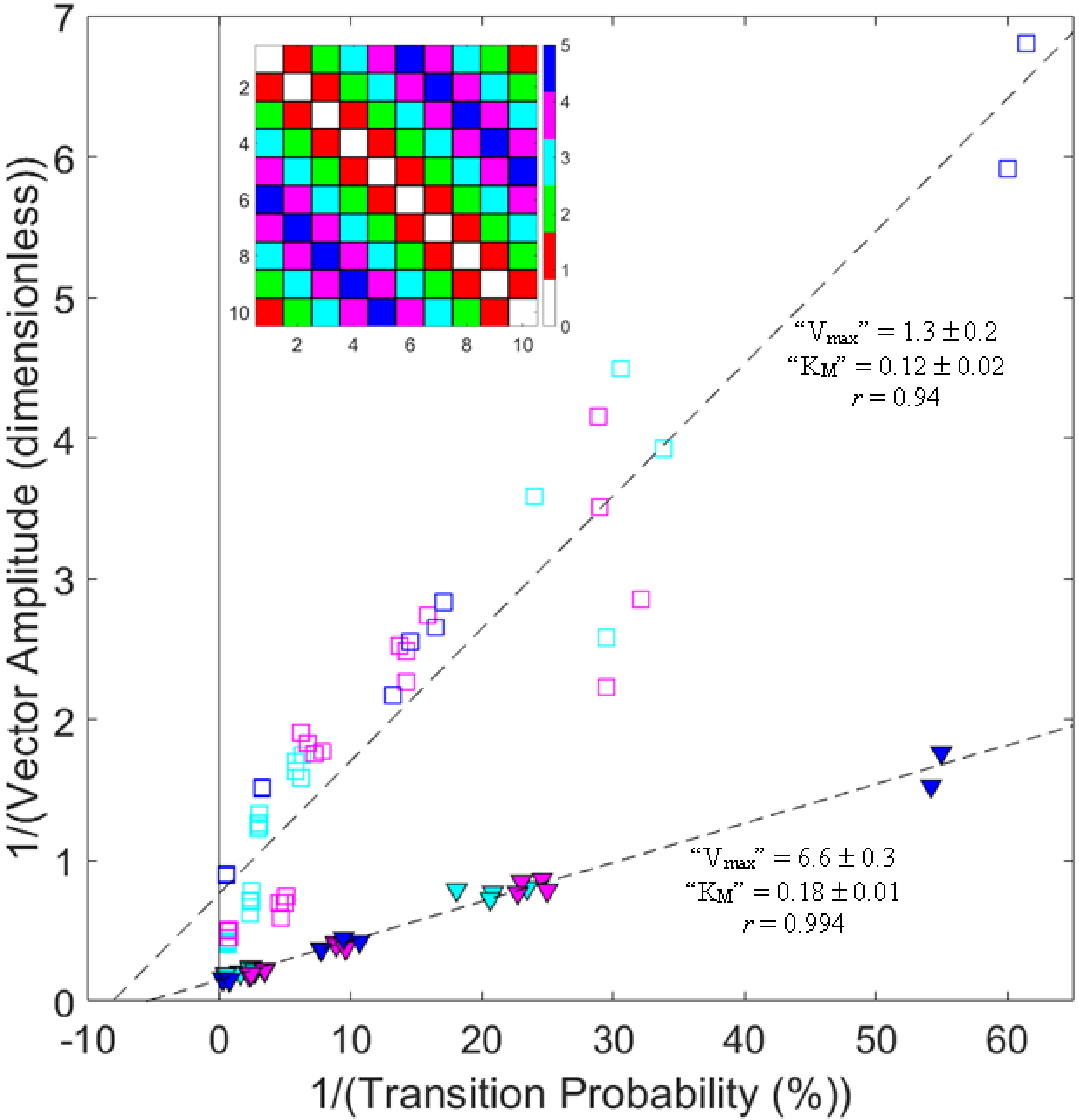

Supplementary Figure 7. Enzyme-Like Behavior in Composite Network Coefficient Measures for an Individual Subject. Transition Class-labeled plot of 1/(vector amplitude) vs. 1/(transition probability) for T- and U-breast data (filled triangle and unfilled square markers, respectively) for a selected individual subject, for Classes 3-5 (see inset 2D array for Class-label colors); dashed (T) and dash-dot (U) lines are the linear fits to the full set (n = 50) of Classes 3-5 points. The three network coefficients used as vector components (Eq. (11)) are ΔtotalHb flux, ΔHbO_2Sat flux, and the difference between post- and pre-transition dwell times.

Supplementary Figure 8

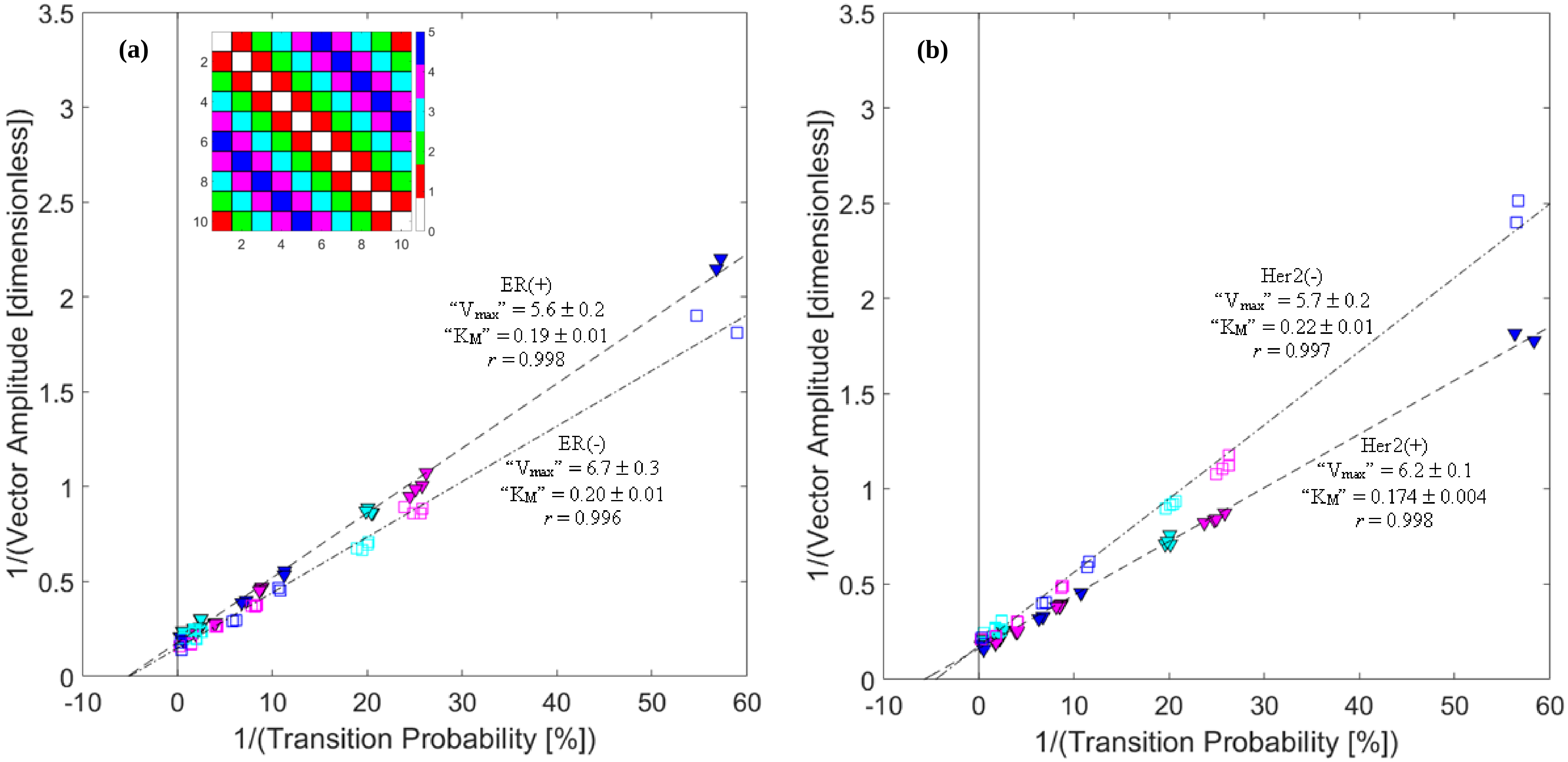

Supplementary Figure 8. Tumor Biomarker-Sensitive L-B Plot. **a**, Transition Class-labeled plot of 1/(vector amplitude) vs. 1/(transition probability) for the ER(+) (n = 13) and ER(-) (n = 5) subsets of the T-breast data (filled triangle and unfilled square markers, respectively). **b**, Corresponding 1/(vector amplitude) vs. 1/(transition probability) plot for the Her2(+) (n = 9) and Her2(-) (n = 9) subsets of the T-breast data. Plotted in all cases are the 50 Classes 3-5 data values (see inset 2D array in (**a**) for Class-label colors); dashed (biomarker-(+)) and dash-dot (biomarker-(-)) lines are the corresponding linear fits. The three network coefficients used as vector components (Eq. (11)) are ΔtotalHb flux, ΔHbO_2Sat flux, and the difference between post- and pre-transition dwell times.

Supplementary Figure 9

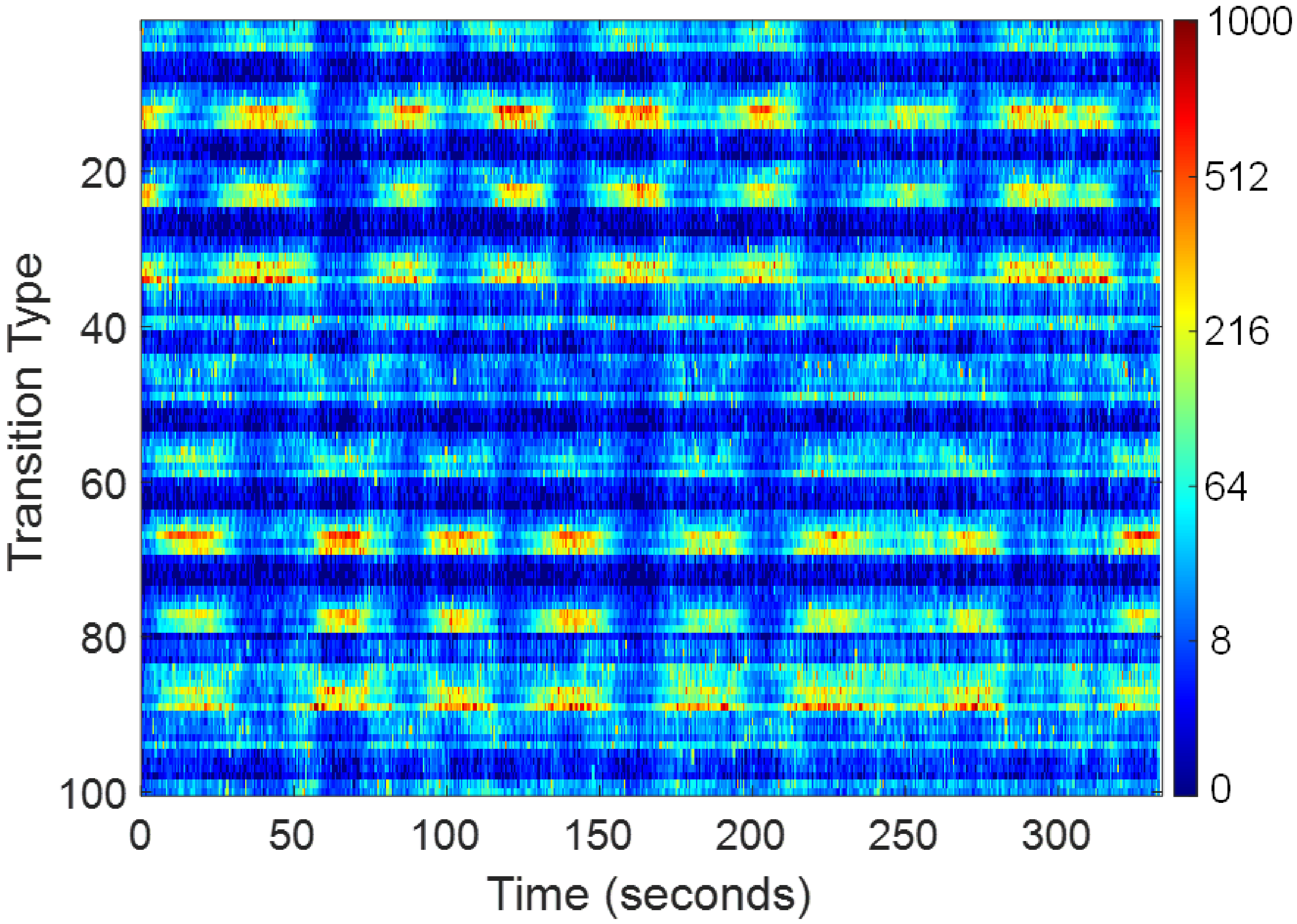

Supplementary Figure 9. Transition count matrix (Eq. (3)) for an exemplary subject. Shown is the time- and transition type-dependent transition count **C** for the unaffected breast of a breast-cancer subject. Row dimension is transition type as defined in Eq. (1), column dimension is the measurement time step (in units of seconds), and the magnitude of the transition count is indicated by the color (a nonlinear (third-root) scale is used, to facilitate visualization of the full range of counts).

Supplementary Figure 10

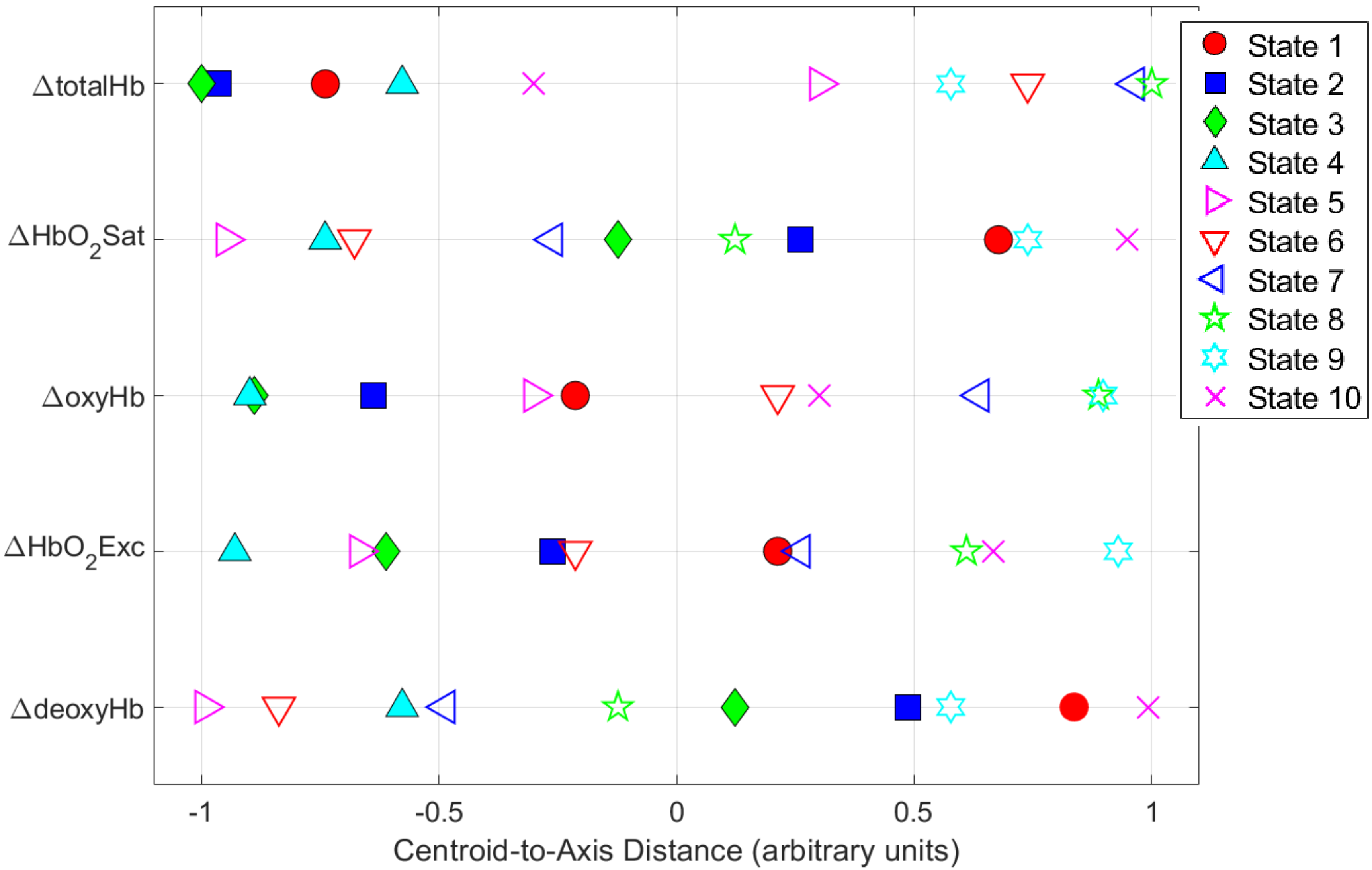

Supplementary Figure 10. Uniform-disk model mean value (y-axis) of each Hb-signal component for each State (x-axis). The ΔtotalHb sequence (top row) qualitatively matches the order of data values along each "spoke" in a plot such as Fig. 3a.

Supplementary Figure 11

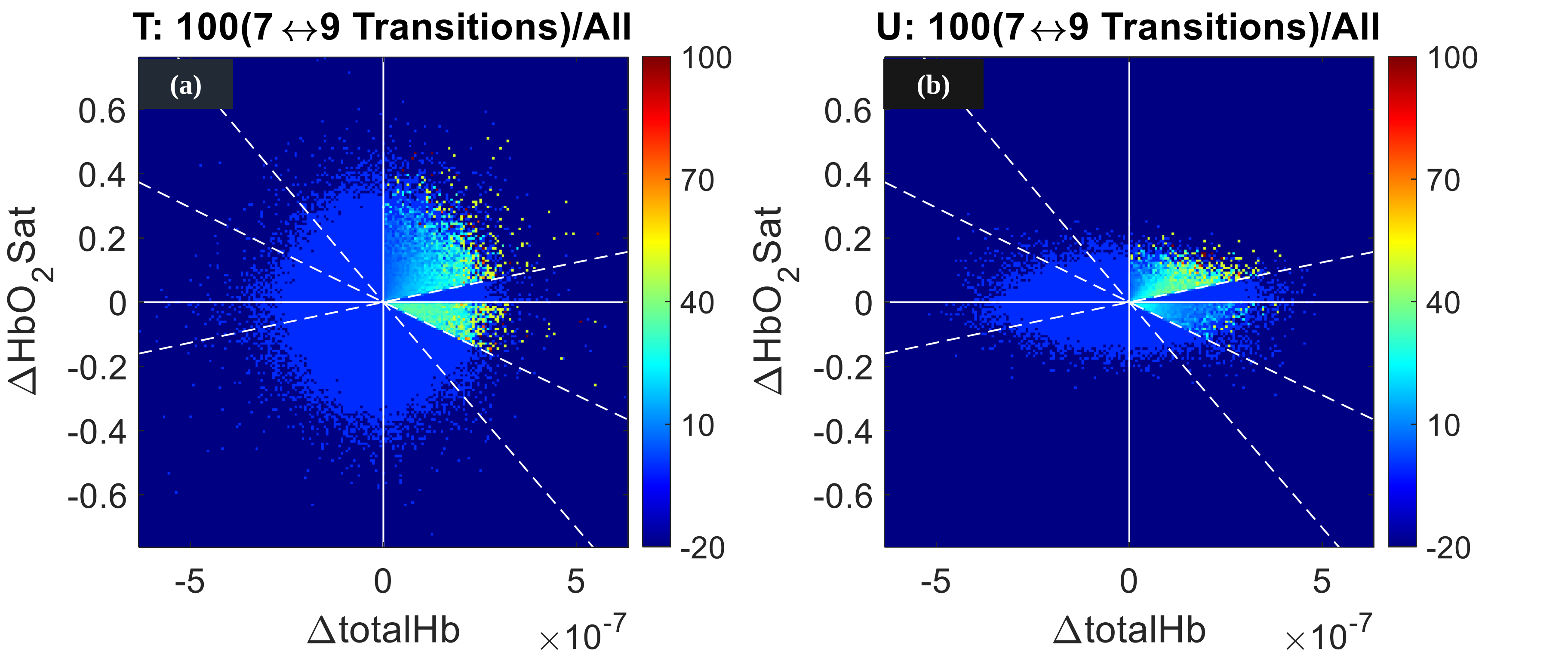

Supplementary Figure 11. Percentage of paired resting-state ΔtotalHb and ΔHbO_2Sat values that take part in either 7→9 or 9→7 transitions, for the affected (**a**) and unaffected (**b**) breasts of the same subject as in Fig. 1b-c.

Supplementary Figure 12

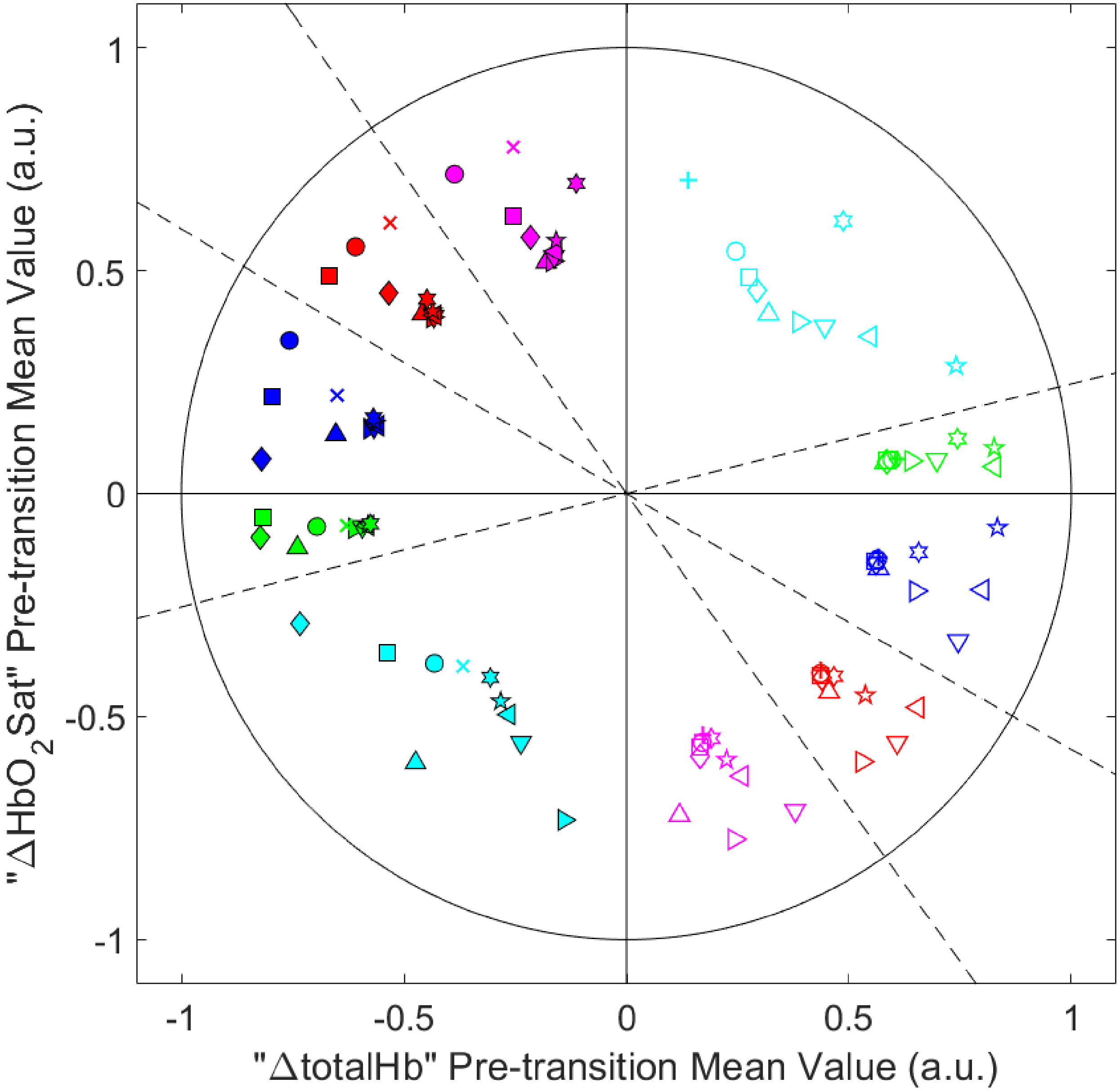

Supplementary Figure 12. Plot of pre-transition mean-value coordinates, computed for the uniform-disk model using Eq. (S5.2) with $\alpha = 3$. Model result is qualitatively different from the Fig. 3a breast-data counterpart.

Supplementary Figure 13

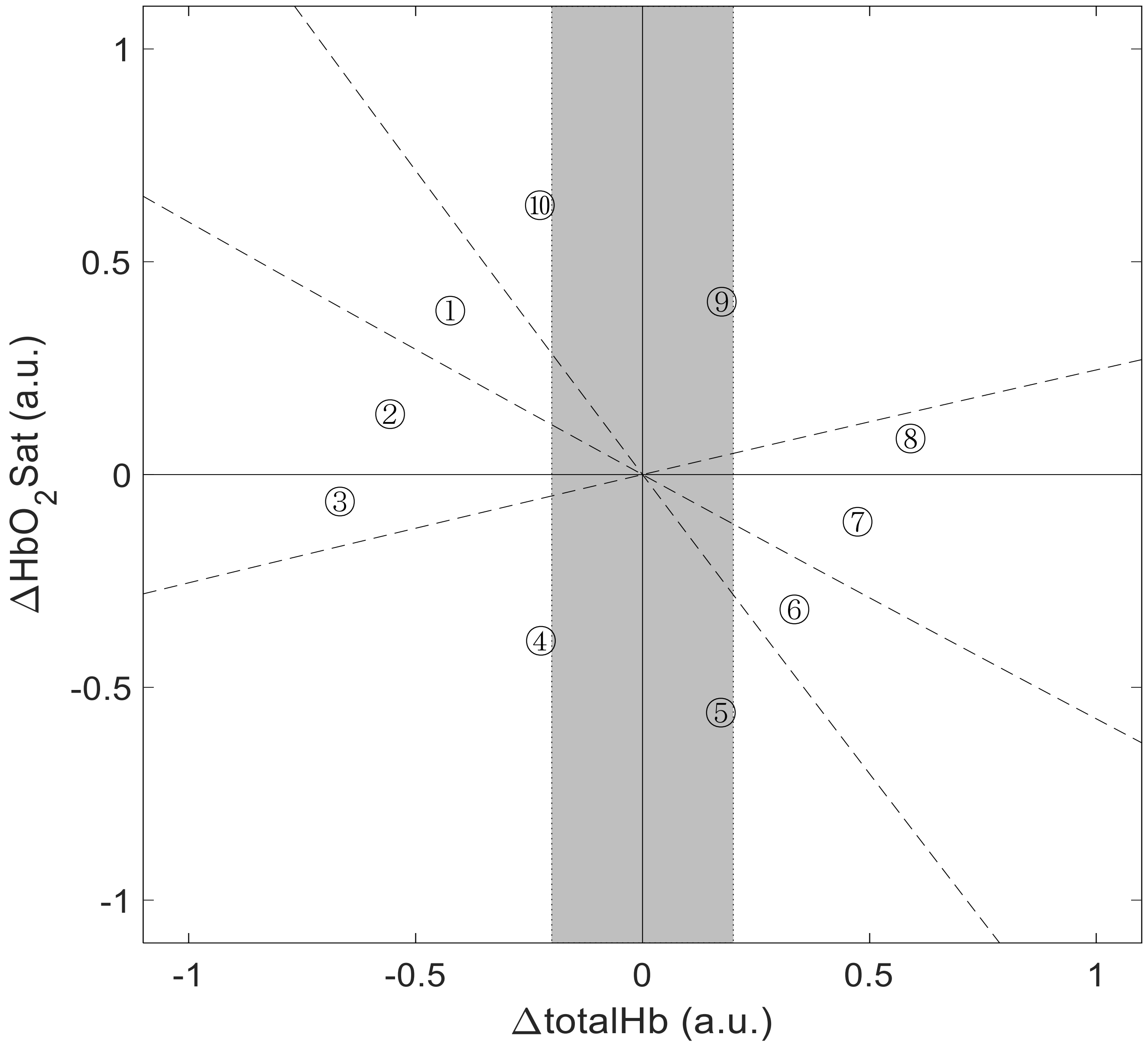

Supplementary Figure 13. The ΔHbO_2Sat vs. ΔtotalHb coordinate system, with State sectors explicitly labeled with circled numbers. The region |ΔtotalHb ≤ 0.2| is colored gray, showing that it occupies finite-area regions in States 1-3 and 6-8, but (in principle) infinite-area regions in States 10, 4, 5 and 9.

Supplementary Figure 14

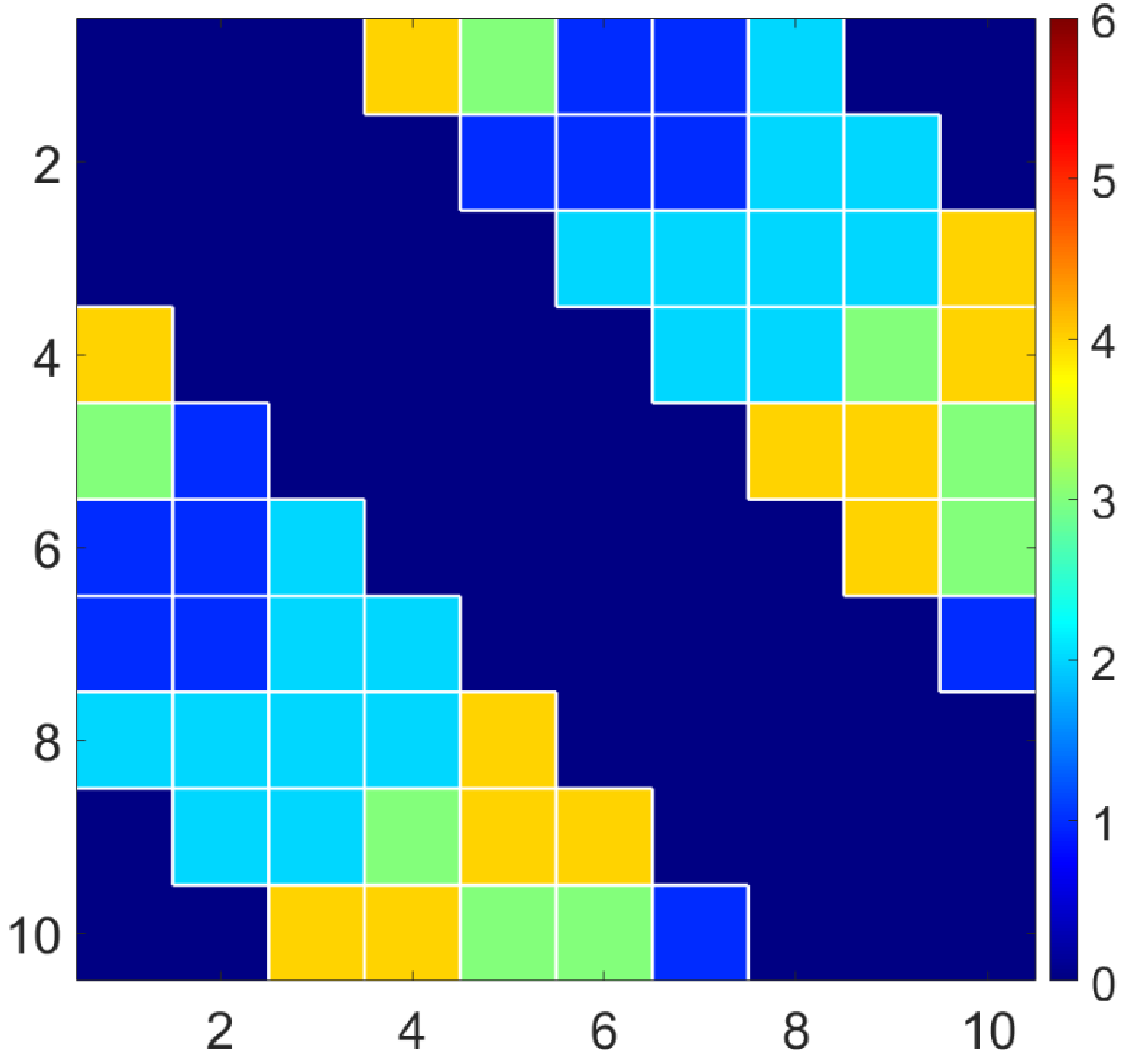

Supplementary Figure 14. Colormap showing the subsets of Class 3-5 transition types in each of the rank-order categories considered in Table 2 (Supplementary Note 15): 1 = |*t*′(ΔtotalHb)| > |*t*′(ΔHbO_2Sat)| > |*t*′(Δ(dwell time))|, 2 = |*t*′(ΔtotalHb)| > |*t*′(Δ(dwell time))| > |*t*′(ΔHbO_2Sat)|, 3 = |*t*′(ΔHbO_2Sat)| > |*t*′(ΔtotalHb)| > |*t*′(Δ(dwell time))|, 4 = |*t*′(ΔHbO_2Sat)| > |*t*′(Δ(dwell time))| > |*t*′(ΔtotalHb)|).

Supplementary Figure 15

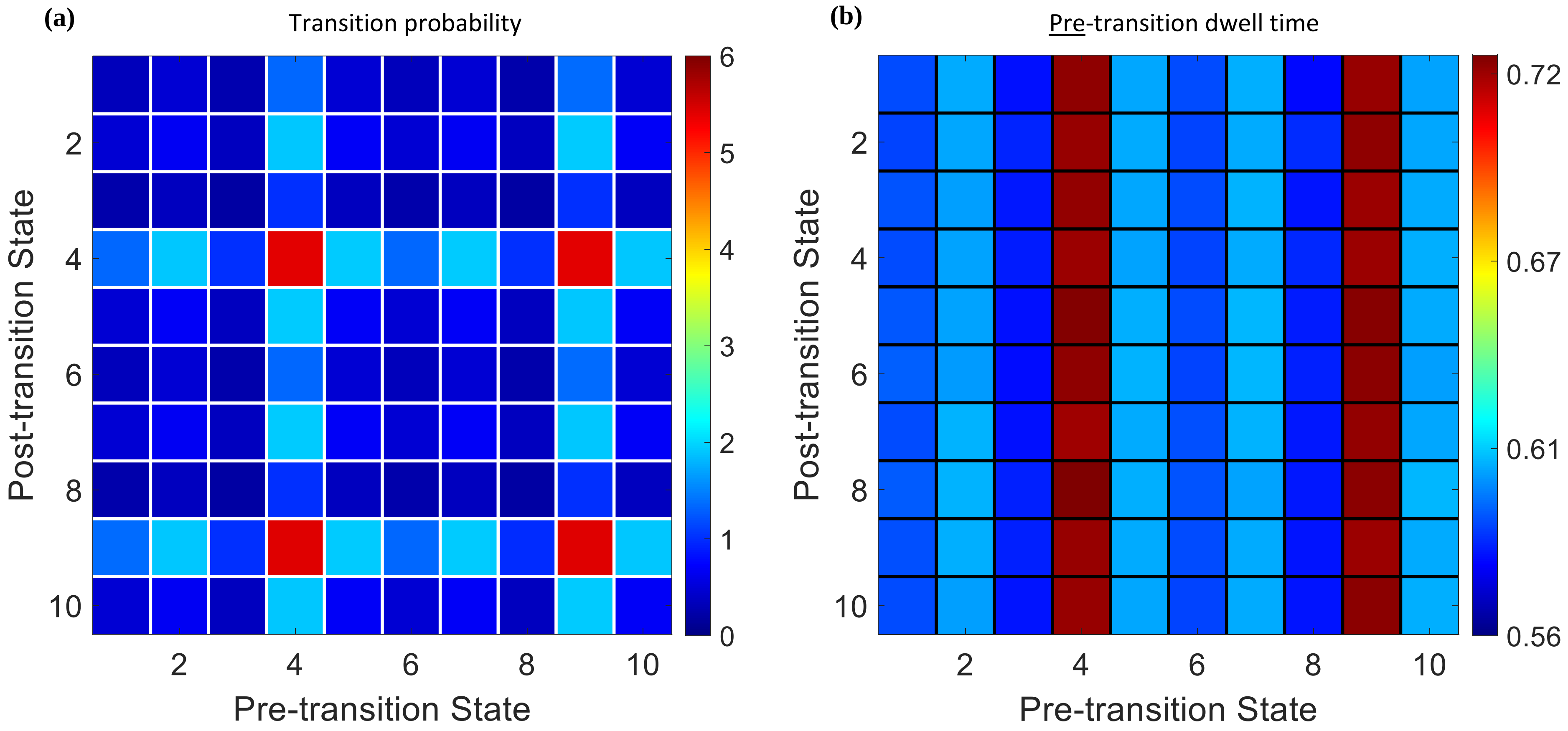

Supplementary Figure 15. Noise analogue plot of Fig. 2a,b; **a**, transition probability (%). **b**, pre-transition dwell time (sec).

Supplementary Figure 16

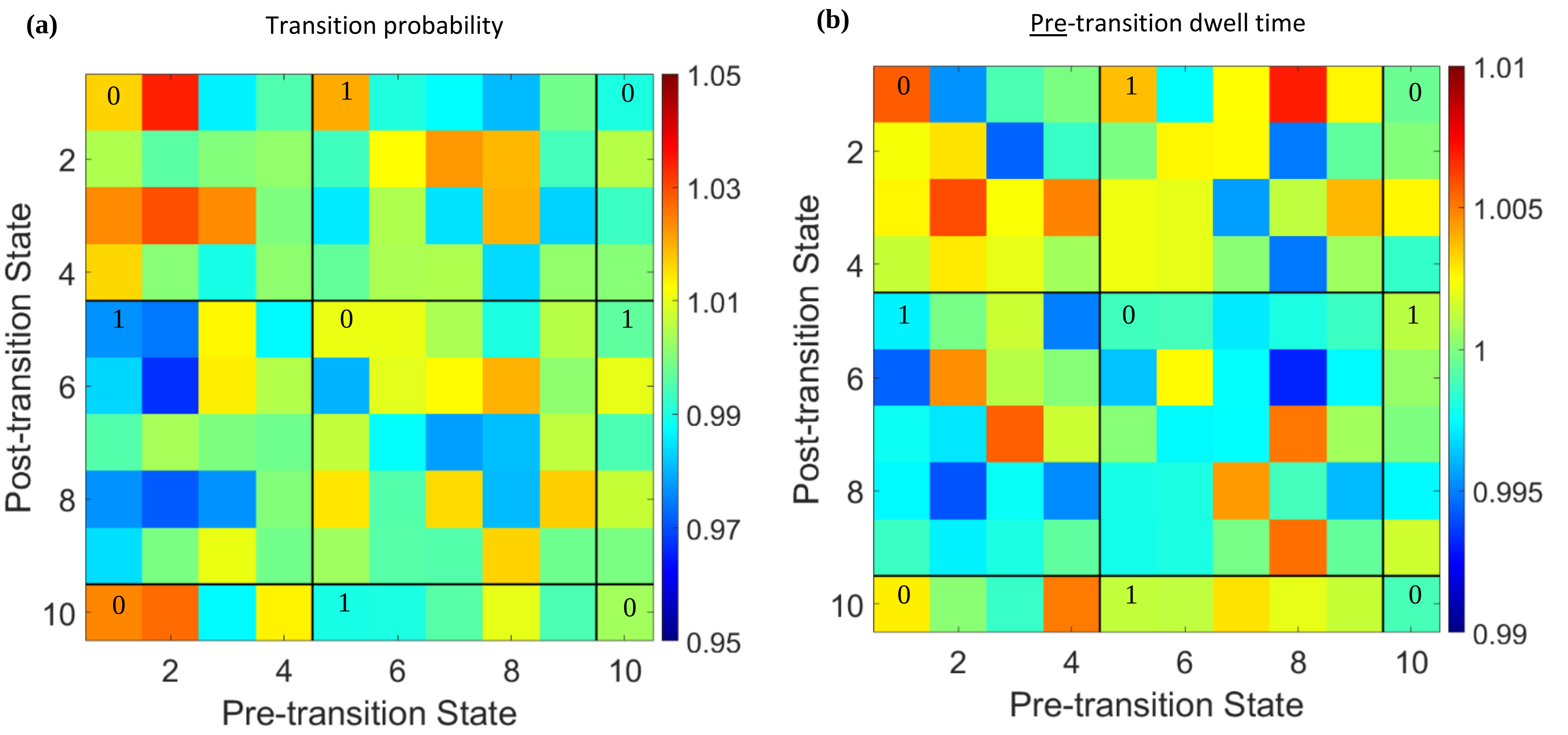

Supplementary Figure 16. Noise analogue plot of Supplementary Fig. 3a-b. Ratios of noise-derived adjacency-matrix values, for pairs of transition types chosen so that every signal component undergoes a sign change in one transition type and is unchanged in the other. **a**, Ratios of transition probabilities. **b**, ratios of pre-transition dwell times. Thin horizontal and vertical lines separate the 10×10 matrix into transition types for which the algebraic sign of ΔtotalHb does (‘1’) and does not (‘0’) change.

Supplementary Figure 17

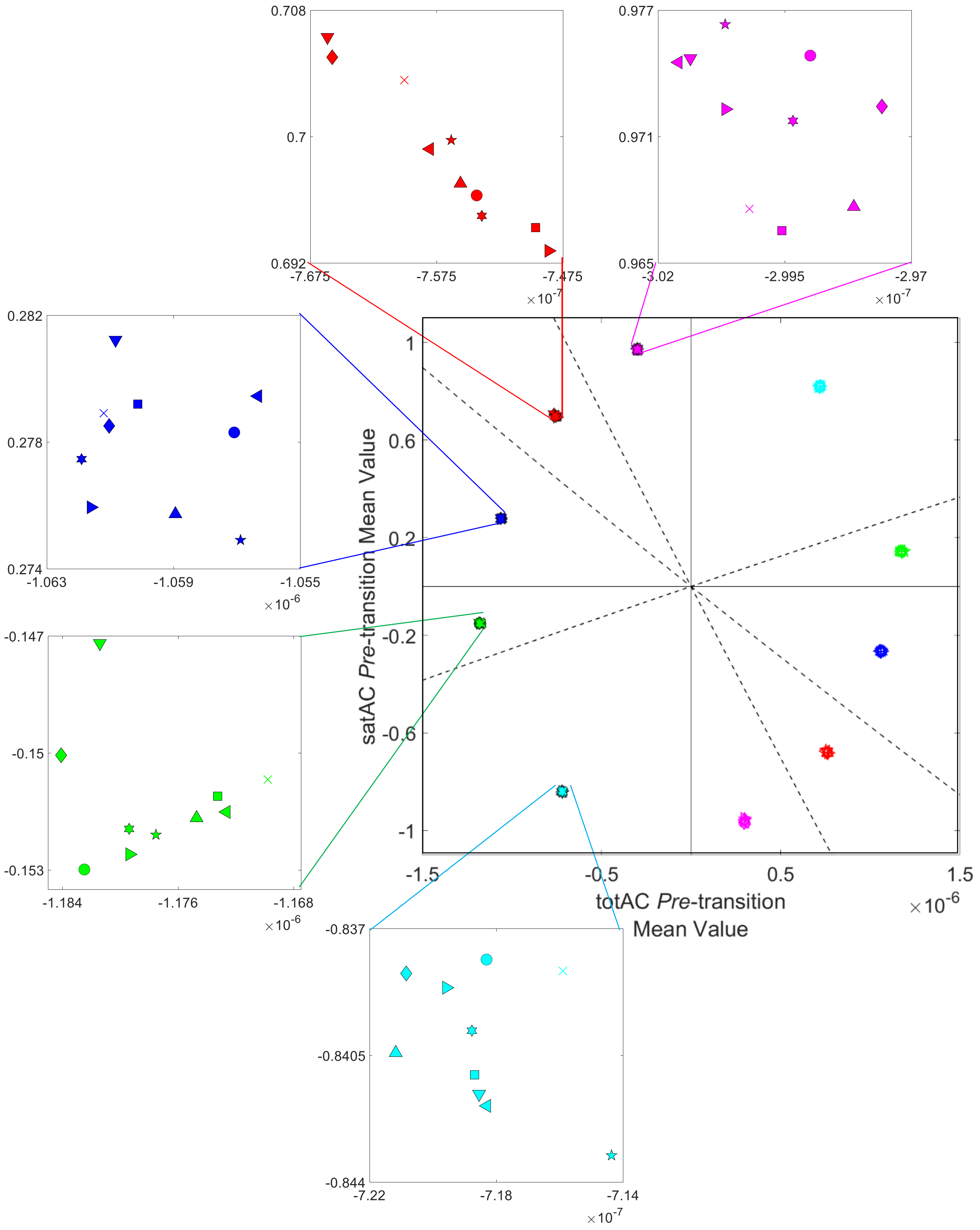

Supplementary Figure 17. Noise analogue plot of Fig. 3a. Noise data-based mean pre-transition satAC vs. mean pre-transition totAC. In the original-scale view, every set of 10 points for a fixed pre-transition State (i.e., having the same combination of symbol fill and color) appears co-located. Magnified views of the one-State clusters reveal no consistent structure.

Supplementary Figure 18

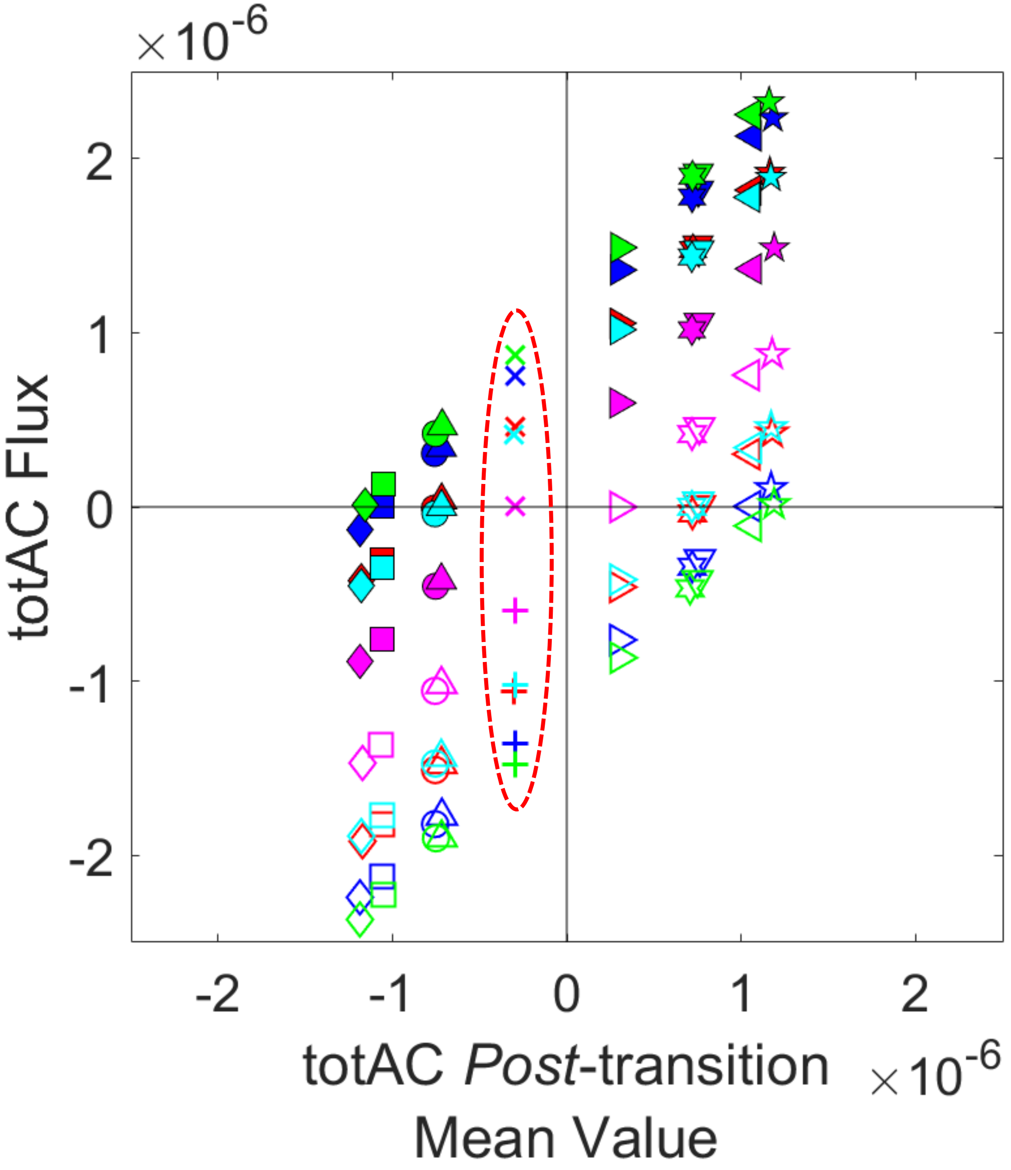

Supplementary Figure 18. Noise analogue plot of Fig. 3d; Noise data-based totAC flux vs. mean post-transition totAC. Every set of 10 points for a fixed pre-transition State (i.e., having the same combination of symbol fill and color) or fixed post-transition State (i.e., having the same symbol shape) lie on a straight line. The specific case of post-transition State 10 is highlighted (red dashed ellipse), for concreteness.

Supplementary Figure 19

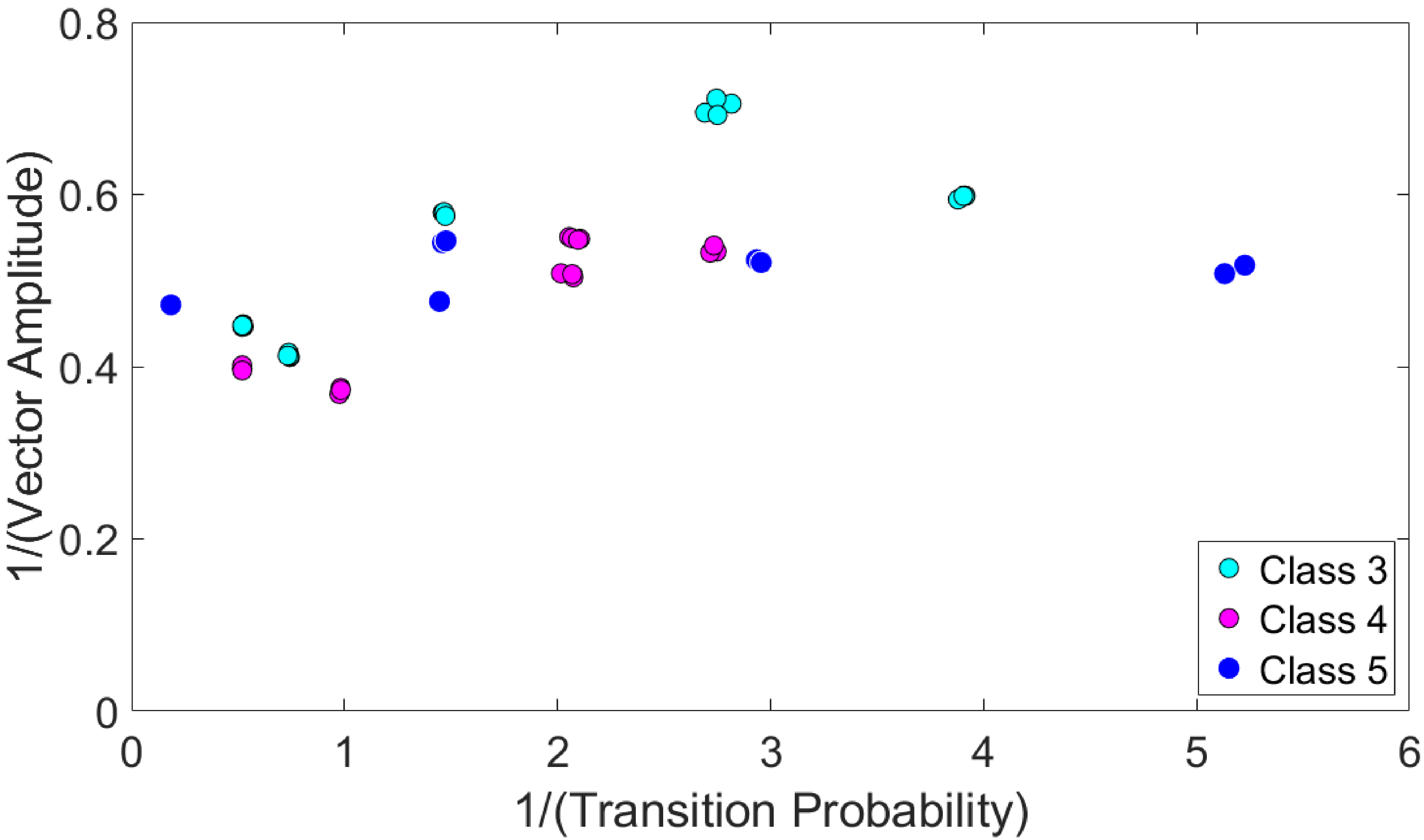

Supplementary Figure 19. Noise analogue plot of Fig. 5d; L-B plot computed from noise-simulation data.

Supplementary Figure 20

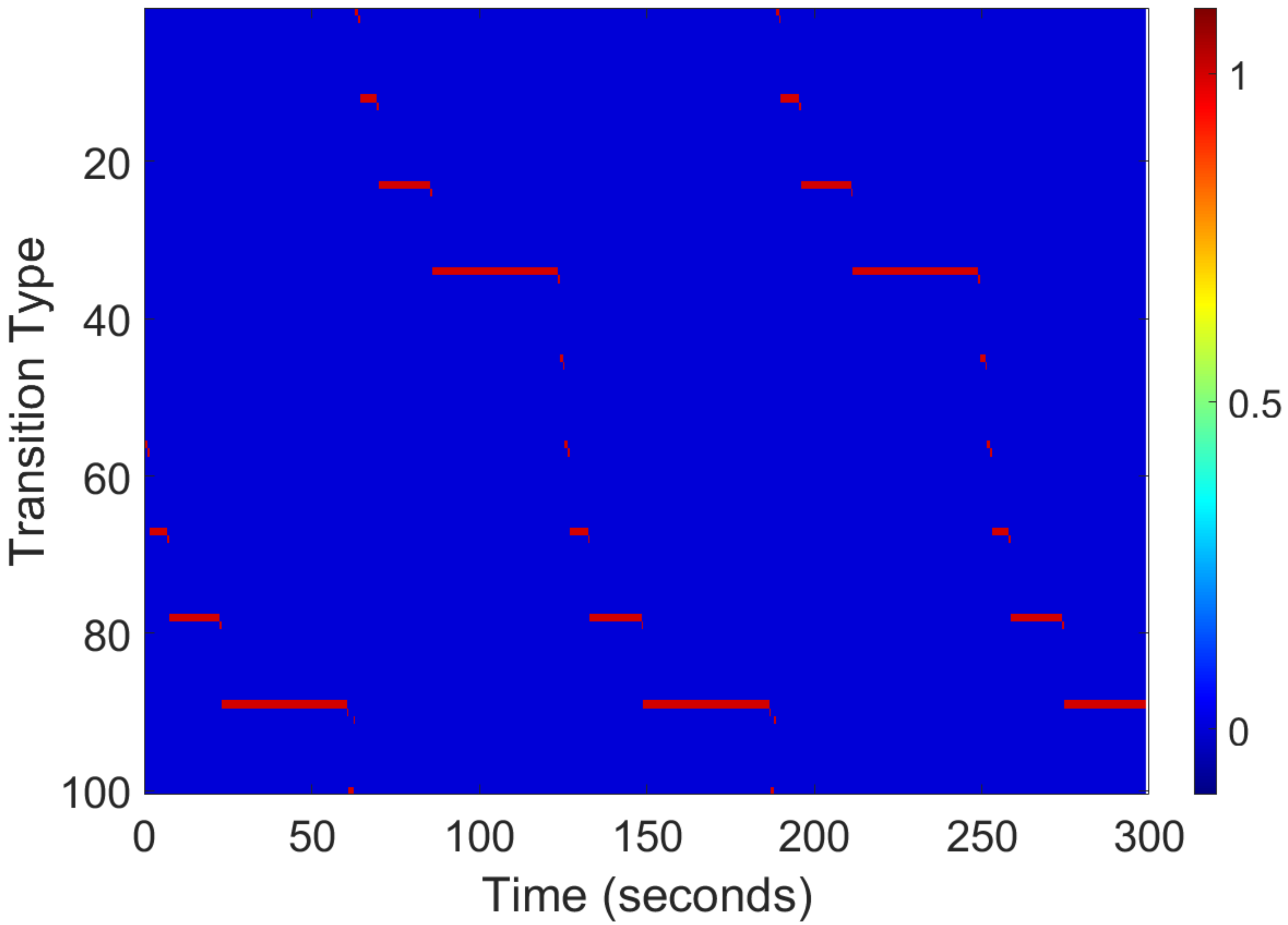

Supplementary Figure 20. Transition count matrix **C** (Eq. (3)) for a set of perfectly sinusoidal simulated hemodynamic time series. Row dimension is transition type as defined in Eq. (1), column dimension is the measurement time step (in units of seconds), and the transition count is indicated by the color.

Supplementary Figure 21

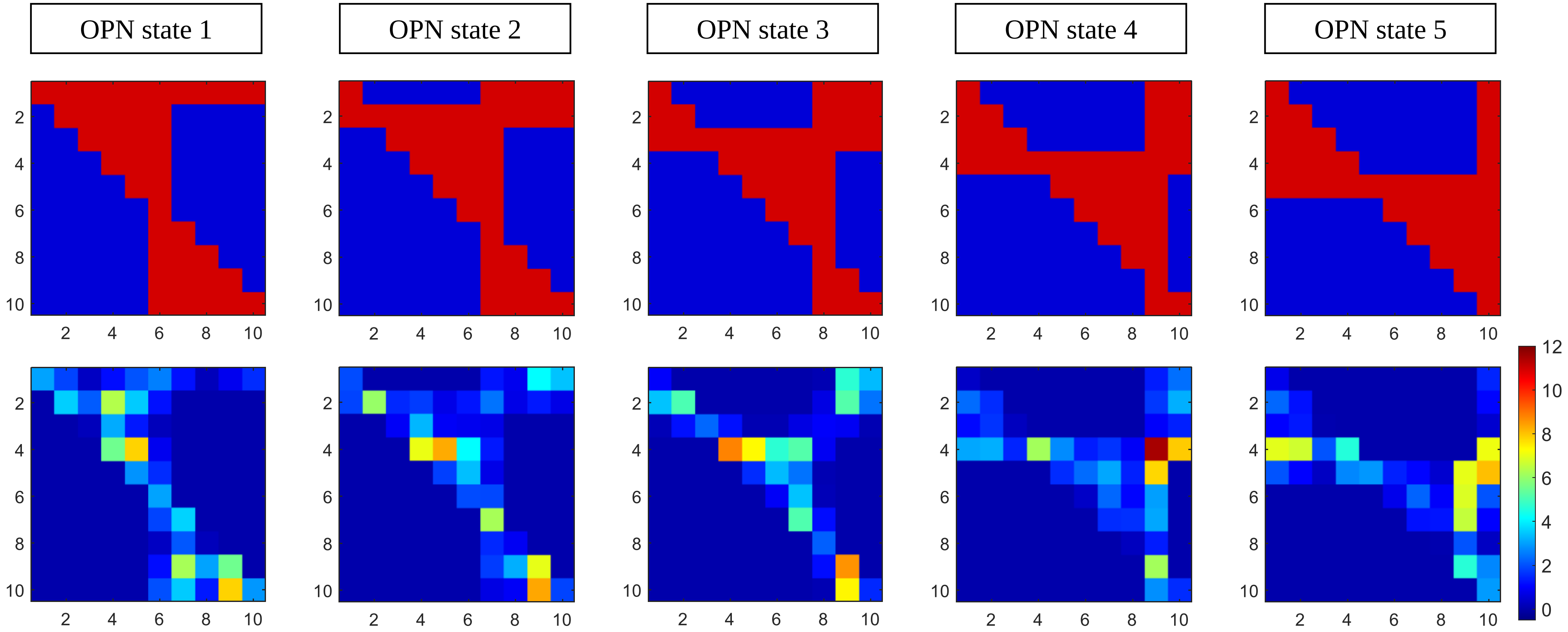

Supplementary Figure 21. Distribution of transition types for the States-transitions network (fr-OPN) within multivariate OPN states 1-5. Corresponding data for states 6-10 (not shown) are the transposes of the states 1-5 findings. Colormaps in the top row show the transition types that do (red; n = 39 in every case) and do not (blue) contribute to the specified states. The bottom row shows the percentage of state-j events (j = 1-5) that are from each transition type. In every panel the plotted data are averages of left- and right-breast values for the 45 non-cancer subjects. As in Fig. 2 and Supplementary Fig. 22, matrix column and row indices denote the pre- and post-transition States, respectively.

Supplementary Figure 22

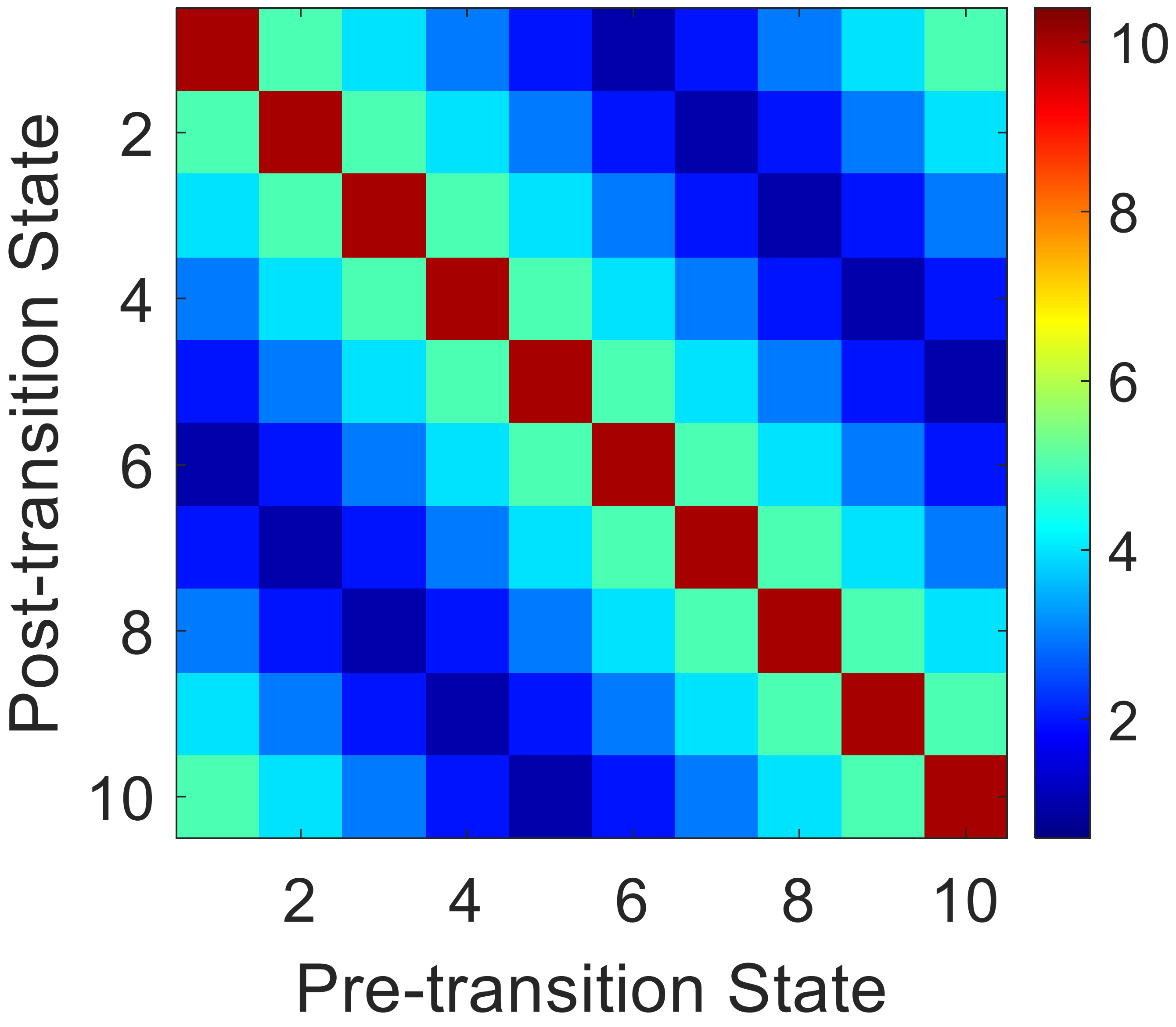

Supplementary Figure 22. Colormap showing the number of multivariate OPN states that include transitions from each transition type of the States-transitions network.

Supplementary Figure 23

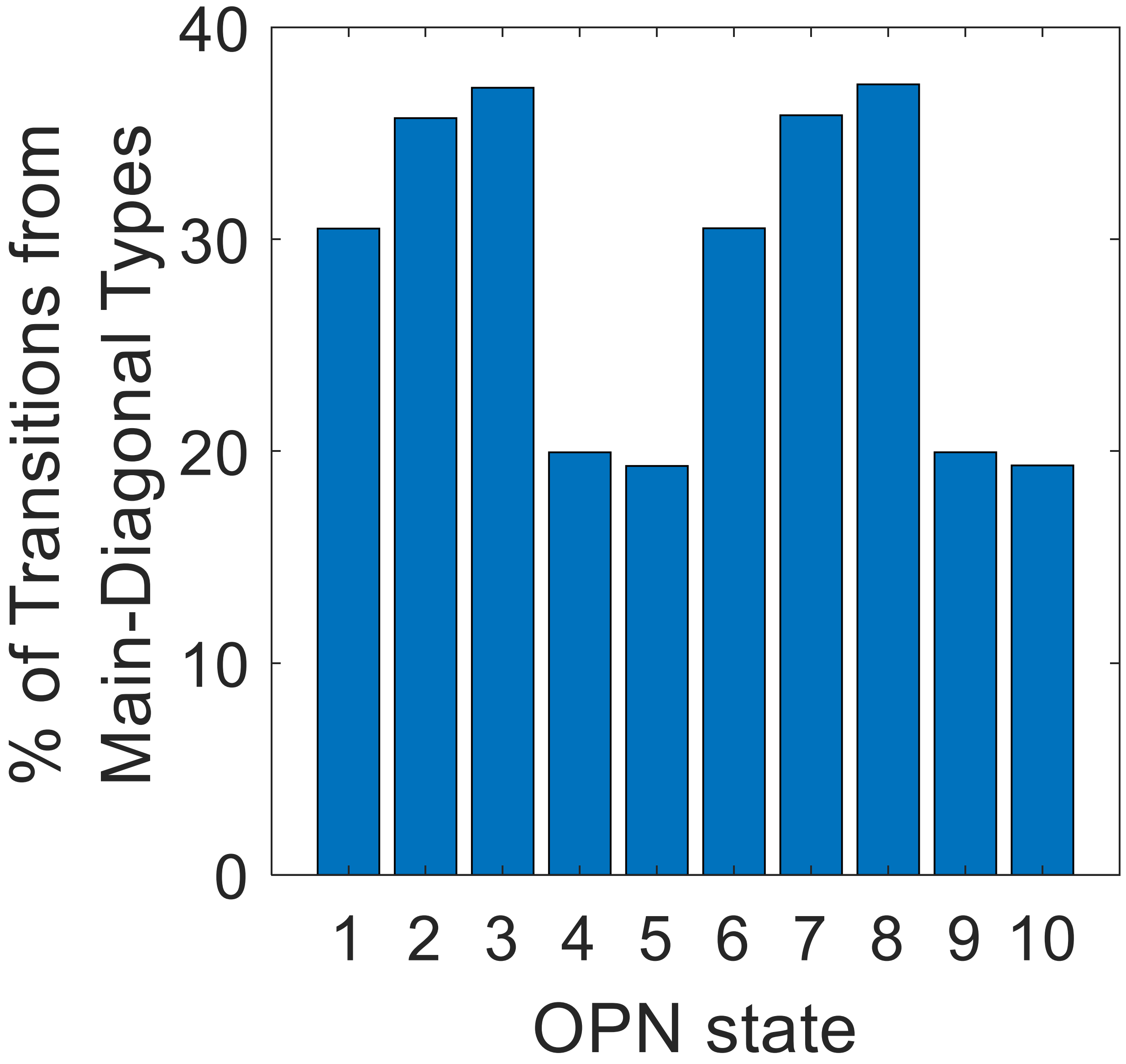

Supplementary Figure 23. Bar graph showing the percentage of all individual transitions in each OPN state that are accounted for by the Class-0 transition types.

Supplementary Tables

Supplementary Table 1: Findings reported here involve exploration of data for the "Second-generation imager" subject group [1,21], while different data analysis methodologies are applied here. Subject demographic information is summarized in Table S1.

Table S1. Subject-group descriptive information.

Group-Level Parameter	Active Breast Cancer		Benign Breast Pathology	No Breast Pathology
	Left	Right		
N	12[(a]	6[(b,c]	23	22
Age [yr, mean (SD)]	53.9 (9.5)	53.7 (14.1)	48.1 (10.7)	51.5 (11.7)
BMI [$kg\text{-}m^{-2}$, mean (SD)]	32.6 (8.6)	31.8 (6.2)	31.6 (6.4)	29.9 (4.5)
Tumor Size [cm, min-max (mean)]	0.5-6 (2.8)	1-5 (2.7)	n/a	n/a

a) Includes 10 cases of invasive ductal carcinoma (IDC), 1 of invasive lobular carcinoma, and 1 of invasive mammary carcinoma (ImaC).

b) Includes 4 cases of IDC, 1 of ImaC and 1 of invasive mucinous carcinoma.

c) Includes one equivocal case: subject had a right-breast lumpectomy (IDC) 2 years prior to NIRS study, and subsequent radiological scans suggested a recurrence.

Table S2. Algebraic sign-change properties for transitions from pre-transition State 4.

Post-transition State	Presence/absence of transition-associated algebraic-sign change[a]				
	ΔdeoxyHb	ΔHbO_2Exc	ΔoxyHb	ΔHbO_2Sat	ΔtotalHb
1	+	+	-	+	-
2	+	-	-	+	-
3	+	-	-	-	-
4	-	-	-	-	-
5	-	-	-	-	+
6	-	-	+	-	+
7	-	+	+	-	+
8	-	+	+	+	+
9	+	+	+	+	+
10	+	+	+	+	-

a) A '+' in the table may denote either a change from a negative (i.e., less than the baseline mean) pre-transition value to a positive post-transition one, or a positive-to-negative change. A '-' may denote either a positive-to-positive or a negative-to-negative transition. Pairs of table rows with the same color denote transition types having opposite algebraic sign-change properties for all five Hb-signal components.

Table S3. Numbers of transition types with statistically significant network-coefficient values.[a]

Network Coefficient[a]	Number of Transition Types with $p < 10^{-4}$ [c]
ΔtotalHb Flux	99
ΔHbO_2Sat Flux	95
$\tau^{(2)} - \tau^{(1)}$ (Dwell-Time "Flux")	93
Transition Probability	95

a) Results of the surrogate-data analysis described in Supplementary Note 16 are shown, for a selected subject.

b) Tabulated quantities are the network coefficients that are used in generating a Lineweaver-Burk plot (e.g., Fig. 5d).

c) With unperturbed network-coefficient values, surrogate-data means, and surrogate standard deviations denoted as U, M_s, and D_s, respectively, the t-test statistic is $t = (M_s - U)/D_s$, and the null hypothesis in $t = 0$.

Supplementary References

1. Barbour, R.L. et al. Hemoglobin state-flux: A finite-state model representation of the hemoglobin signal for evaluation of the resting state and the influence of disease. *PLoS ONE* **13**, e0198210 (2018).

2. List of centroids: 2-D Centroids. Retrieved October 20, 2022: https://en.wikipedia.org/wiki/List_of_centroids#2-D_Centroids.

3. Distance from a point to a line: Cartesian coordinates. Retrieved October 20, 2022: https://en.wikipedia.org/wiki/Distance_from_a_point_to_a_line#Cartesian_coordinates.

4. Wallin, A.E. Uniform random points in a circle using polar coordinates. Retrieved October 20, 2022: http://www.anderswallin.net/2009/05/uniform-random-points-in-a-circle-using-polar-coordinates/.

5. Grinshpan, A. Permutation matrices. Retrieved October 20, 2022: https://www.math.drexel.edu/~tolya/permutations.pdf.

6. Cyganek, B. *Object Detection and Recognition in Digital Images: Theory and Practice* (Wiley, 2013).

7. McLogan, B. Writing the equation of a hyperbola given the foci and vertices. Retrieved October 19, 2022: https://www.youtube.com/watch?v=mY8-rDNx0BI.

8. Barbour, R.L. et al. Hb State-Flux measures yield disease-sensitive Michaelis-Menten type behaviors. Poster #2125 at 2020 OHBM Annual Meeting.

9. Lehners, M. cGMP signaling and vascular smooth muscle cell plasticity. *J. Cardiovasc. Development and Disease* **5**, 20 (2018).

10. Choudhari, S.K. et al. Nitric oxide and cancer: A review. *World J. Surg. Oncol.* **11**, 118 (2013).

11. Stone, J.R. & Marletta, M.A. Spectral and Kinetic Studies on the Activation of Soluble Guanylate Cyclase by Nitric Oxide. *Biochem.* **35**, 1093-1099 (1996).

12. Yuan, A.E. & Shou, W. Data-driven causal analysis of observational biological time series. *eLife* **11**, e72518 (2022).

13. Al abdi, R. et al. Optomechanical imaging system for breast cancer detection. *J. Opt. Soc. Am. A* **28**, 2473-2493 (2011).

14. Commin, H. Generating Partially Correlated Random Variables. Retrieved October 19, 2022: https://www.dsprelated.com/showarticle/1241.php.

15. Varley, T.F. & Sporns, O. Network analysis of time series: Novel approaches to network neuroscience. *Frontiers in Neurosci.* **15**, 787068 (2022).

16. Zhang, J. et al. Constructing ordinal partition transition networks from multivariate time series. *Scientific Reports* **7**, 7795 (2017).

17. Lacasa, L. et al. From time series to complex networks: The visibility graph. *PNAS* **105**, 4972-4975 (2008).

18. Supriya, S. et al. Weighted visibility graph with complex network features in the detection of epilepsy. *IEEE Access* **4**, 6554-6566 (2016).

19. Sannino, S. et al. Visibility graphs for fMRI data: Multiplex temporal graphs and their modulations across resting-state networks. *Network Neurosci.* **1**, 208-221 (2017).

20. Cai, Q. et al. A multiplex visibility graph motif-based convolutional neural network for characterizing sleep stages using EEG signals. *Brain Science Advances* **6**, 355-363 (2020).

21. Graber, H.L. et al. Enhanced resting-state dynamics of the hemoglobin signal as a novel biomarker for detection of breast cancer. *Med. Phys.* **42**, 6406-6424 (2015).
